# An optimized CYP3A4-activatable fluorogenic sensor for *in situ* functional imaging and multi-dimensional inhibitor assessment†

**DOI:** 10.1039/d5sc01791b

**Published:** 2025-05-23

**Authors:** Feng Zhang, Yufan Fan, Mei Luo, Jian Huang, Bei Zhao, Lin Chen, Guanghao Zhu, Yuan Xiong, Hong Lin, Chuting Xu, Xiaodi Yang, Tony D. James, Guangbo Ge

**Affiliations:** a State Key Laboratory of Discovery and Utilization of Functional Components in Traditional Chinese Medicine, Shanghai Frontiers Science Center of TCM Chemical Biology, Institute of Interdisciplinary Integrative Medicine Research, Shanghai University of Traditional Chinese Medicine Shanghai 201203 China geguangbo@shutcm.edu.cn; b Pharmacology and Toxicology Division, Shanghai Institute of Food and Drug Control Shanghai 201203 China; c The Research Center of Chiral Drugs, Innovation Research Institute of Traditional Chinese Medicine, Shanghai University of Traditional Chinese Medicine Shanghai 201203 China; d School of Chemistry and Chemical Engineering, Henan Normal University Xinxiang 453007 China; e Department of Chemistry, University of Bath Bath BA2 7AY UK t.d.james@bath.ac.uk

## Abstract

Cytochrome P450 3A4 (CYP3A4), one of the most important drug-metabolizing enzymes, plays a pivotal role in the oxidative metabolism of a wide range of non-polar xenobiotics and endogenous substances. Deciphering the dynamic changes in CYP3A4 activity under specific physiological or pathological conditions, as well as assessing the modulatory effects of therapeutic agents on CYP3A4, requires highly-efficient and reliable tools for sensing CYP3A4 activity within complex biological matrices. Herein, an integrated strategy was adopted for developing an optimized CYP3A4-activatable fluorogenic sensor that enables *in situ* detection of CYP3A4 activity in living systems without the interference of P-glycoprotein (P-gp), *via* integrating computer-aided substrate design, drug-likeness filtering, and biochemical assays. Following screening a range of 1,8-naphthalimide derivatives, *N*-cyclopropylmethyl-1,8-naphthalimide (NCN) was identified as an optimized fluorogenic substrate for CYP3A4, demonstrating exceptional isoform-specificity, single metabolite formation, ultrahigh sensitivity, high binding-affinity, improved cell-membrane permeability, and favorable bio-safety profiles. Notably, both NCN and its fluorogenic metabolite (HNCN) were identified as non-substrates of P-gp, which greatly facilitated *in situ* functional imaging of CYP3A4 activities in living systems, such as live cells and organs. It was also found that NCN was an orally bioavailable agent, which significantly facilitated the precise assessment of CYP3A4 inhibitors across multi-dimensional biological systems, including *in vitro*, *ex vivo*, and *in vivo*. Collectively, this work showcases an integrated strategy for the rational engineering of isoform-specific and orally bioavailable CYP3A4-activatable fluorogenic substrates for CYP3A4, with NCN emerging as a practical and reliable CYP3A4-activatable tool for *in situ* imaging and inhibitor assessment.

## Abstract 2

An integrated molecular engineering strategy was developed for constructing a highly specific and orally active CYP3A4-activatable fluorogenic sensor for CYP3A4, offering a practical and reliable fluorogenic tool for *in situ* imaging and inhibitor assessment in various biological systems.

## Graphical abstract

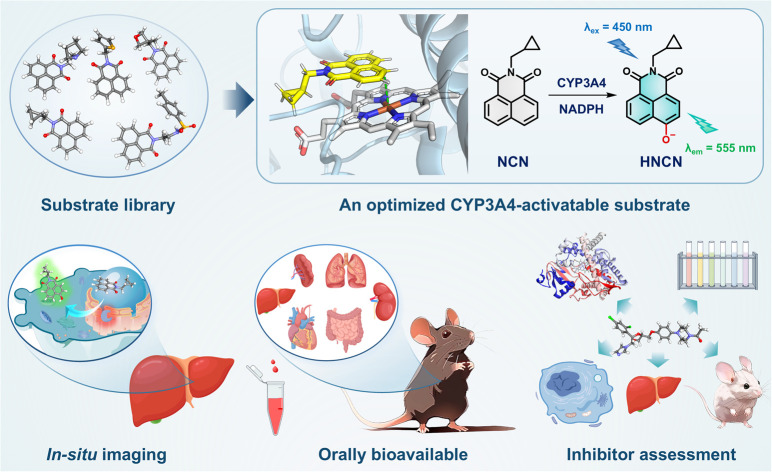

## Introduction

Cytochrome P450 enzymes (CYPs) are a diverse superfamily of metabolic enzymes that play a crucial role in the oxidative metabolism of a wide range of endogenous substances (*e.g.*, steroids, bile acids, fatty acids) and xenobiotics (*e.g.*, drugs, food chemicals, and environmental toxins).^1–3^ Among all identified human CYPs, CYP3A4 is one of the most important and well-investigated members,^4,5^ owing to its critical roles in drug metabolism, steroid biosynthesis, drug toxicity, and drug–drug interactions.^4,6–8^ As the most abundant CYP isoenzyme in both the liver and small intestine, CYP3A4 is recognized as a major contributor to drug metabolism and a key regulator of treatment outcomes for most clinical drugs.^9,10^ Potent inhibition of CYP3A4 can significantly alter the pharmacokinetic and pharmacodynamic behavior of its substrate drugs.^11,12^ This mechanism may enhance the therapeutic efficacy of certain CYP3A4 substrate drugs (*e.g.*, nirmatrelvir and lopinavir),^13–15^ but also precipitate adverse effects or harmful drug interactions, especially when combined with drugs exhibiting a narrow therapeutic index, such as warfarin, cyclosporine, tacrolimus, theophylline.^16–19^ Thus, accurately assessing the interactions between CYP3A4 and therapeutic agents in biological systems is crucial for ensuring the safe and effective use of medications, achieving optimal therapeutic outcomes, and avoiding drug toxicity.

It is well-known that the expression and activity of CYP3A4 can be modulated by multiple factors, including genetic variations, disease states, age, gender, dietary components, drug exposure, and environmental factors.^20,21^ Under specific pathological conditions, particularly those diseases impacting liver function (*e.g.*, hepatitis, cirrhosis, and drug-induced liver injury), as well as systemic inflammatory states, the expression and activity of CYP3A4 can undergo significant alterations.^22–24^ Studies have demonstrated that the expression and activity levels of CYP3A4 are markedly reduced in various liver diseases, such as non-alcoholic fatty liver disease (NAFLD), hepatocellular carcinoma (HCC), as well as drug-induced liver injury (DILI).^25–28^ Conversely, upregulation of CYP3A4 or targeted activation of its upstream nuclear receptors (*e.g.*, pregnane X receptor, PXR) can alleviate DILI and other liver disorders.^29,30^ Furthermore, a linear correlation between the reserve hepatic function and CYP3A4 activity levels in the liver has been reported, suggesting that CYP3A4 activity can serve as a reliable functional biomarker for assessing hepatic reserve function.^31^

Deciphering CYP3A4 activity under specific pathological conditions and discovering CYP3A4 inhibitors requires reliable tools for sensing CYP3A4 activity in a complex biological matrix. Over the past few decades, drug substrates (*e.g.*, midazolam) and physiological substrates (*e.g.*, testosterone) have been frequently used to quantify CYP3A4 activity by measuring the formation rates of oxidative metabolites using liquid chromatography-mass spectrometry (LC-MS/MS).^32–35^ However, these methods are restricted by time-consuming sample preparation, expensive instrumentation, and the need for professional operators.^36,37^ In contrast, enzyme-activatable fluorogenic sensors are powerful tools for visualizing spatial distribution and functional variation of target enzyme(s), due to their inherent advantages of ultrahigh sensitivity, rapid response, high-throughput detection, and unparalleled spatiotemporal resolution.^38–43^ However, only two CYP3A4-specific fluorescent sensors, including *N*-ethyl-1,8-naphthalimide (NEN) and *N*-(4-fluorobenzyl)-1,8-naphthalimide (F8), have been reported to date (Table S1†).^44,45^ Although the two sensors excel in sensing CYP3A4 activity at microsomal or enzyme levels, their applications in living systems are strongly hindered by their susceptibility to efflux by P-glycoprotein (P-gp), a known efflux transporter showing highly overlapped substrate spectra with CYP3A4.^46–48^ This defect significantly diminishes the intracellular exposure and oral bioavailability of CYP3A4-activatable probes, thereby limiting biological applications in live cells and *in vivo*. Therefore, optimizing CYP3A4-activatable sensors that circumvent P-gp interference is crucial for sensing and imaging CYP3A4 in complex living systems, including living cells, organs, and *in vivo*.

**Scheme 1:**
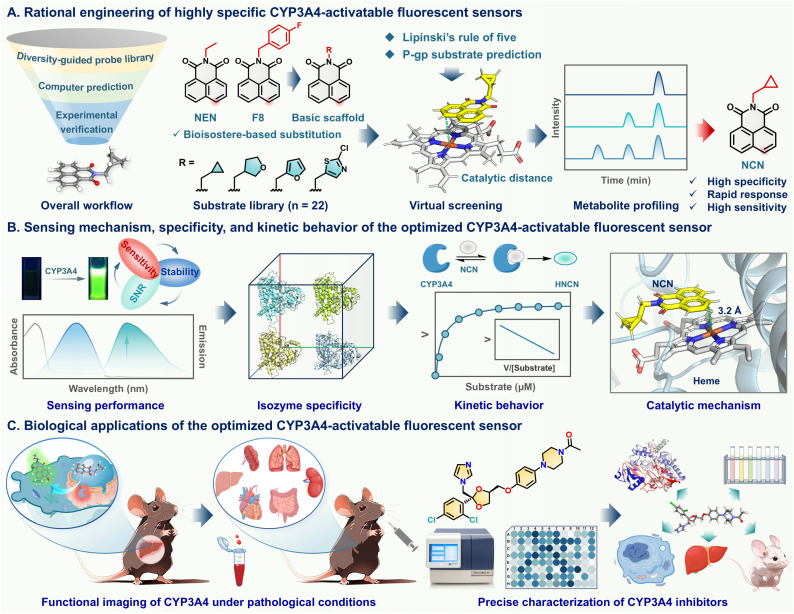
Schematic illustration of the rational engineering of CYP3A4-activatable sensors for *in situ* functional imaging and inhibitor assessment. (A) Design principle and multidimensional screening of highly specific and orally active CYP3A4 substrates. (B) Sensing performance and methodological validation of candidate probes. (C) Applications of NCN in functional imaging and precise assessment of CYP3A4 inhibitors in multi-dimensional biological systems.

Herein, an integrated strategy was adopted for developing an optimized CYP3A4 fluorogenic substrate free from P-gp interference, assembled using computer-aided substrate design, drug-likeness filtering, and biochemical validation. Firstly, a suite of NEN derivatives was designed *via* intentionally introducing various drug-like moieties on the north part of NEN to ensure their physicochemical properties adhered to Lipinski's rule of five (Scheme 1A). After that, the potential of the NEN derivatives as CYP3A4 substrates were assessed one by one using docking simulations, the top ten candidates were then synthesized for further biochemical evaluation. Among the synthesized candidates, NCN was selected as the optimal CYP3A4 substrate, since this agent exhibited a rapid metabolic rate, single metabolite formation, high isoform-specificity, ideal Michaelis–Menten kinetic behavior, and negligible P-gp efflux susceptibility (Scheme 1B). These observations indicate that NCN is an ideal CYP3A4-specific fluorescent substrate, which encouraged us to use this CYP3A4-activatable probe for the *in situ* functional imaging of this key enzyme in living systems under normal and hepatocyte injury conditions, as well as for the precise assessment of the inhibitory effects of CYP3A4 inhibitors in complex biological matrices (Scheme 1C).

## Results and discussion

### Rational engineering of a highly specific CYP3A4-activatable fluorescent probe

Practical and reliable CYP3A4-activatable fluorescent substrates suitable for precisely sensing CYP3A4 activity in complex biological systems should meet the following requirements: excellent isoform specificity, rapid response, suitable kinetic behavior (*e.g.*, Michaelis–Menten kinetics), negligible P-gp efflux liability, improved cell-membrane permeability, high bio-safety profiles, and acceptable oral bioavailability. Our previous studies have revealed that CYP3A4 prefers to catalyze 4-hydroxylation of NEN and its derivatives.^44,45^ However, NEN showed very low binding affinity towards CYP3A4 and poor cell-membrane permeability, while introducing a benzene ring on the north part of NEN (such as *N*-(4-fluorobenzyl)-1,8-naphthalimide, also termed F8) strongly enhanced the binding affinity towards CYP3A4 but such modification made F8 a good substrate for P-gp (Fig. S1†). Therefore, it is necessary to employ smarter molecular design strategies to develop more practical CYP3A4-activatable fluorescent substrates with suitable physicochemical properties, improved cell-membrane permeability, negligible P-gp efflux liability, and acceptable oral bioavailability.

**Fig. 1:**
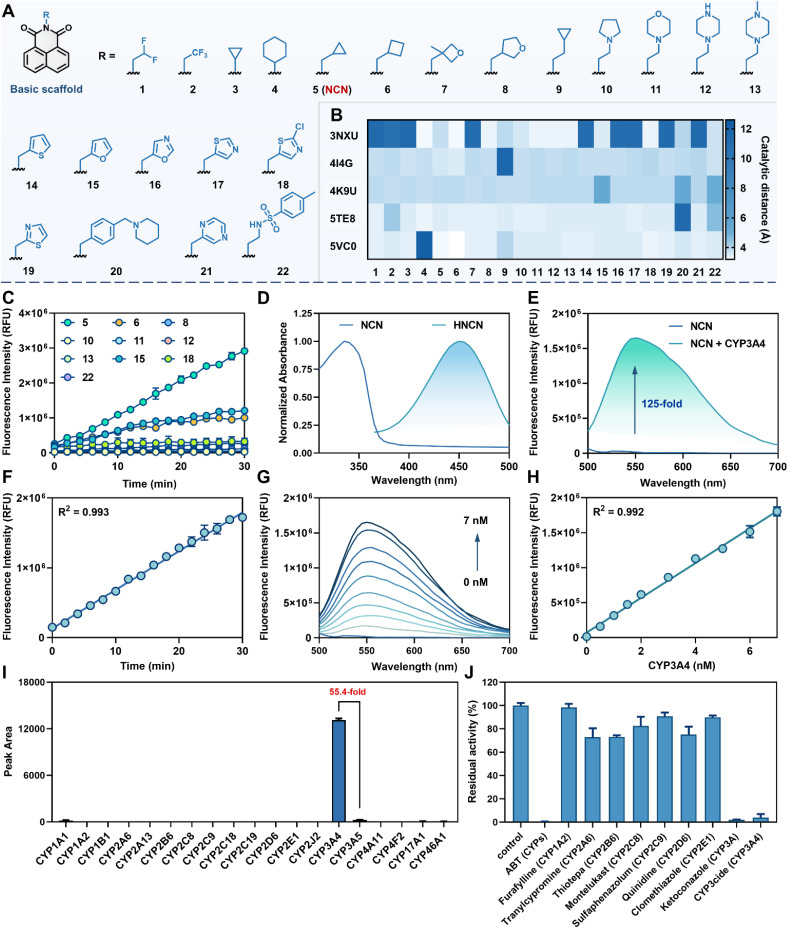
Discovery of an optimized CYP3A4-activatable fluorogenic substrate. (A) Diversity-guided CYP3A4 substrate candidate library. (B) Catalytic distances between the C-4 site of each candidate and the heme Fe atom. (C) The formation rates of the fluorescent products from candidate substrates in HLMs for 30 min. (D) Absorption spectra of NCN and HNCN. (E) Emission spectra of NCN (10 μM) in the presence or absence of CYP3A4 (2 nM) for 30 min. (F) Time-dependent response of CYP3A4-catalyzed NCN 4-hydroxylation. (G) Emission spectra of NCN (10 μM) incubated with various concentrations of CYP3A4 (0–7 nM). (H) A linear relationship between fluorescence intensity of HNCN and CYP3A4 concentration. (I) Fluorescence response of NCN (10 μM) towards various human P450 enzymes. (J) Chemical inhibition assays of NCN 4-hydroxylation in HLMs.

Herein, a bioisosterism strategy was adopted to construct a diversity-guided CYP3A4-activatable fluorescent substrate library by replacing the northern moiety of NEN with a series of drug-like moieties that exhibited similar electronic effects and physicochemical properties. To avoid interference from P-gp, a suite of non-benzene ring moieties (*e.g.*, heterocyclic rings or cycloalkyl moieties) were intentionally introduced on the northern part of NEN (Fig. 1A). A total of 22 NEN derivatives were designed, based on their physicochemical properties and the obeyance of Lipinski's rule of five, including molecular weight (MW), lipophilicity (log *P*), topological polar surface area (tPSA), hydrogen bond donor (HBD), hydrogen bond acceptor (HBA), and the number of rotatable bonds (Table S2†). Meanwhile, all designed NEN derivatives were predicted to exhibit acceptable gastrointestinal absorption and negligible P-gp efflux liability (Table S3†).

After that, the potential of these NEN derivatives as CYP3A4-activatable fluorescent substrates was screened using a dual-filtering strategy that integrated computer-aided virtual screening and P450 reaction phenotyping assays. Specifically, ensemble docking simulations were adopted to determine the distance between the C-4 position of each NEN derivative and the iron atom of heme in the catalytic cavity of human CYP3A4. Given the flexible nature of the catalytic pocket of CYP3A4, five crystal structures (PDB ID: 3NXU, 4I4G, 4K9U, 5TE8, and 5VC0) were retrieved for docking simulations. As shown in Fig. 1B and Table S4,† most of the designed NEN derivatives could be well-docked into the catalytic pocket of CYP3A4 and formed catalytic conformations with distances of less than 5.0 Å. Notably, the top ten candidates (5, 6, 8, 10, 11, 12, 13, 15, 18, and 22) exhibited a low dispersion of catalytic distances, highlighting their significant potential as CYP3A4 substrates. Therefore, these top ten candidates were chemically synthesized and fully characterized (Fig. S2–S31†). Their sensing potential for CYP3A4 was then evaluated by quantifying the formation rates of the corresponding fluorescent product and the fluorescence enhancement efficacy. Among all evaluated candidates, probes 5, 6, and 15 exhibited noticeable fluorescence activation (Fig. 1C) following oxidative metabolism in human liver microsomes (HLMs). However, probe 6 and 15 could be metabolized by HLMs to generate multiple metabolites, while only probe 5 (*N*-cyclopropylmethyl-1,8-naphthalimide, also termed NCN) could be rapidly metabolized by HLMs to generate a single oxidative metabolite that emitted bright fluorescence signals at around 555 nm (Fig. S32†). These findings suggest that NCN is a good substrate candidate for sensing CYP3A4 with favorable physicochemical properties and superior reactivity.

### Sensing performance of NCN towards CYP3A4

**Fig. 2:**
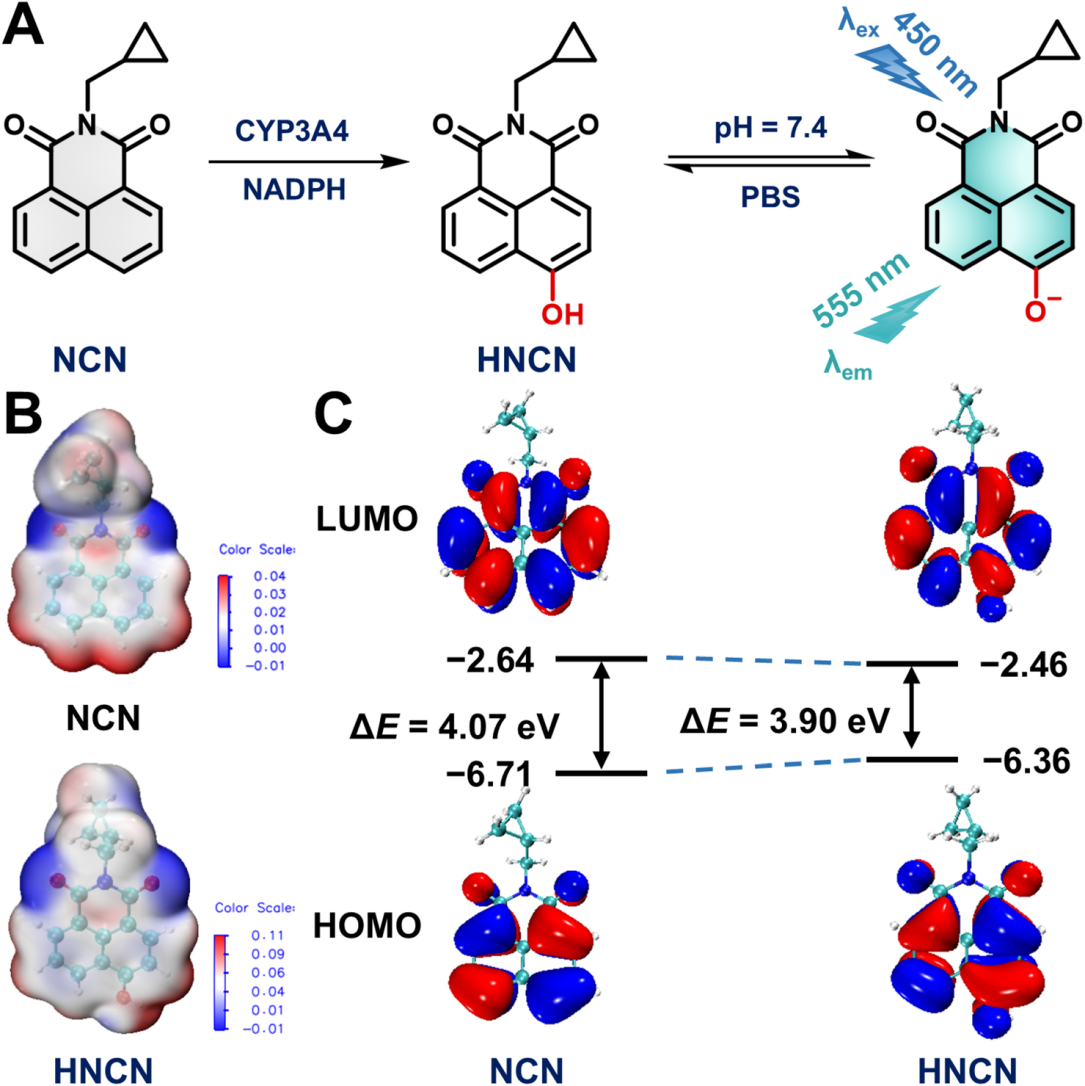
Sensing mechanism NCN for CYP3A4. (A) Proposed sensing mode of NCN toward CYP3A4. (B) ESP distribution of NCN and HNCN. (C) Frontier molecular orbitals and energy gaps of NCN and HNCN.

Subsequently, the metabolic behavior and sensing performance of NCN toward CYP3A4 was systematically evaluated. In the presence of nicotinamide adenine dinucleotide phosphate (NADPH), NCN could be rapidly metabolized by CYP3A4 to form a single oxidative product, which was fully identified as *N*-cyclopropylmethyl-4-hydroxy-1,8-naphthalimide (HNCN) *via* comparing the retention time and MS/MS spectra with the synthetic standard (Scheme S2 and Fig. S33–S37†). Under physiological conditions, NCN exhibited an absorption peak at 450 nm and negligible background fluorescence (Fig. 1D and E). In contrast, brightly green signals around 555 nm were captured following co-incubation with CYP3A4 and NCN in the presence of NADPH for 30 min, generating a 125-fold fluorescence enhancement. The intramolecular charge transfer properties of NCN and HNCN were analyzed using density functional theory (DFT) calculations. Compared to NCN, HNCN displayed a more obvious electrostatic potential (ESP) and a narrow energy gap between the lowest unoccupied molecular orbital (LUMO) and highest occupied molecular orbital (HOMO), thereby inducing a long wavelength shift of the emission (Fig. 2). Furthermore, CYP3A4-catalyzed NCN 4-hydroxylation exhibited a linear response towards CYP3A4 in both time- (up to 30 min) and CYP3A4 concentration-dependent manner (within the range of 0–7 nM), achieving a detection limit as low as 0.03 nM (Fig. 1F–H). These results demonstrate that NCN exhibits superior sensing performance and ultrahigh signal-to-noise ratio (SNR) for sensing CYP3A4.

Next, the isozyme specificity of NCN towards CYP3A4 was investigated using both P450 reaction phenotyping assays and chemical inhibition assays. P450 reaction phenotyping assays demonstrated that only CYP3A4 could effectively trigger discernible fluorescence enhancement (Fig. 1I), while other human P450 enzymes, including CYP3A5, hardly catalyzed NCN 4-hydroxylation under the same conditions. Chemical inhibition assays further corroborated this selectivity. The broad-spectrum CYP inhibitor 1-aminobenzotriazole (ABT), the potent CYP3A inhibitor ketoconazole (KCZ), and the specific CYP3A4 inhibitor CYP3cide were all capable of completely blocking the NCN 4-hydroxylation in HLMs (Fig. 1J). Furthermore, this enzymatic reaction also displayed robust resistance to interference from various endogenous species, including amino acids, fatty acids, and metal ions (Fig. S38†). The effects of pH on the fluorescence response of HNCN were also investigated, the fluorescence signals of HNCN were stable within the pH range of 7.0–11.0 (Fig. S39†). These findings suggest that NCN is an ideal CYP3A4-activatable fluorescent substrate, showing rapid response, high sensitivity, and robust anti-interference ability, which encouraged us to explore the sensing mechanism of NCN towards CYP3A4.

### Kinetic behavior of CYP3A4-catalyzed NCN 4-hydroxylation

**Fig. 3:**
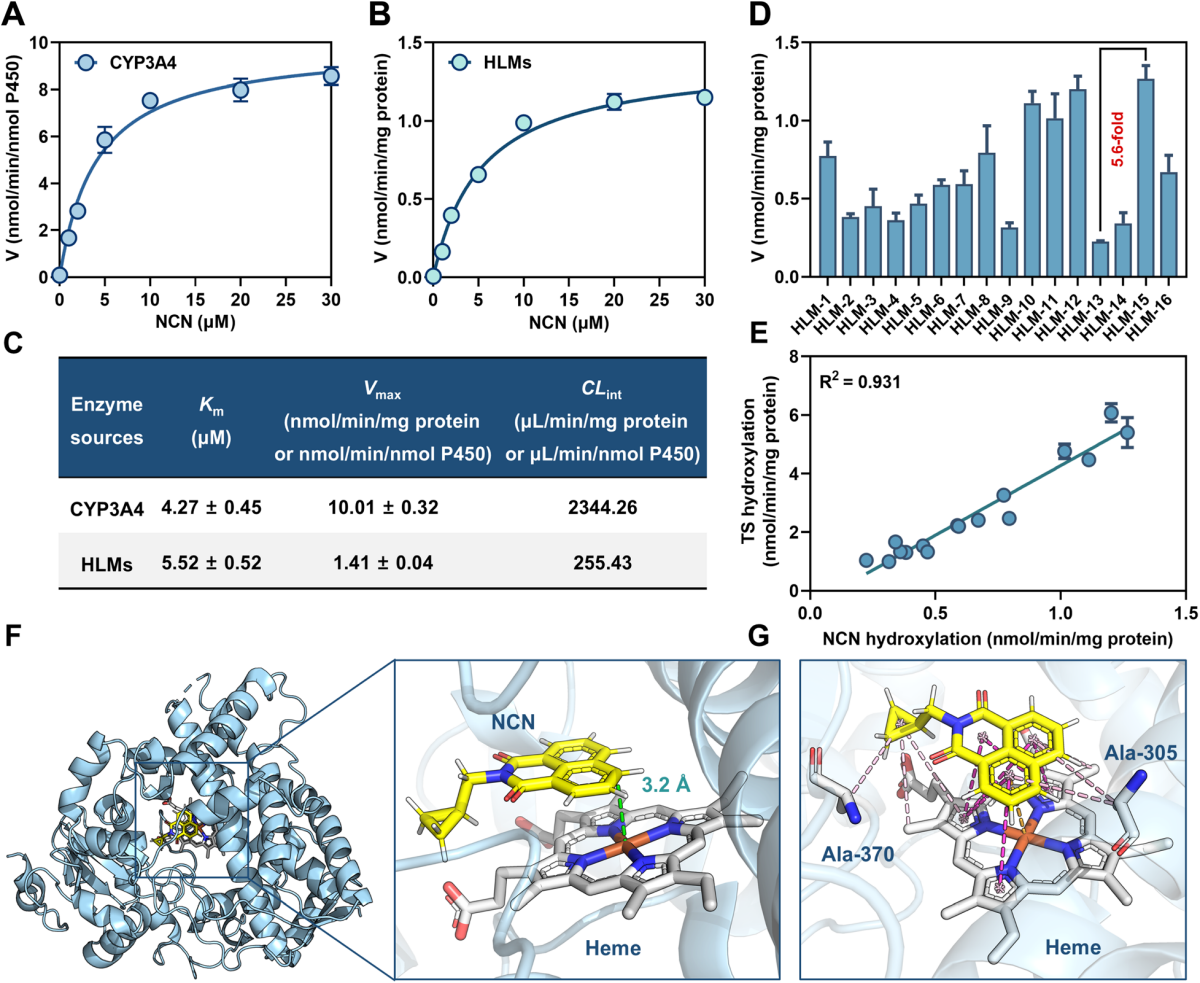
Michaelis–Menten kinetics of NCN 4-hydroxylation catalyzed by CYP3A4 (A) and HLMs (B). (C) Kinetic parameters of NCN 4-hydroxylation in both CYP3A4 and HLMs. (D) The CYP3A4 activity levels in sixteen individual HLM samples using NCN as the probe substrate. (E) Correlation analysis between NCN 4-hydroxylation rates and testosterone 6β-hydroxylation rates in sixteen individual HLM samples. (F) Catalytic distance between the metabolic site of NCN (C-4 site) and the heme iron of CYP3A4 (PDB ID: 5VC0). (G) Binding pose of NCN in CYP3A4.

It is well-known that the enzymatic kinetic behavior of enzyme-activatable fluorescent probes is very important for quantifying target enzyme(s).^41,49^ In this study, NCN 4-hydroxylation kinetics in both CYP3A4 and HLMs were studied by performing a set of kinetic assays. As shown in Fig. 3A and B, NCN 4-hydroxylation in both CYP3A4 and HLMs followed canonical Michaelis–Menten kinetics, as evidenced by the corresponding linear Eadie–Hofstee plots (Fig. S40†). Meanwhile, the *K*_m_ value of NCN 4-hydroxylation in CYP3A4 (*K*_m_ = 4.27 ± 0.45 μM) was very close to that in HLMs (*K*_m_ = 5.52 ± 0.52 μM), underscoring the predominant role of CYP3A4 for catalyzing NCN 4-hydroxylation in HLMs (Fig. 3C). Next, the CYP3A4 activity levels in 16 individual HLM samples were quantitatively analyzed using NCN and testosterone (a common physiological substrate for CYP3A4) as the probe substrate. Striking individual variation (5.6 fold) in NCN 4-hydroxylation formation rate was observed among individuals, consistent with the previously reported variability of CYP3A4 activity in HLMs (Fig. 3D). Notably, a significant linear correlation (*R*^2^ = 0.931, *P* < 0.0001) between NCN 4-hydroxylation rates and testosterone 6β-hydroxylation rates was observed (Fig. 3E). These findings validate that NCN is a specific and reliable substrate for precisely assessing CYP3A4 activity levels in complex biological specimens.

Meanwhile, docking simulations were performed to further explore the recognition mechanisms and binding modes of NCN in the catalytic cavity of CYP3A4. NCN could be well-docked into the catalytic cavity of CYP3A4. The catalytic distance between the C-4 site of NCN and the heme iron was as low as 3.2 Å (Fig. 3F), such a favorable spatial orientation facilitated the oxidative metabolism of NCN in CYP3A4. Meanwhile, the binding poses of NCN were also examined in several other important P450 isozymes, including CYP1A2, CYP2A6, CYP2B6, CYP2C8, CYP2C9, CYP2C19, CYP2D6, and CYP2E1. The spatial distances between the metabolic site of NCN and the heme Fe of each P450 enzyme were greater than that of CYP3A4 (Fig. S41†), which explained the excellent isoenzyme-selectivity of NCN. 2D-interaction analysis indicated that NCN could generate strong hydrophobic interactions (*e.g.*, π–π stacked, π–cation, π–alkyl, and alkyl interactions) with the heme of CYP3A4, suggesting that hydrophobic interactions emerged as the primary driving force for the stable binding of NCN with CYP3A4. Notably, the cyclopropyl moiety on the northern part of NCN formed π–alkyl interactions with Ala-305 and alkyl interactions with Ala-370, respectively (Fig. 3G and S42†), which partly explained the high binding-affinity of NCN when compared to NEN (Fig. S43†). These findings suggest that introducing a cyclopropyl moiety on the northern part of NCN strongly facilitates the recognition and binding of NCN in the catalytic cavity of CYP3A4.

### NCN excelled in functional imaging of CYP3A4 activity in living systems

**Fig. 4:**
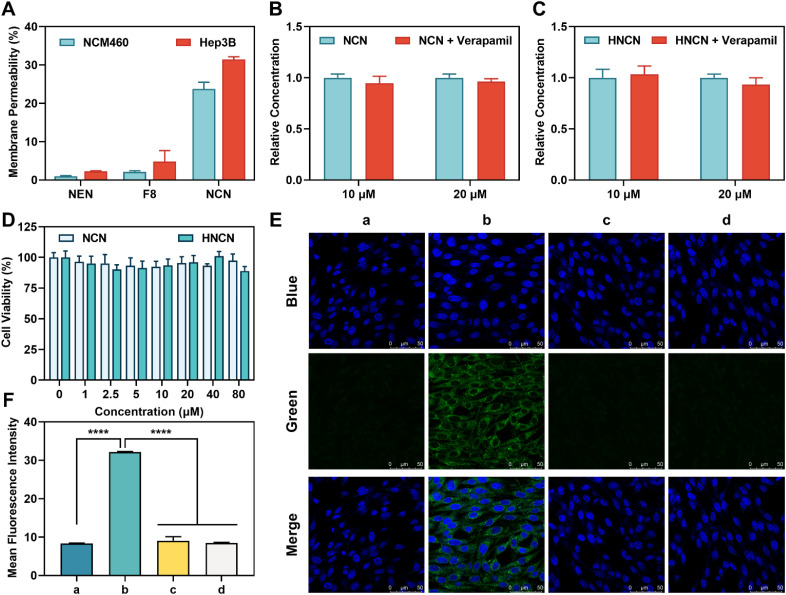
Functional sensing and imaging of CYP3A4 in living hepatocytes. (A) Cell-membrane permeability assays of NEN, F8, and NCN. (B and C) Differences in intracellular uptake of NCN (B) and HNCN (C) in MDR1-MDCK cells in the presence or without verapamil. (D) Cytotoxicity assays of NCN and HNCN in Hep3B cells. (E) Fluorescence imaging of CYP3A4 in living Hep3B cells. Scale bar: 50 μm. (a) Hep3B cells only. (b) Hep3B cells incubated with NCN (20 μM). (c) Hep3B cells were incubated with KCZ (a potent CYP3A4 inhibitor, 10 μM) and NCN. (d) Hep3B cells were incubated with ritonavir (a potent CYP3A4 inhibitor, 10 μM) and NCN. (F) Quantitative analysis of (E). ****P < 0.0001.

It has been reported that the cyclopropyl moiety is a good drug-like moiety and an ideal bioisosteric replacement of hydrophobic benzene rings, such modification may bring improved oral bioavailability and reduced drug efflux that are beneficial for functional imaging of cellular target(s) in living systems.^50^ Here, the practicability of NCN in functional imaging of CYP3A4 activity was investigated in living systems. Prior to cell imaging, the cell-membrane permeability of NCN was determined (Fig. 4A). The results indicated that NCN exhibited improved cell-membrane permeability compared to the previously reported CYP3A4 fluorescent sensors including NEN and F8. Specifically, in NCM460 and Hep3B cell lines, NCN exhibited 23.9-fold and 13.7-fold enhancement in cell-membrane permeability compared to NEN. Meanwhile, the P-gp efflux liability of both NCN and its oxidative metabolite HNCN were also investigated utilizing rhodamine 123 as a positive P-gp substrate. Given that the intracellular levels of P-gp substrates would be increased upon addition of a P-gp inhibitor (such as verapamil). As shown in Fig. S1,† the intracellular levels of either NEN or F8 were significantly enhanced in the presence of verapamil. By contrast, the intracellular exposure levels of both NCN and HNCN were unchanged following treatment with verapamil (Fig. 4B and C). These findings clearly demonstrated that both NCN and HNCN are non-substrates of P-gp. Furthermore, HNCN exhibited excellent phase II metabolic stability (*t*_1/2_ = 81.80 min) and was significantly more stable than the positive drug 7-hydroxycoumarin (*t*_1/2_ = 17.24 min), indicating that HNCN could be used for long-term imaging in living systems (Fig. S44†). CCK-8 assays revealed that NCN, HNCN, KCZ, and ritonavir displayed minimal cytotoxicity and excellent biocompatibility in Hep3B cells (Fig. 4D and S45†). Following co-incubation with NCN in Hep3B cells for 60 min (Fig. 4E and F), bright fluorescence signals from Hep3B cells were observed. In sharp contrast, the CYP3A4 inhibitors (either KCZ or ritonavir) could significantly diminish the fluorescence signals, suggesting that the formation of the fluorogenic metabolite HNCN was CYP3A4-dependent. These observations indicate that NCN can act as a reliable visualization tool for light-up sensing of CYP3A4 in live hepatocytes, highlighting its application for both functional imaging and rapid screening of CYP3A4 modulators in live hepatocytes.

### NCN shows favorable bio-safety profiles in mice

**Fig. 5:**
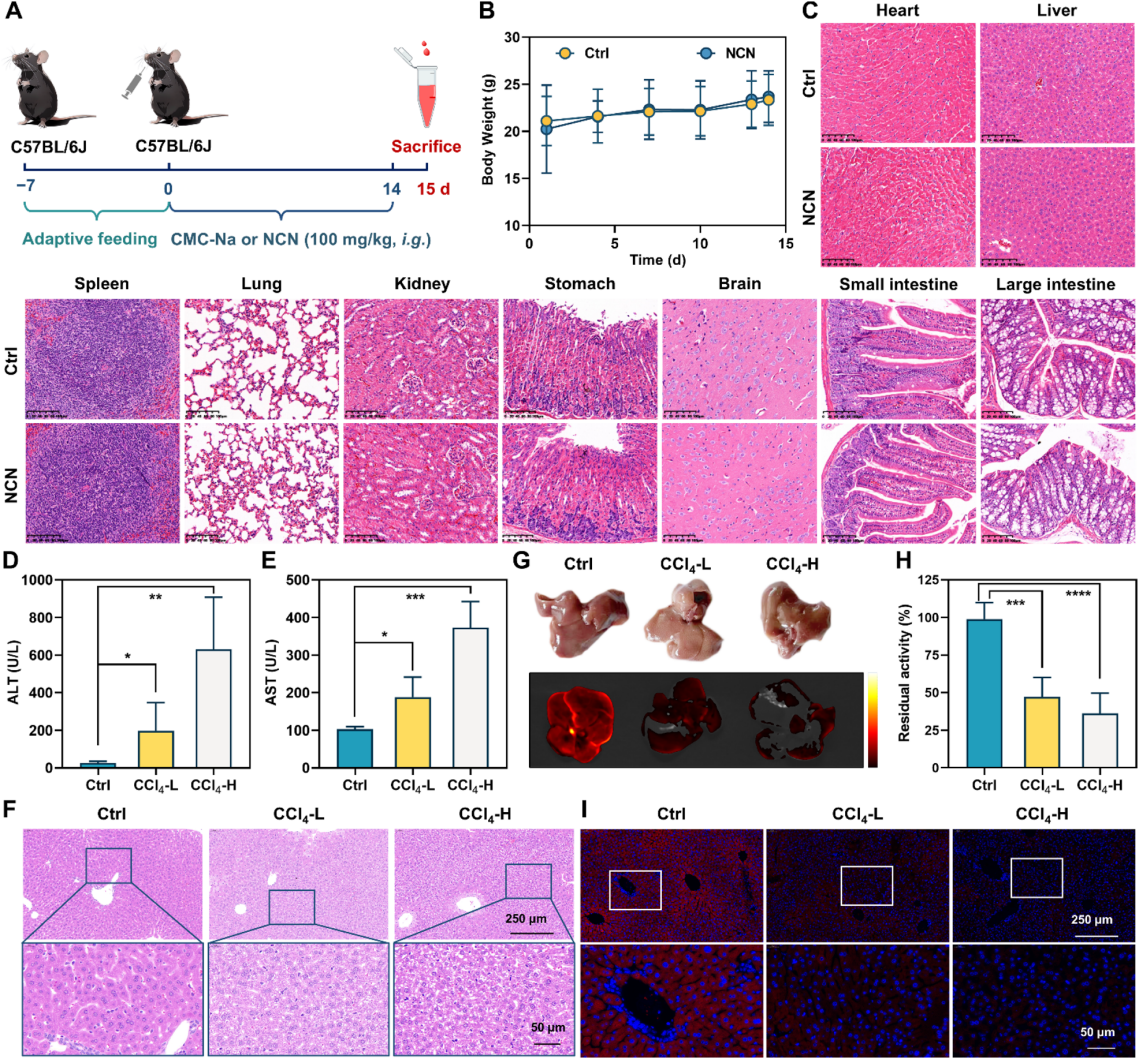
Evaluation of liver metabolic function using NCN as the substrate. (A) Experimental design of tolerance test for NCN. (B) Changes in body weight of mice after oral administration of 100 mg kg^−1^NCN (*n* = 8). (C) Histopathological section of several important organs after oral administration of NCN for 14 consecutive days. Scale bar = 100 μm. (D and E) Serum ALT (D) and AST (E) levels for ctrl group and CCl_4_-induced liver injury group. (F) H&E staining analysis for the ctrl and liver injury groups. (G) Fluorescence imaging of isolated liver of various groups incubated with NCN. (H) Differences in metabolic function between normal liver and injured liver using NCN as the substrate. (I) Immunofluorescence staining of CYP3A4 in various liver tissues. *P < 0.05, **P < 0.01, and ***P < 0.001, ****P < 0.0001 *versus* control.

The above-mentioned findings encouraged us to investigate the practicability of NCN as an *in vivo* CYP3A probe substrate. Prior to *in vivo* tests, a tolerability study on NCN was conducted in mice to decipher its bio-safety profile (Fig. 5A). After oral administration of NCN (100 mg kg^−1^) for 14 consecutive days, no toxicity, mortality, or morbidity signs were observed. Compared to the control group, there were similar weight gain trends in the NCN-treated group, suggesting that NCN did not adversely affect the growth and development of the mice (Fig. 5B). Biochemical tests revealed that the serum levels of liver function indicators (*e.g.*, alanine aminotransferase and aspartate aminotransferase), kidney function indicators (*e.g.*, urea and creatinine), and other indicators (*e.g.*, Na^+^ and K^+^) in the NCN-treated group were all within normal ranges (Fig. S46†). The visceral organs collected from NCN-treated mice were all normal in color, size, and shape, with no evident abnormalities or observed lesions (Fig. S47†). Histopathological examination was conducted to assess potential toxic effects on vital organs from both NCN-treated mice and control mice, including the heart, liver, lungs, spleen, kidneys, brain, and gastrointestinal tract. The results revealed no evidence of lesions or inflammation in these key organs following NCN treatment (Fig. 5C). These observations clearly demonstrate that NCN possesses favorable bio-safety profiles without any evident toxicity or observable organ injury.

### Sensing the liver reserve function using NCN as an imaging tool

As mentioned above, CYP3A4 activity can serve as a reliable functional biomarker for assessing hepatic reserve function. Here, to further investigate the potential of NCN for liver function assessment, a CCl_4_-induced acute liver injury model was established, and the functional integrity of CYP3A4 was evaluated using NCN as an imaging tool. Compared to the healthy mice, the serum levels of ALT and AST significantly elevated in the CCl_4_-challenged mice, indicating substantial hepatocytes damage (Fig. 5D and E). Morphologically, the CCl_4_-injured livers exhibited visible milky white granular substances on the surface and reduced elasticity. Histopathological analysis found the preserved structural integrity in control livers, with hepatocytes exhibiting uniform morphology and no signs of inflammation or necrosis (Fig. 5F). In sharp contrast, the CCl_4_-challenged livers exhibited extensive areas of necrosis and marked inflammatory cell infiltration. Following co-incubation of the liver with NCN, the fluorescence signals around 555 nm were significantly attenuated in the CCl_4_-injured livers (Fig. 5G). Meanwhile, a substantial reduction in NCN 4-hydroxylation rate was observed in S9 fractions of the CCl_4_-injured livers compared to the control group (Fig. 5H). Consistently, both western blot and immunofluorescence staining indicated that the expression levels of CYP3A4 in the CCl_4_-injured livers were significantly downregulated compared to control group (Fig. 5I and S48†). These data highlight the utility of NCN as a reliable visualization tool for assessing liver reserve function or liver injury severity.

### Precise assessing the residual CYP3A4 activity levels in various biological systems

**Fig. 6:**
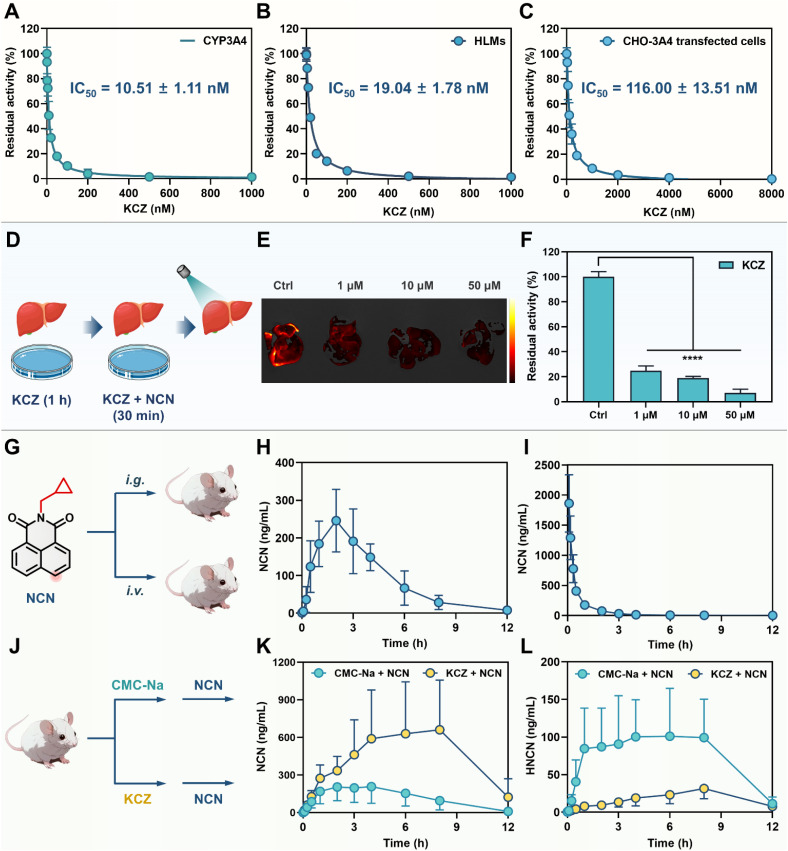
Multidimensional characterization of the inhibitory effects of the CYP3A4 inhibitor KCZ. (A and B) Dose-inhibition curves of KCZ against CYP3A4 in recombinant human CYP3A4 (A) and HLMs (B). (C) Dose-inhibition curves of KCZ against intracellular CYP3A4 (CHO-3A4 stably transfected cells). (D) Experimental procedure for liver organ imaging in the presence of KCZ. (E) Fluorescence imaging of the isolated liver incubated with increasing concentrations of KCZ (0 μM, 1 μM, 10 μM, 50 μM) and NCN (50 μM). (F) Residual activity of hepatic CYP3A4 following KCZ treatment. ****P < 0.0001 *versus* control. (G) Experimental design for testing oral bioavailability of NCN. (H–I) Mean plasma concentration–time curves of NCN (20 mg kg^−1^) following oral administration (H, *n* = 5) and intravenous administration (I, *n* = 5). (J) Experimental procedure for *in vivo* inhibitory effect of KCZ. (K) The mean plasma concentration–time curves of NCN (20 mg kg^−1^, i.g.) in the control group (CMC-Na, *n* = 6) and experimental group (50 mg kg^−1^ of KCZ, i.g., *n* = 6). (L) The mean plasma concentration–time curves of HNCN in the control group (CMC-Na, *n* = 6) and experimental group (50 mg kg^−1^ of KCZ, i.g., *n* = 6) when NCN was administered orally (20 mg kg^−1^, i.g.).

It is well-known that potent CYP3A4 inhibitors may strongly prolong the metabolic half-life and enhance the systematic exposure levels of CYP3A4 substrate drugs. Precise assessment of the pharmacological effects of CYP3A4 inhibitors in living systems is pivotal for delineating their pharmacodynamic properties and translational risks. In this study, KCZ was selected as a model compound for validating the utility of NCN for assessing the pharmacological effects of CYP3A4 inhibitor(s). As shown in Fig. 6A and B, KCZ dose-dependently inhibited CYP3A4 in both recombinant CYP3A4 and HLMs, with IC_50_ values of 10.51 nM and 19.04 nM, respectively. The IC_50_ value of KCZ against CYP3A in mouse liver microsomes (MLMs) was also determined to be 11.18 nM (Fig. S49†). In CYP3A4 stably transfected CHO-3A4 cells and Hep3B cells, the IC_50_ values of KCZ exponentially increased, reaching 116.0 nM and 255.40 nM, respectively (Fig. 6C and S50†). At the organ level (liver), KCZ could also dose-dependently attenuate the formation of the fluorescent product (HNCN), showing the residual activity of ∼20% at a moderate KCZ dose (1 μM) (Fig. 6D–F). It was worth noting that NCN could be absorbed into the circulation system after oral administration, with an oral bioavailability of 12.92% (Fig. 6G–I and Table S5†). This result indicated that NCN could also be used as an *in vivo* probe for assessing the inhibitory effects of CYP3A4 inhibitor(s) in rodents (Fig. 6J–L). Compared to the control group, the pharmacokinetic behavior of NCN (intragastric administration) were significantly modulated by KCZ (50 mg kg^−1^). The plasma exposure (AUC_0-inf_) was increased from 1518 ng mL^−1^ h to 5839 ng mL^−1^ h (3.85-fold). Peak concentration (*C*_max_) increased from 207 ng mL^−1^ to 674 ng mL^−1^ (3.26-fold). The metabolic half-life (*t*_1/2_) was markedly extended from 1.47 h to 2.30 h. Notably, KCZ significantly reduced the circulating exposure of HNCN by 3.92-fold (AUC_(0-inf)_ ranged from 961 ng mL^−1^ h to 245 ng mL^−1^ h) compared with the control group, while the *C*_max_ value of HNCN was decreased 3.16-fold (from 101 ng mL^−1^ to 32 ng mL^−1^). Moreover, the metabolic half-life (*t*_1/2_) of HNCN was significantly prolonged from 1.77 h to 2.88 h. It was also found that the *in vivo* inhibitory effect of KCZ on NCN 4-hydroxylation decreased significantly after 8 h, which was consistent with the metabolic half-life (*t*_1/2_) of KCZ and also suggested that KCZ could be taken orally every 8 h to achieve better *in vivo* effects.^51–53^

Compared to the previous reported probes (NEN and F8), NCN displayed rapid response, appropriate binding-affinity, and ultrahigh sensitivity (Table S6†). More importantly, the high cell-membrane permeability and non-P-gp substrate nature make NCN an ideal tool for sensing cellular CYP3A4 in living systems. Unlike NEN and F8, NCN exhibited favorable safety profiles and an acceptable oral bioavailability, making it a practical *in vivo* probe for CYP3A4. In brief, NCN worked well for the precise assessment of the inhibitory effects of CYP3A4 inhibitor(s) across multidimensional biological systems, including recombinant enzymes, liver preparations, live cells, organs, and the whole body.

## Conclusions

In summary, an integrated strategy was adapted for the rational engineering of an isoform-specific and orally active CYP3A4-activatable fluorogenic substrate suitable for sensing CYP3A4 activity levels in various biological systems, *via* integrating computer-aided substrate design, drug-likeness filtering, and biochemical validation. Following screening of a variety of NEN derivatives, *N*-cyclopropylmethyl-1,8-naphthalimide (NCN) was identified as an optimized fluorogenic substrate for CYP3A4, as such this substrate exhibited exceptional isoform-specificity, rapid response, high SNR, improved cell-membrane permeability, negligible P-gp efflux liability and high bio-safety profiles. NCN was subsequently used for sensing and imaging the CYP3A4 activity levels in various biological systems, including liver preparations, hepatocytes, and the liver organ under both normal and liver injury conditions. Notably, as a cell-permeable agent and a P-gp non-substrate, NCN excelled for *in situ* imaging of CYP3A4 activities in living systems (such as hepatocytes and live organs) and could be absorbed into the circulating system after oral administration. These features ensure that this newly constructed CYP3A4-activatable fluorogenic substrate can sense the hepatic reserve function and the residual CYP3A4 activity levels across multidimensional biological systems, including hepatocytes, the liver, and the whole body. Collectively, an integrated molecular engineering strategy was developed for constructing a highly specific and orally active CYP3A4-activatable fluorogenic sensor for CYP3A4, offering a practical and reliable fluorogenic tool for *in situ* imaging and inhibitor assessment in various biological systems.

## Ethical statement

C57BL/6J male mice (aged 6 weeks, 18–20 g) were purchased from Shanghai SLAC Laboratory Animal Co. Ltd and housed in the Experiment Animal Center of Shanghai University of Traditional Chinese Medicine. This study was approved by the Animal Ethics Committee of Shanghai University of Traditional Chinese Medicine (license No. PZSHUTCM2301050004). Male SD rats (180–200 g) were purchased from Shanghai Laboratory Animal Center and housed in the Experiment Animal Center of Shanghai Institute of Food and Drug Control (license No. SIFDC-IACUC25026).

## Data availability

The data supporting this article have been included as part of the ESI.†

## Author contributions

Feng Zhang: writing – original draft, methodology, investigation, formal analysis, data curation, conceptualization. Yufan Fan: writing – original draft, methodology, formal analysis, data curation, conceptualization. Mei Luo: investigation, validation. Jian Huang: data curation. Bei Zhao: methodology, formal analysis. Lin Chen: data curation. Guanghao Zhu: formal analysis. Yuan Xiong: methodology, validation. Hong Lin: methodology. Chuting Xu: methodology, validation. Xiaodi Yang: methodology. Tony D. James: conceptualization, writing – review, and editing. Guangbo Ge: conceptualization, writing – review and editing, supervision, project administration, funding acquisition, formal analysis.

## Conflicts of interest

The authors declare no conflict of interest.

## Supplementary Material

SC-OLF-D5SC01791B-s001
