# Supplementary material for: An optimized CYP3A4-activatable fluorogenic sensor for in situ functional imaging and multi-dimensional inhibitor assessment
Source: Chem Sci. 2025 May 23;16(25):11386–97. doi: 10.1039/d5sc01791b (PMC12101129; doi:10.1039/d5sc01791b)
Supplement: SC-016-D5SC01791B-s001 [file SC-016-D5SC01791B-s001.pdf]

Supplementary Information (SI) for Chemical Science.
This journal is © The Royal Society of Chemistry 2025

Supporting Information

for

An Optimized CYP3A4-Activatable Fluorogenic Sensor for *in-situ* Functional Imaging and Multi-dimensional Inhibitor Assessment

Feng Zhang[a,1], Yufan Fan[a,1], Mei Luo[a], Jian Huang[b], Bei Zhao[a], Lin Chen[a], Guanghao Zhu[a], Yuan Xiong[a], Hong Lin[c], Chuting Xu[a], Xiaodi Yang[c], Tony D. James[d,e,**], Guangbo Ge[a,*]

[a] *State Key Laboratory of Discovery and Utilization of Functional Components in Traditional Chinese Medicine, Shanghai Frontiers Science Center of TCM Chemical Biology, Institute of Interdisciplinary Integrative Medicine Research, Shanghai University of Traditional Chinese Medicine, Shanghai, 201203, China.*

[b] *Pharmacology and Toxicology Division, Shanghai Institute of Food and Drug Control, Shanghai, 201203, China.*

[c] *The Research Center of Chiral Drugs, Innovation Research Institute of Traditional Chinese Medicine, Shanghai University of Traditional Chinese Medicine, Shanghai 201203, China.*

[d] *School of Chemistry and Chemical Engineering, Henan Normal University, Xinxiang, 453007, China.*

[e] *Department of Chemistry, University of Bath, Bath, BA2 7AY, United Kingdom.*

* Corresponding author. Institute of Interdisciplinary Integrative Medicine Research, Shanghai University of Traditional Chinese Medicine, Shanghai, 201203, China.

**Corresponding author. Department of Chemistry, University of Bath, Bath, BA2 7AY, United Kingdom.

E-mail addresses: t.d.james@bath.ac.uk (T.D. James); geguangbo@shutcm.edu.cn (G. Ge)

[1] These authors contributed equally to this work.

This file contains supplementary materials and methods, fifty supplementary figures and six supplementary tables.

Materials and methods

1.1. Chemicals and instruments

NADPH was obtained from Shangke Biological Medicine (Shanghai) Co., Ltd. ABT, furafylline, tranylcypromine, montelukast, sulfaphenazolum, quinidine, clomethiazole were obtained from Sigma-Aldrich (St. Louis, MO, USA). CYP3cide was purchased from MedChemExpress (USA). $MgCl_2$ was supplied by Sinopharm Chemical Reagent (Shanghai, China). Pooled human liver microsomes from 50 donors (HLMs, lot No. X008067) were purchased from Bioreclamation IVT (Baltimore, MD, USA). A panel of human recombinant cytochrome P450 enzymes (such as CYP1A1, CYP1A2, CYP1B1, CYP2A6, CYP2A13, CYP2B6, CYP2C8, CYP2C9, CYP2C18, CYP2C19, CYP2D6, CYP2E1, CYP2J2, CYP3A4, CYP3A5, CYP4A11, CYP4F2, CYP17A1, and CYP46A1) were obtained from Cypex. 16 individual-donor HLM were supplied by Research Institute for Liver Diseases (RILD, Shanghai, China). LC grades of methanol, acetonitrile, and formic acid were ordered from Fisher Scientific Co. (Fair Lawn, NJ, USA), while ultra-purified water was prepared using a Millipore purification system. Ketoconazole, ritonavir, DMEM Medium, and PBS were obtained from Meilun Bio. Tech (Dalian, China). Cell Counting Kit-8 (CCK-8) was supplied by Yeasen Biotechnology (Shanghai) Co., Ltd. Fetal bovine serum (FBS) and trypsin were purchased from Gibco. Hoechst 33342 was supplied by Shanghai Yuanye Biotechnology Co., Ltd (Shanghai, China). 4% Paraformaldehyde fix solution was supplied by Beyotime (Shanghai, China). CCl_4 was purchased from Sinopharm Chemical Reagent Co., Ltd.

1.2. Structure-guided substrate screening

Five crystal structures of CYP3A4 (PDB ID: 3NXU, 4I4G, 4K9U, 5TE8, and 5VC0) were downloaded from the Protein Data Bank (http://www.rcsb.org/). According to a standard protocol, the PDBQT files for five receptors were prepared by deleting water molecules and redundant heteroatoms, adding the polar hydrogens, adding Kollman charges, and assigning AD4 type. All ligands were submitted to AutoDock Tools using default parameters after energy minimization. A grid box with a spacing of 0.375 Å and

dimensions of 80 × 80 × 80 points centered on the active site (heme). AutoDock Vina software conducted molecular docking simulations. PyMOL (Version 2.3, Schrödinger) was used to analyze output poses by quantifying the distance between the C-4 site of naphthalimide and the Fe atom of heme (C−Fe).

1.3 Synthesis of candidate probes

A series of *N*-substituted naphthalimide derivatives were synthesized according to the following steps. 1,8-Naphthalic anhydride (2.0 mmol) and each amine compounds (2.0 mmol) were dissolved and refluxed in absolute EtOH (10 mL) for 8 h. After cooling, the precipitate was filtered and washed with cold EtOH to afford the crude product, which was further purified by a silica gel column.

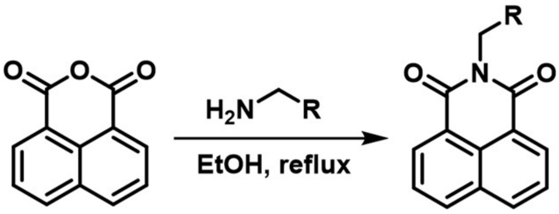

Scheme S1. Synthetic procedure of candidate probes.

Probe 5 (**NCN**): white powder (464 mg, yield = 92.4%). ^{1}H NMR (600 MHz, Chloroform-*d*) *δ* 8.63 (d, *J* = 7.3 Hz, 2H), 8.23 (d, *J* = 8.2 Hz, 2H), 7.78 (t, *J* = 7.7 Hz, 2H), 4.11 (d, *J* = 7.2 Hz, 2H), 1.39 (hept, *J* = 6.8 Hz, 1H), 0.52 (d, *J* = 6.5 Hz, 4H). ^{13}C NMR (151 MHz, Chloroform-*d*) *δ* 164.47, 133.84, 131.61, 131.20, 128.26, 126.92, 122.85, 44.69, 10.28, 3.94. ESI-MS: M = 251.26; found *m/z* 252.09 $[M+H]^+$.

Probe 6: white powder (454 mg, yield = 85.6%). ^{1}H NMR (600 MHz, Chloroform-*d*) *δ* 8.62 (dd, *J* = 7.2, 1.2 Hz, 2H), 8.24 – 8.21 (m, 2H), 7.77 (t, *J* = 7.7 Hz, 2H), 4.29 (d, *J* = 7.3 Hz, 2H), 2.86 (hept, *J* = 7.6 Hz, 1H), 2.05 (qq, *J* = 7.2, 4.6, 4.0 Hz, 2H), 1.91 (dddd, *J* = 27.8, 25.3, 13.1, 8.0 Hz, 4H). ^{13}C NMR (151 MHz, Chloroform-*d*) *δ* 164.48, 133.81, 131.58, 131.22, 128.20, 126.92, 122.78, 45.03, 34.72, 26.45, 18.35. ESI-MS: M = 265.11; found *m/z* 266.11 $[M+H]^+$.

Probe 8: white powder (475 mg, yield = 84.4%). ^{1}H NMR (600 MHz, Chloroform-*d*) *δ* 8.63 (d, *J* = 7.3 Hz, 2H), 8.24 (d, *J* = 8.2 Hz, 2H), 7.79 (t, *J* = 7.7 Hz, 2H), 4.35 (dd, *J*

= 13.0, 7.6 Hz, 1H), 4.22 (dd, *J* = 13.0, 7.1 Hz, 1H), 4.00 (td, *J* = 8.1, 5.6 Hz, 1H), 3.89 (dd, *J* = 8.6, 6.9 Hz, 1H), 3.81 (q, *J* = 7.6 Hz, 1H), 3.70 (dd, *J* = 8.6, 6.1 Hz, 1H), 2.86 (hept, *J* = 7.0 Hz, 1H), 2.04 (dtd, *J* = 13.1, 7.7, 5.6 Hz, 1H), 1.84 (ddd, *J* = 14.3, 12.7, 7.0 Hz, 1H). ^{13}C NMR (151 MHz, Chloroform-*d*) *δ* 164.51, 134.07, 131.61, 131.42, 128.21, 127.00, 122.53, 71.49, 67.73, 42.24, 38.78, 30.24. ESI-MS: M = 281.11; found *m/z* 282.10 [M+H]$^+$.

Probe 10: white crystal (408 mg, yield = 69.3%). ^{1}H NMR (600 MHz, DMSO-*d*$_6$) *δ* 8.51 (dd, *J* = 7.2, 1.2 Hz, 2H), 8.47 (dd, *J* = 8.3, 1.2 Hz, 2H), 7.88 (dd, *J* = 8.2, 7.2 Hz, 2H), 4.19 (t, *J* = 7.2 Hz, 2H), 2.68 (t, *J* = 7.2 Hz, 2H), 2.54 – 2.52 (m, 4H), 1.67 (p, *J* = 2.9 Hz, 4H). ^{13}C NMR (151 MHz, DMSO-*d*$_6$) *δ* 163.83, 134.83, 131.79, 131.25, 127.84, 127.73, 122.51, 54.22, 53.53, 39.16, 23.68. ESI-MS: M = 294.14; found *m/z* 295.14 [M+H]$^+$.

Probe 11: pinkish-white crystal (414 mg, yield = 66.8%). ^{1}H NMR (600 MHz, Chloroform-*d*) *δ* 8.62 (d, *J* = 7.3 Hz, 2H), 8.24 (d, *J* = 8.2 Hz, 2H), 7.82 – 7.77 (m, 2H), 4.39 (t, *J* = 6.9 Hz, 2H), 3.73 (t, *J* = 4.6 Hz, 4H), 2.77 (t, *J* = 6.9 Hz, 2H), 2.73 – 2.58 (m, 4H). ^{13}C NMR (151 MHz, Chloroform-*d*) *δ* 164.25, 134.02, 131.64, 131.27, 128.24, 126.98, 122.66, 56.13, 53.75. ESI-MS: M =310.13; found *m/z* 311.13 [M+H]$^+$.

Probe 12: yellowish-white crystal (452 mg, yield = 73.1%). ^{1}H NMR (600 MHz, Chloroform-*d*) *δ* 8.61 (dd, *J* = 7.2, 1.1 Hz, 2H), 8.23 (dd, *J* = 8.2, 1.1 Hz, 2H), 7.77 (dd, *J* = 8.2, 7.2 Hz, 2H), 4.37 (dd, *J* = 7.8, 6.5 Hz, 2H), 2.91 (t, *J* = 4.8 Hz, 4H), 2.73 – 2.70 (m, 2H), 2.62 (s, 4H), 2.08 (s, 1H). ^{13}C NMR (151 MHz, Chloroform-*d*) δ 164.20, 133.94, 131.61, 131.22, 128.20, 126.95, 122.68, 56.25, 54.48, 46.00, 37.34. ESI-MS: M = 309.15; found *m/z* 310.15 [M+H]$^+$.

Probe 13: white crystal (428 mg, yield = 66.2%). ^{1}H NMR (600 MHz, Chloroform-*d*) *δ* 8.61 (d, *J* = 7.2 Hz, 2H), 8.23 (d, *J* = 8.1 Hz, 2H), 7.77 (t, *J* = 7.7 Hz, 2H), 4.36 (t, *J* = 7.2 Hz, 2H), 2.78 – 2.43 (m, 10H), 2.32 (s, 3H). ^{13}C NMR (151 MHz, Chloroform-*d*) *δ*

164.19, 133.94, 131.61, 131.22, 128.20, 126.95, 122.68, 55.59, 55.10, 53.05, 45.87, 37.51. ESI-MS: M = 323.16; found *m/z* 324.16 $[M+H]^+$.

Probe 15: faint yellow (450 mg, yield = 99.1%). ^1H NMR (600 MHz, Chloroform-*d*) δ 8.65 (dd, J = 7.3, 1.2 Hz, 2H), 8.23 (dd, J = 8.3, 1.1 Hz, 2H), 7.77 (dd, J = 8.2, 7.3 Hz, 2H), 7.35 (dd, J = 1.9, 0.9 Hz, 1H), 6.49 – 6.46 (m, 1H), 6.33 (dd, J = 3.3, 1.8 Hz, 1H), 5.43 (s, 2H). ^{13}C NMR (151 MHz, Chloroform-*d*) δ 163.89, 150.49, 142.08, 134.13, 131.63, 131.53, 128.23, 126.96, 122.54, 110.37, 109.14, 36.35. ESI-MS: M = 277.07; found *m/z* 278.07 $[M+H]^+$.

Probe 18: white crystal (636 mg, yield = 96.7%). ^1H NMR (600 MHz, Chloroform-*d*) δ 8.65 (dd, J = 7.3, 1.2 Hz, 2H), 8.26 (dd, J = 8.3, 1.1 Hz, 2H), 7.79 (dd, J = 8.2, 7.3 Hz, 2H), 7.73 (s, 1H), 5.47 (s, 2H). ^{13}C NMR (151 MHz, Chloroform-*d*) δ 163.77, 152.62, 141.95, 135.09, 134.57, 131.73, 131.66, 128.20, 127.07, 122.14, 35.61. ESI-MS: M = 328.01; found *m/z* 329.01 $[M+H]^+$.

Probe 22: white powder (708 mg, yield = 89.8%). ^1H NMR (600 MHz, DMSO-d_6) δ 8.51 – 8.43 (m, 4H), 7.91 – 7.85 (m, 2H), 7.75 (t, J = 6.3 Hz, 1H), 7.59 (d, J = 8.2 Hz, 2H), 7.23 (d, J = 8.0 Hz, 2H), 4.12 (t, J = 6.6 Hz, 2H), 3.10 (q, J = 6.5 Hz, 2H), 2.26 (s, 3H). ^{13}C NMR (151 MHz, DMSO-d_6) δ 163.98, 142.91, 138.12, 134.71, 131.75, 131.09, 129.95, 127.95, 127.63, 126.81, 122.65, 21.34. ESI-MS: M = 394.10; found *m/z* 395.09 $[M+H]^+$.

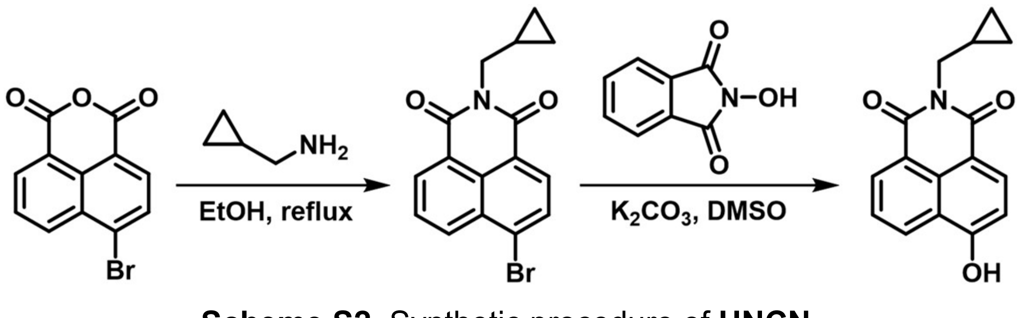

Scheme S2. Synthetic procedure of **HNCN**.

4-Bromo-1,8-naphthalic anhydride (2.0 mmol) and cyclopropylmethanamine (3.0 mmol) were dissolved in 10 mL EtOH and refluxed for 8 h. After cooling, the precipitate was filtered and washed with EtOH to afford the faint yellow intermediate (572 mg, yield = 86.7%) without further purification. Next, the crude intermediate (495 mg, 1.5 mmol) and K_2CO_3 (276 mg, 2.0 mmol) were mixed in DMSO (20 mL) and stirred at 100 °C for 4 h after adding 2-hydroxyisoindoline-1,3-dione (326 mg, 2.0 mmol). Subsequently, the reaction solution was poured into plenty of ice water. After adjusting the pH to 4~5, the generated precipitate was filtered and washed with water. Finally, the crude compound was further purified by a silica gel column chromatograph (petroleum ether: ethyl acetate = 3: 1) to obtain yellow product (365 mg, 91.3%). ^{1}H NMR (600 MHz, DMSO-d_6) δ 11.87 (s, 1H), 8.56 – 8.51 (m, 1H), 8.48 (dd, J = 7.3, 1.6 Hz, 1H), 8.36 (dd, J = 8.2, 1.6 Hz, 1H), 7.78 – 7.74 (m, 1H), 7.16 (dd, J = 8.2, 1.5 Hz, 1H), 3.92 (d, J = 7.1 Hz, 2H), 1.23 (ddt, J = 10.2, 7.2, 3.7 Hz, 1H), 0.46 – 0.41 (m, 2H), 0.39 (dd, J = 4.4, 2.4 Hz, 2H). ^{13}C NMR (151 MHz, DMSO-d_6) δ 164.35, 163.67, 160.76, 134.11, 131.68, 129.68, 129.43, 126.10, 122.85, 122.26, 113.05, 110.47, 44.04, 10.65, 4.10. ESI-MS: M = 267.09; found *m/z* 266.08 $[M-H]^-$.

1.4. Oxidative metabolism of candidate substrates

The incubation system (200 μL) contained PBS (pH 7.4, 100 mM), enzyme sources (HLM), $MgCl_2$, NADPH, and substrate. After pre-incubating at 37 °C for 3 min, the reactions were initiated by adding NADPH. Then, all samples were terminated by adding 200 μL ice-cold acetonitrile and centrifugated at 20,000 ×g and 4 °C for 30 min. After that, the supernatants were taken for further analysis.

1.5. Metabolic profiling of candidate substrates

The oxidative metabolites of each substrate candidate in HLMs were identified utilizing Shimadzu UFLC system coupled with a Triple TOF 5600 mass spectrometer (SCIEX). The mobile phase consisted of 0.1% formic acid in water (A) and acetonitrile (B) with the following elution program: 0-2 min, 2% B; 2-10 min, 2%-90% B; 10-12 min, 90% B; 12-12.5 min, 90%-2% B; 12.5-15.0 min, 2% B. The flow rate was set at 0.4

mL/min, while the column temperature was maintained at 40 ºC. The full-scan mass was acquired over the range from 50-1500 (*m/z*). The ion source gas1 and gas2 were 50 psi, curtain gas was 35 psi, ion spray voltage was set at +5000 kV/-4500 kV, and ion source temperature was 500/450 ºC. The declustering potential (DP) and collision energy (CE) were ± 80 V and ± 45 V, respectively.

1.6. P450 reaction phenotyping and chemical inhibition assays

P450 reaction phenotyping analysis of **NCN** was determined using a panel of human recombinant cytochrome P450 enzymes, while the inhibitory effects of CYP specific inhibitors on **NCN** 4-hydroxylation in HLMs were assessed. The final concentration of each human recombinant CYP enzyme in the incubation system was 5 nM. Briefly, each sample was co-incubated with **NCN** at 37 °C for 30 min, the formation rate of **HNCN** was determined to assess the metabolic specificity of **NCN**.

1.7. DFT calculations

Gaussian program conducted a GMMX conformational analysis using a DFT/B3LYP/6-31G(d) pre-optimized 3D geometry. The resulting GMMX conformers were optimized at B3LYP/6-31G(d) level. Moreover, all conformers were real minima, as no imaginary vibrational frequencies were found. The energy ranking was performed to find the minimum energy conformer. The minima conformer was optimized at B3LYP /6-311G(d,p) level. The molecular orbital energy levels and electrostatic potential (ESP) were calculated at the DFT/B3LYP/6-311G(d,p) level. Frontier orbital and ESP were depicted by Multiwfn (Lu and Chen 2011) and VMD (William Humphrey 1996).

1.8. Enzyme kinetic analysis

The kinetic parameters of **NCN** 4-hydroxylation in HLMs were determined by performing a set of hydroxylation kinetic assays. Kinetic parameters (K_m and V_{max}) for **NCN** 4-hydroxylation were fitted by the following equation (a):

$$V = \frac{V_{max} \times [S]}{K_m + [S]} \#(1)$$

K_m is the Michaelis constant of **NCN** 4-hydroxylation, V_{max} is the maximum velocity of **NCN** 4-hydroxylation, V_{max}/K_m is utilized to calculate the intrinsic clearance (CL_{int}).

1.9. Cell-membrane permeability assay

Hep3B cells were cultured with the DMEM media supplemented with 10% fetal bovine serum (FBS) in a humidified culture chamber at 37 °C with 95% air and 5% CO_2. The cells were trypsinized and resuspended with the DMEM basal medium at a density of $2×10^5$ cells/mL. 200 µL cell suspension was incubated with **NCN** (20 µM) at 37 °C for 1 h. Then, the cells were centrifuged (1000 rpm, 3 min), resuspended in DMEM media, and centrifuged again. Next, the cells were lysed with ice-cold methanol (200 µL) and centrifuged (20000 g, 30 min, 4 °C). The content of **NCN** in the supernatant was determined.

1.10. P-gp transporter assay

To test whether **NCN** is pumped extracellularly by P-gp, MDR1-MDCK cells (a transfected cell line overexpressing human P-glycoprotein) were seeded in 96-well plates supplemented with DMEM media with 10% FBS. Then, verapamil (a potent P-gp inhibitor, 100 µL) was added to 96-well plates and cultured for 15 min. After that, the cells were treated with **NCN**/Rhodamine 123 (100 µL) for 2 h. The cells were collected and washed with PBS, and lysed by 200 µL ice-cold acetonitrile. Next, the cell supernatant was collected by centrifugation at 20,000 g, 4 °C for 30 min. The content of **NCN** in the cells was determined.

1.11. Functional imaging of CYP3A4 in living cells

Hep3B cells were seeded in 35 mm glass bottom dishes with 4 chambers at a density of $1 × 10^5$ cells per chamber. With/without ketoconazole or ritonavir (20 µM, final concentration), Hep3B cells were cultured in DMEM media containing DMSO/**NCN** (20 µM, final concentration, 1 h) and Hoechst 33342 (10 µg/mL, final concentration, 15 min), respectively. Then, the cells were fixated with 4% paraformaldehyde (PFA) and washed with PBS. Functional imaging of CYP3A4 in living cells was performed using confocal microscopy. Blue channel for Hoechst 33342: $λ_{ex}$ 405 nm, $λ_{em}$ 425−475 nm; Green Channel for **HNCN**: $λ_{ex}$ 488 nm, $λ_{em}$ 530−580 nm.

1.12. Animal experiments

C57BL/6J male mice (aged 6 weeks, 18−20 g) were purchased from Shanghai SLAC Laboratory Animal Co. Ltd and housed in the Experiment Animal Center of Shanghai University of Traditional Chinese Medicine. This study was approved by the Animal Ethics Committee of Shanghai University of Traditional Chinese Medicine (license No. PZSHUTCM2301050004). Male SD rats (180−200 g) were purchased from Shanghai Laboratory Animal Center and housed in the Experiment Animal Center of Shanghai Institute of Food and Drug Control (license No. SIFDC-IACUC25026). All animals were subjected to adaptive feeding for 7 d prior to the experiment.

1.13. Western blotting assays

Liver tissues were initially rinsed twice with PBS lysed using RIPA buffer, supplemented with 1% phosphatase inhibitor and 1% protease inhibitor. The protein concentration of lysates was determined by utilizing the Nanodrop One (Thermo Scientific, USA) spectrophotometer. The proteins were separated using 10% sodium dodecyl sulfate-polyacrylamide gel electrophoresis, followed by membrane transfer, sealing, and overnight incubation of the primary antibody at 4 °C. Subsequently, the PVDF membrane was incubated with secondary antibody (titer 1:10,000) for 1 h at room temperature. Protein bands were visualized using Enhanced Chemiluminescence. The relative expression level of the proteins was calculated by using α-Tubulin as an internal reference. Antibodies against CYP3A4 (Proteintech, UK) and α-Tubulin (Proteintech, UK) were used at a dilution of 1:1000 and 1:10,000, respectively. The Image J analysis system was used to analyze chemiluminescent data.

1.14. Imaging of CYP3A activity levels in liver organs

C57BL/6J male mice (aged 6 weeks, 18−20 g) were housed under controlled environmental conditions (25 ± 2 °C, 50 ± 10% humidity; 12 h light/dark cycle) with free access to food and water. All mice were subjected to adaptive feeding for 7 d. Prior to the experiment, all mice were randomly divided into control group, CCl_4 low

dose group (CCl_4-L, 0.15%), and CCl_4 high dose group (CCl_4-H, 0.3%). CCl_4 was dissolved in vegetable oil and administered *via* intraperitoneal injection. After that, mice were fasted and allowed to drink water freely. After 18 h, blood samples were collected to determine the level of ALT and AST, while liver tissues were collected and washed with PBS. Then, the liver tissues were soaked 30 min in a solution containing **NCN** (50 μM, 5 mL) for fluorescent imaging. After organ imaging, the tissues were ground, and centrifuged, and the supernatant was diluted with 10-fold acetonitrile to precipitate the protein. After centrifugation again, the supernatant was determined to quantify the level of **HNCN**.

1.15. Bio-safety assessment of **NCN** in healthy mice

Sixteen C57BL/6J mice (8 males and 8 females, 6 weeks) were randomly assigned into two groups (4 males and 4 females). All mice were subjected to adaptive feeding for 7 d before the experiment. **NCN** was dissolved in 0.5% CMC-Na, and mice were orally administered **NCN** (100 mg/kg)/0.5% CMC-Na continuously for 14 d. The mice were weighed every day, and their status was observed. After 14 d, blood and tissues from all mice were obtained for bio-safety assessment. Serum ALT, AST, TP, UREA, CREA, TG, Na^+, and K^+ levels were tested by using a fully automated serum biochemistry analysis (Thermo, USA). Mice organs were weighed and the organ index was calculated, and hematoxylin and eosin (H&E) stains were provided by Shanghai Ruibaohe Biotechnology Co., Ltd.

1.16. Liver organ imaging in the presence of CYP3A4 inhibitor

C57BL/6J male mice were subjected to adaptive feeding for 7 d. The mice were executed, and all liver tissues were removed and soaked in ketoconazole (0, 1, 10, 50 μM, final concentration, 5 mL) at 37 °C for 1 h. Next, **NCN** (50 μM, final concentration) was added to the solution containing liver tissues and incubated at 37 °C for 30 min. After that, the liver tissues were removed, and fluorescence imaging of the liver was performed using VISQUE Invivo Smart-LF. After organ imaging, the tissues were ground, and centrifuged, and the supernatant was diluted with 10-fold acetonitrile to

precipitate the protein. After centrifugation again, the supernatant was determined to quantify the levels of **HNCN**.

1.17. Oral bioavailability of **NCN**

Twelve SD rats were randomly divided into two groups (oral **NCN** group and intravenous injection **NCN** group). The oral dose of **NCN** is 20 mg/kg, and blood samples were collected at 0 min, 5 min, 15 min, 30 min, 1 h, 2 h, 3 h, 4 h, 6 h, 8 h, 12 h, and 24 h, respectively. **NCN** was injected at a dose of 2 mg/kg and then blood samples were collected at 0 min, 5 min, 10 min, 20 min, 30 min, 1 h, 2 h, 3 h, 4 h, 6 h, 8 h, 12 h, and 24 h, respectively. Next, the **NCN** and **HNCN** in plasma were quantitatively analyzed by precipitating proteins with 5-fold volume of acetonitrile.

1.18. CYP3A4 inhibition assay

The anti-CYP3A4 effects were tested by **NCN**-based biochemical assay. Briefly, a total of 200 μL incubation system PBS (pH 7.4, 100 mM), HLMs, $MgCl_2$, NADPH, **NCN**, and tested inhibitor/solvent, were pre-incubated at 37 °C for 3 min. Upon the addition of NADPH, all samples were incubated in a microplate reader ($\lambda_{ex}/\lambda_{em}$ = 450 nm/555 nm, SpectraMax® iD3, Molecular Devices, Austria) for 30 min at 37 °C. The residual activity (%) of CYP3A4 = (the fluorescence intensity in the presence of inhibitors) / (the fluorescence intensity of the control (solvent only)) × 100%.

1.19. Inhibitory effect of KCZ in CHO-3A4 stably transfected cells

To assess the inhibitory effects of KCZ on CYP3A4 in living cells, CHO-3A4 stably transfected cell line was grown in F12 medium supplemented with 10% fetal bovine serum (FBS), in a humidified atmosphere (95% air and 5% CO_2) at 37 °C. CHO-3A4 stably transfected cell line was seeded in 96-well plates. When the cells in 96-well plates were about 60% confluent, they were treated with KCZ for 1 h. After that, the cells were treated with **NCN** (10 μM, final concentration) for 30 min, then terminated by adding 100 μL ice-cold acetonitrile. The reaction mixture was centrifuged at 20,000 ×g for 30 min. The supernatant was subjected to LC-FD analysis.

1.20. In vivo inhibitory effect of ketoconazole

Twelve rats were randomly divided into two groups (CMC-Na + **NCN** group, ketoconazole + **NCN** group). The oral dose of **NCN** and ketoconazole is 20 mg/kg and 50 mg/kg, respectively. Firstly, blank blood samples were collected. Then, ketoconazole or CMC-Na (control group) was administered orally. After 30 min, **NCN** was administered orally and blood samples were collected at the following time points (5 min, 15 min, 30 min, 1 h, 2 h, 3 h, 4 h, 6 h, 8 h, and 12 h) after administration. The **NCN** and **HNCN** in plasma were quantitatively analyzed by precipitating proteins with 5-fold volume of acetonitrile.

1.21. Statistical analysis

All data are expressed as the mean ± standard deviation. The K_m, V_{max}, and IC_{50} values were determined *via* non-linear regression analysis using GraphPad Prism software. All statistical analyses were carried out using GraphPad Prism software (GraphPad Prism 8.0), and Origin (2025).

Supplementary figures and tables

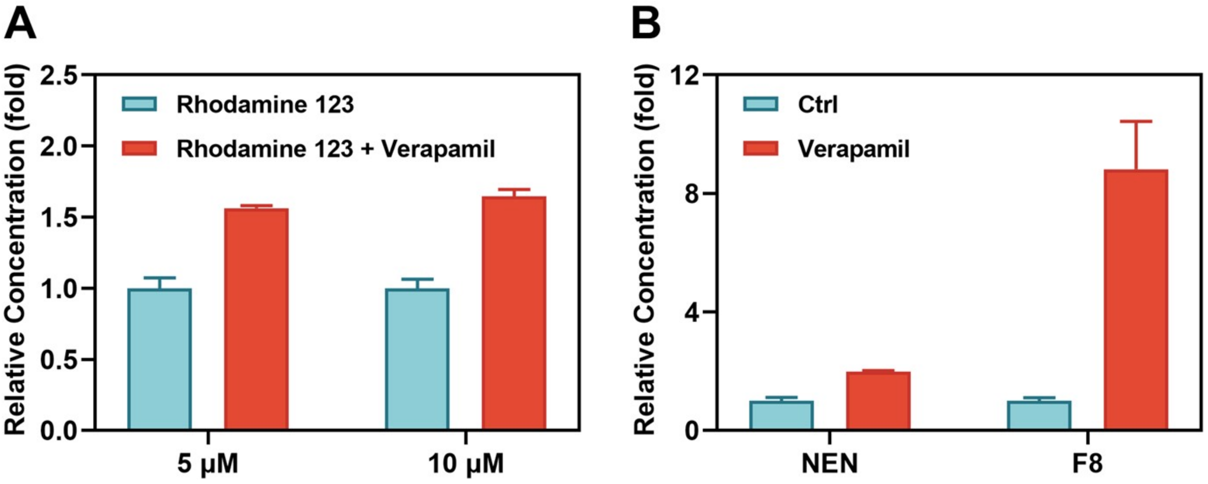

Fig. S1. P-gp efflux transport assays of Rhodamine 123, **NEN**, and **F8**.

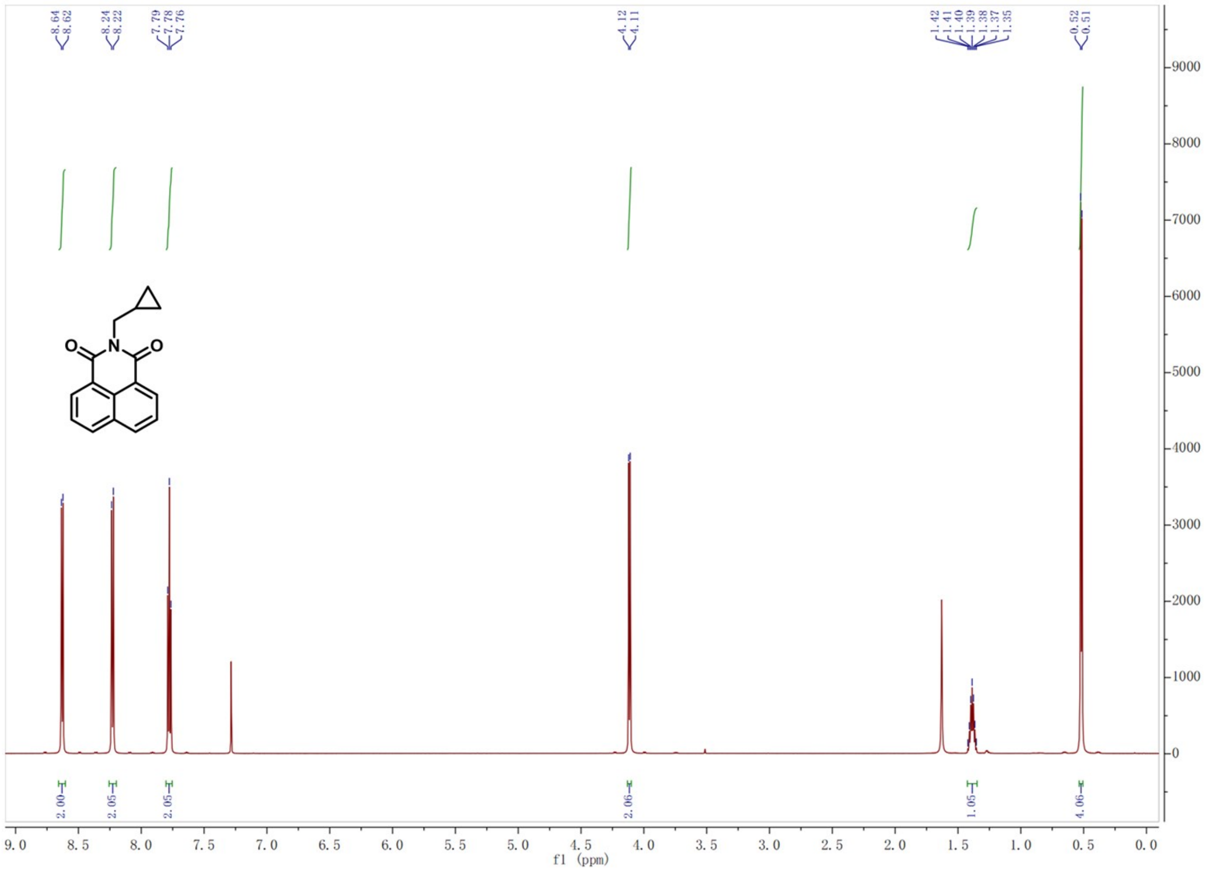

Fig. S2. ^{1}H NMR spectra of probe **5** (**NCN**).

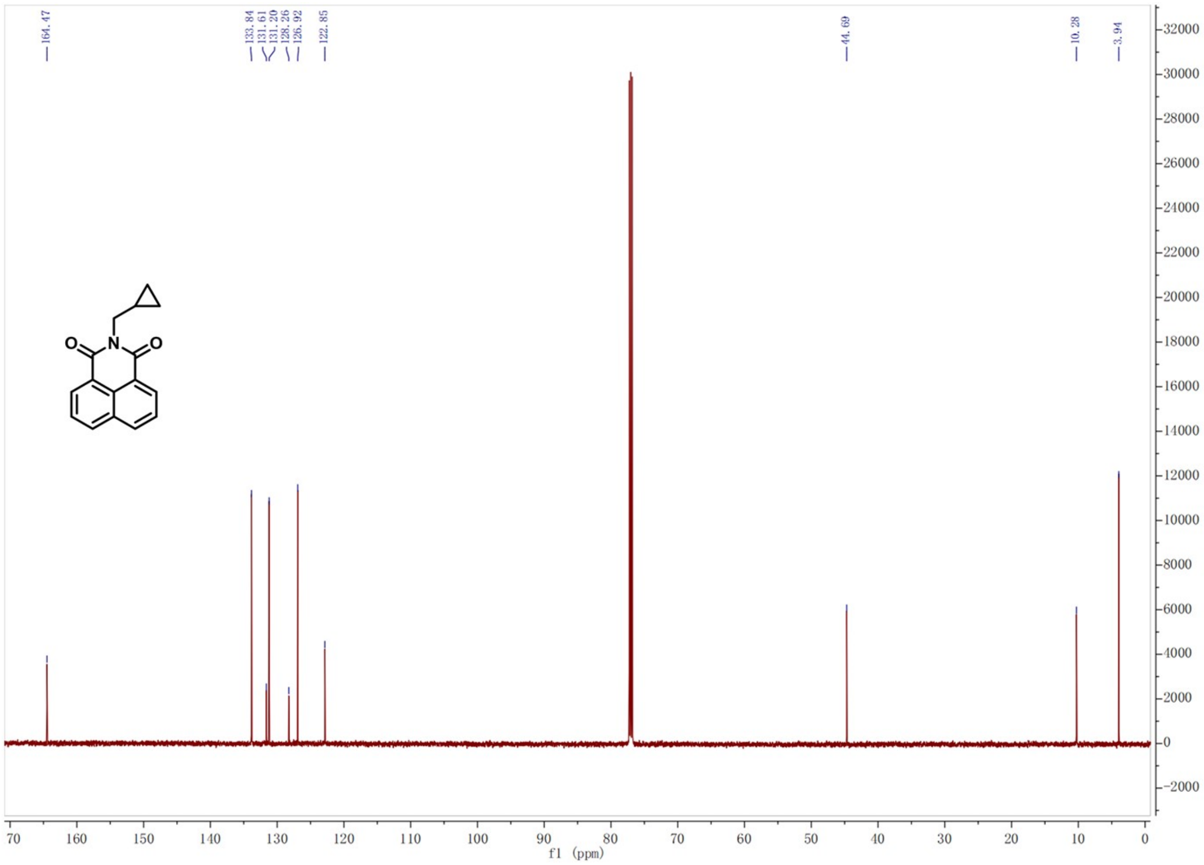

Fig. S3. ^{13}C NMR spectra of probe **5** (**NCN**).

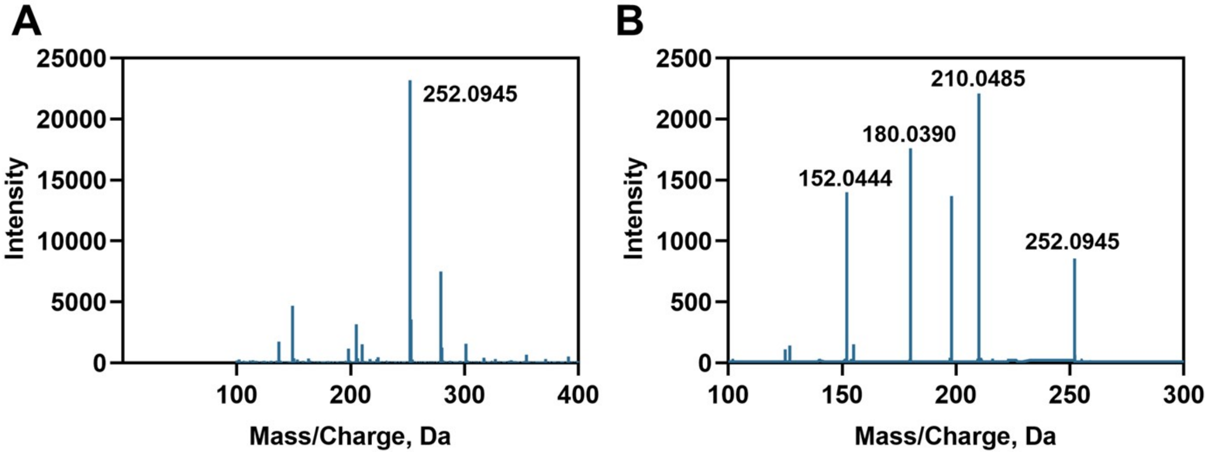

Fig. S4. MS (A) and MS/MS (B) spectra of probe **5** (**NCN**).

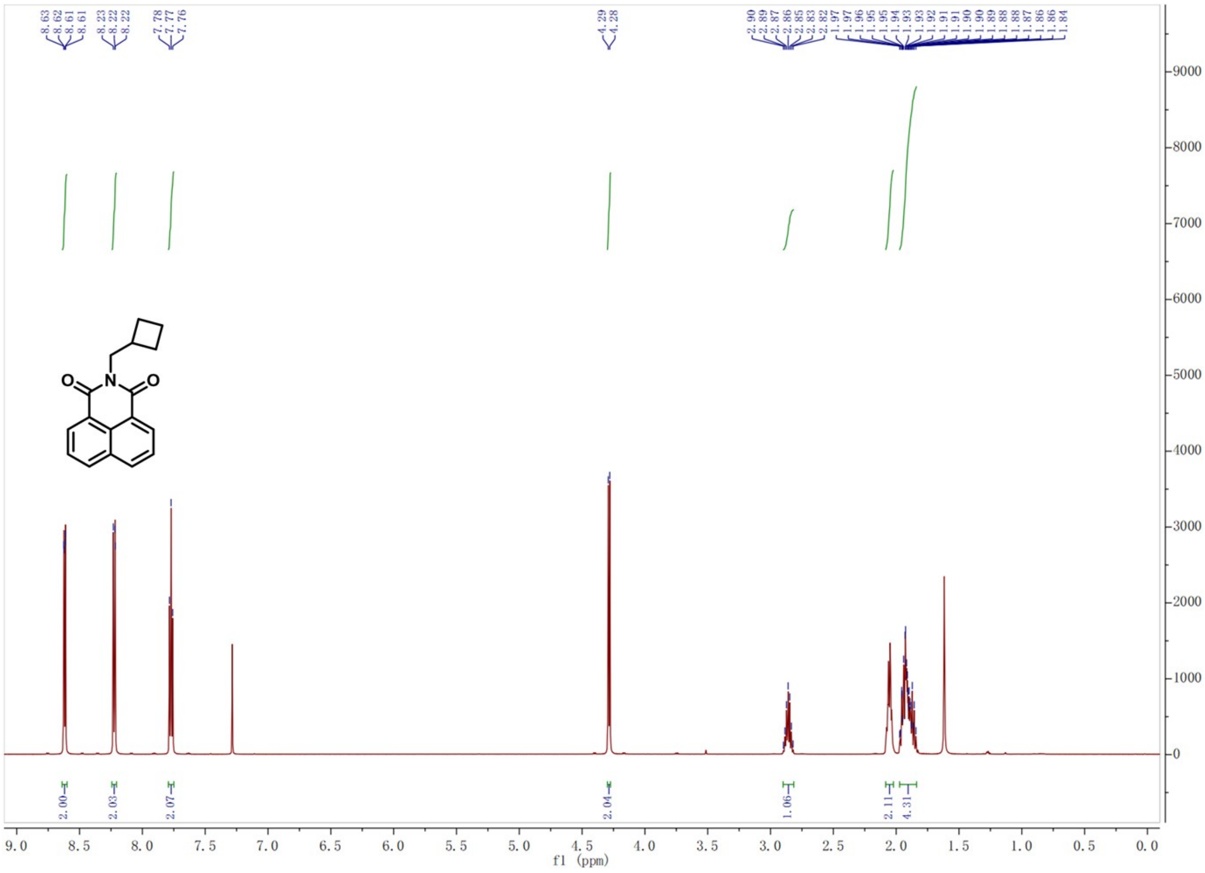

Fig. S5. ^{1}H NMR spectra of probe **6**.

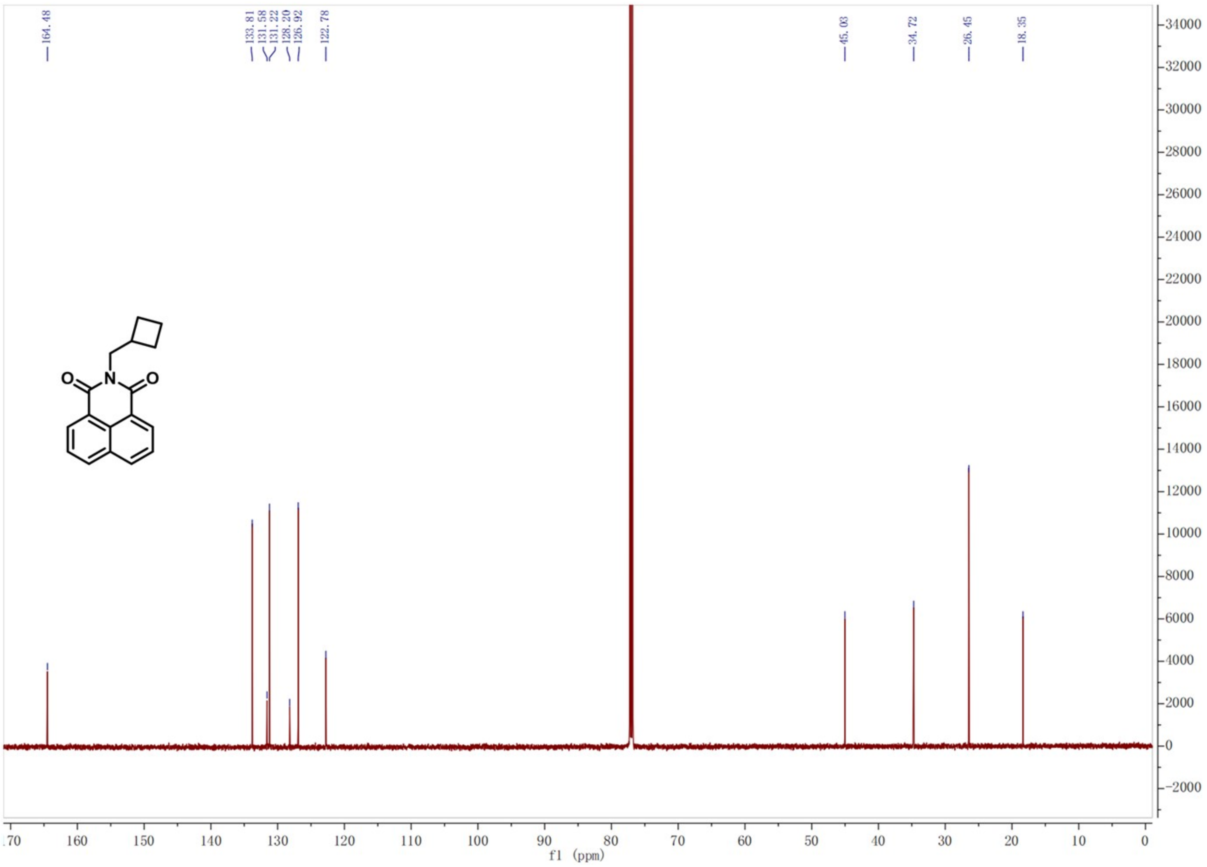

Fig. S6. ^{13}C NMR spectra of probe **6**.

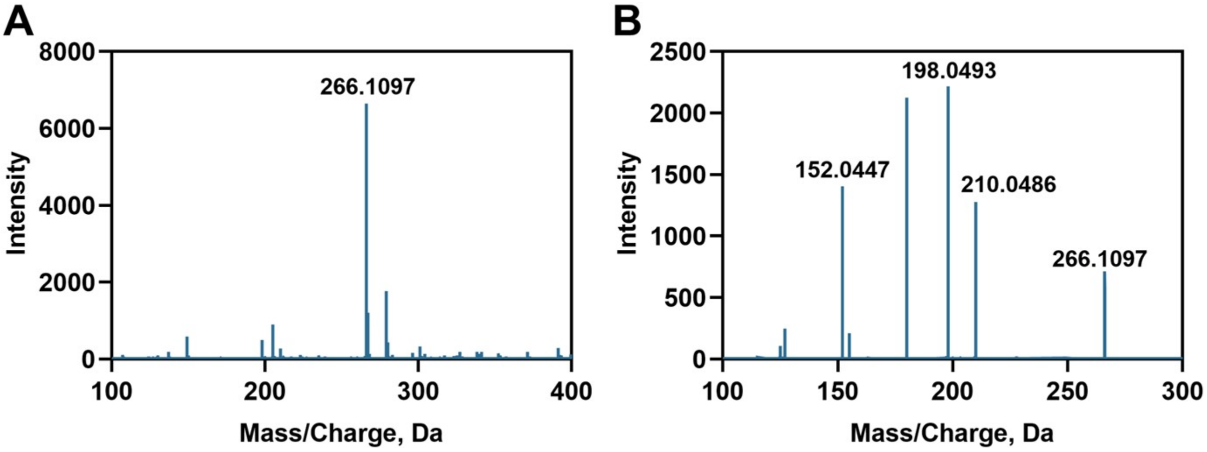

Fig. S7. MS (A) and MS/MS (B) spectra of probe **6**.

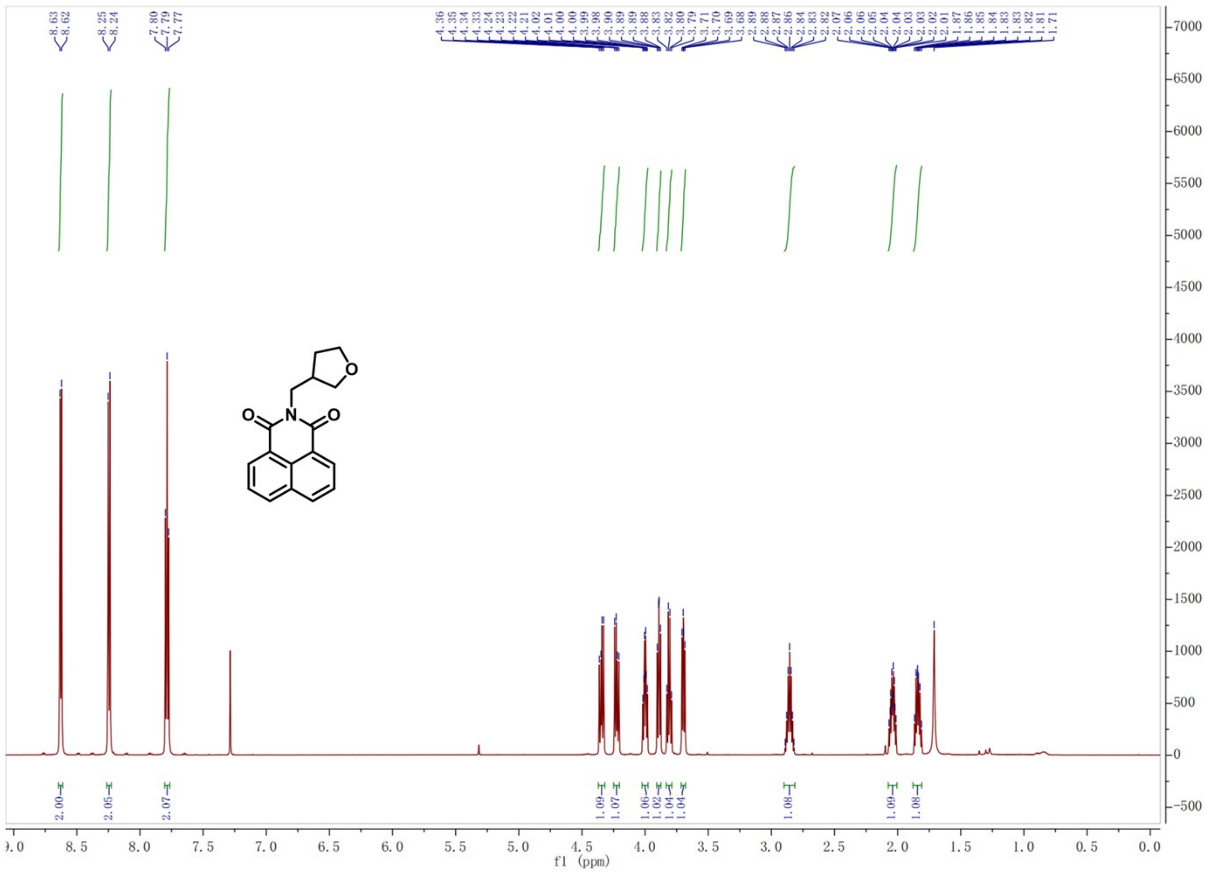

Fig. S8. ^{1}H NMR spectra of probe **8**.

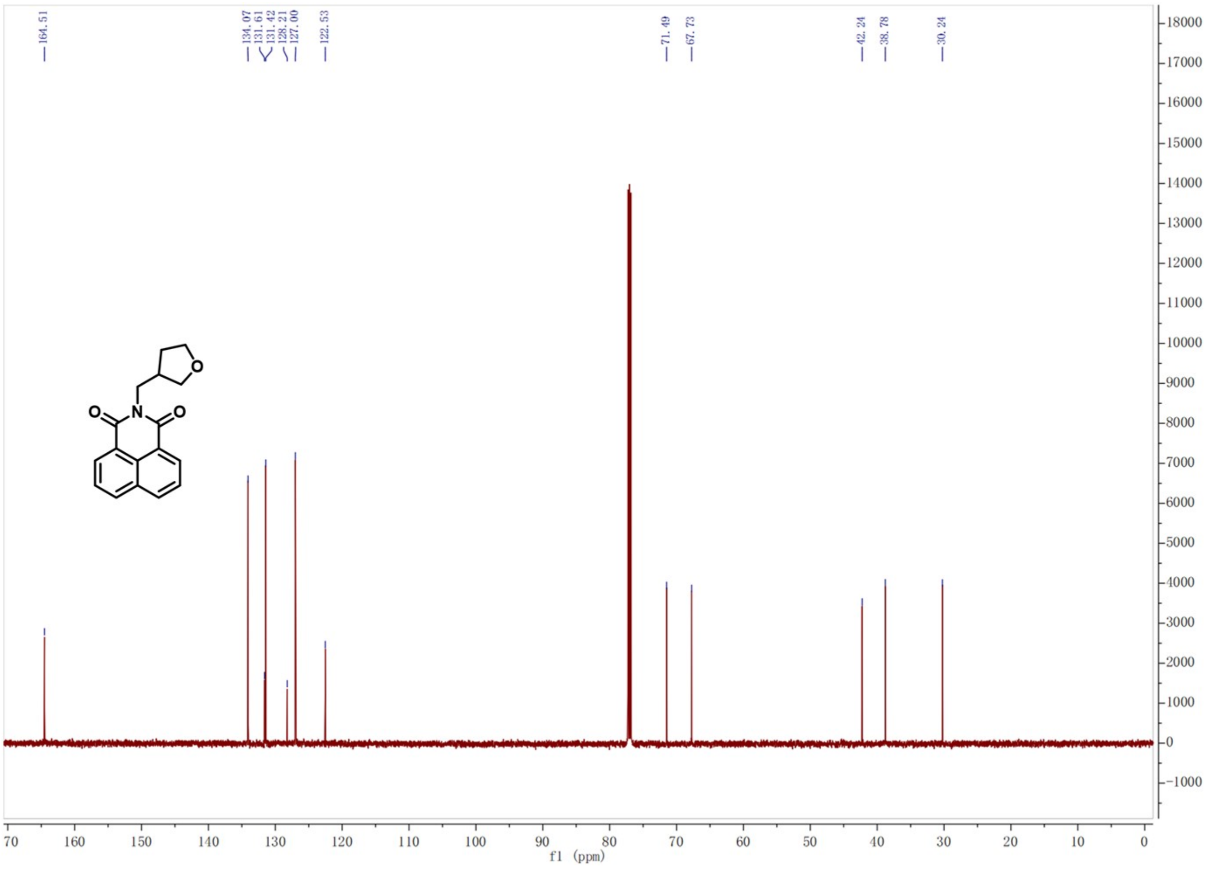

Fig. S9. ^{13}C NMR spectra of probe **8**.

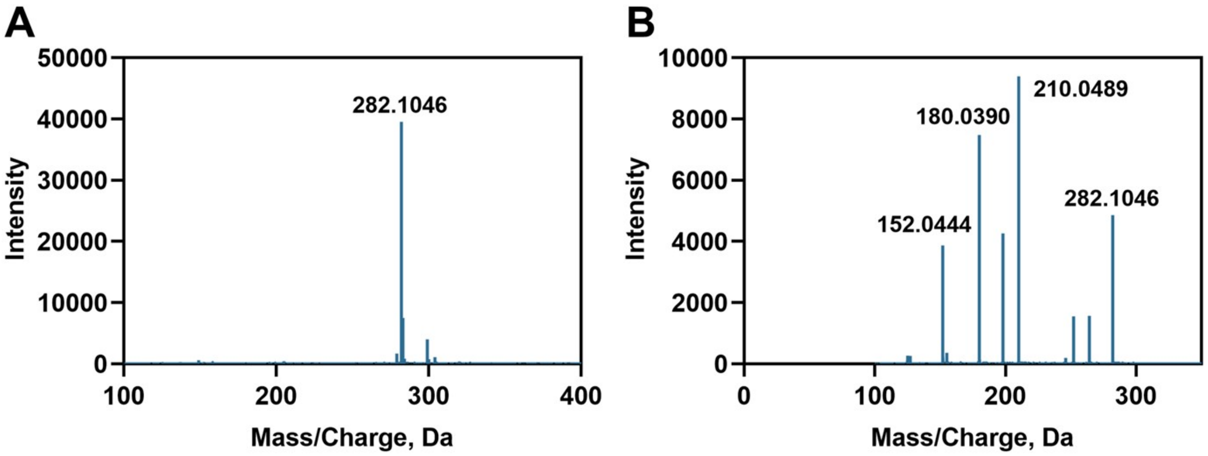

Fig. S10. MS (A) and MS/MS (B) spectra of probe **8**.

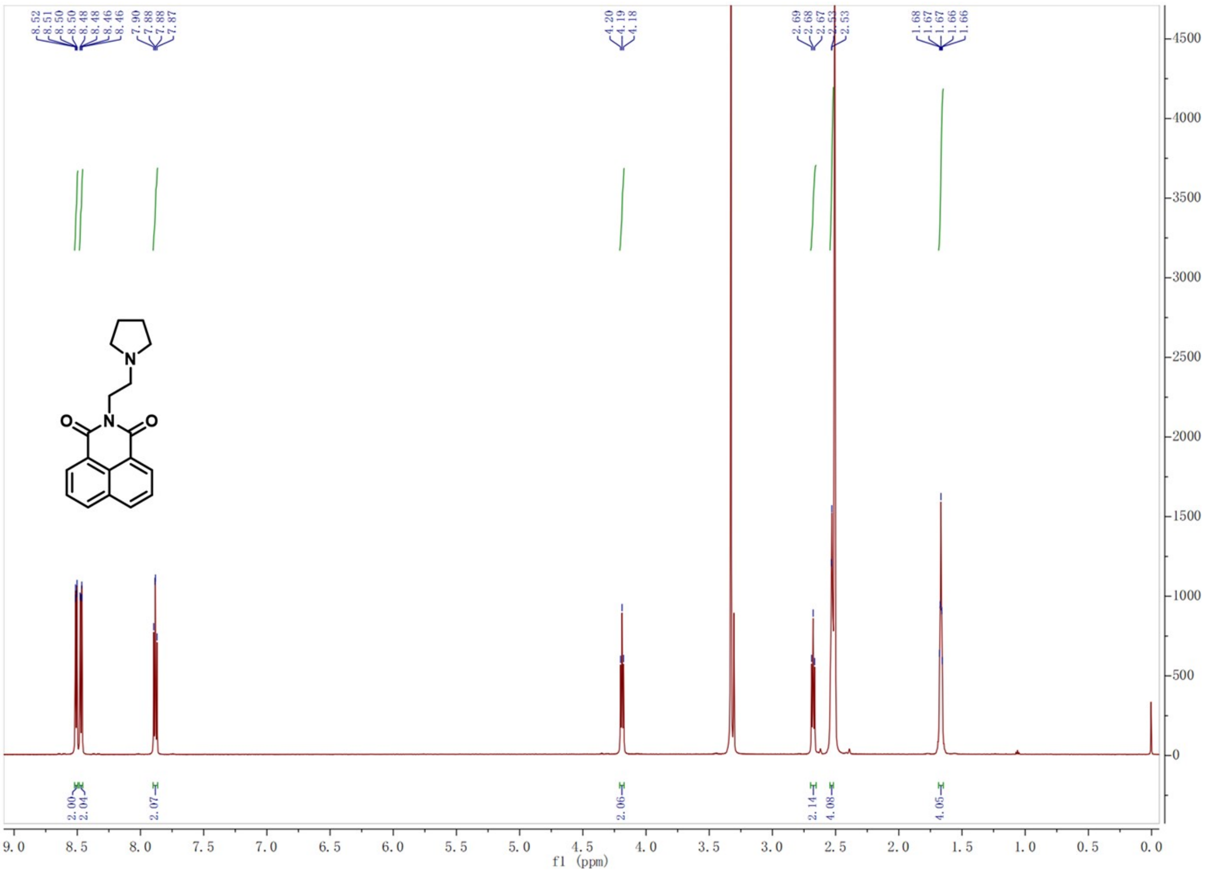

Fig. S11. ^{1}H NMR spectra of probe **10**.

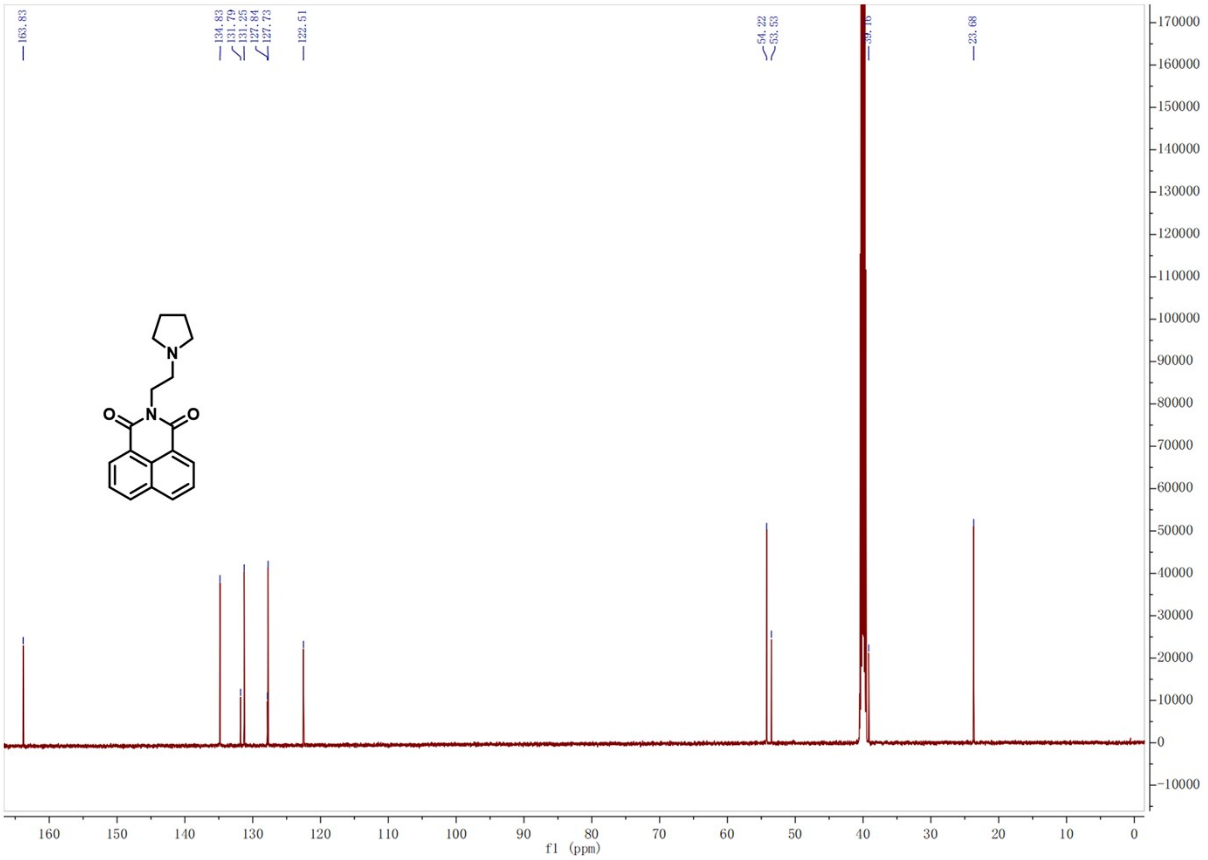

Fig. S12. ^{13}C NMR spectra of probe **10**.

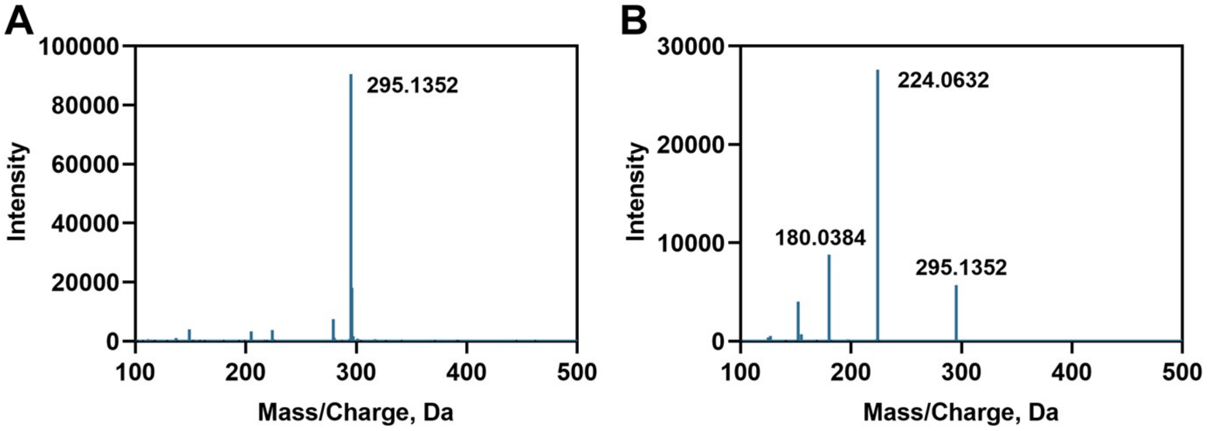

Fig. S13. MS (A) and MS/MS (B) spectra of probe **10**.

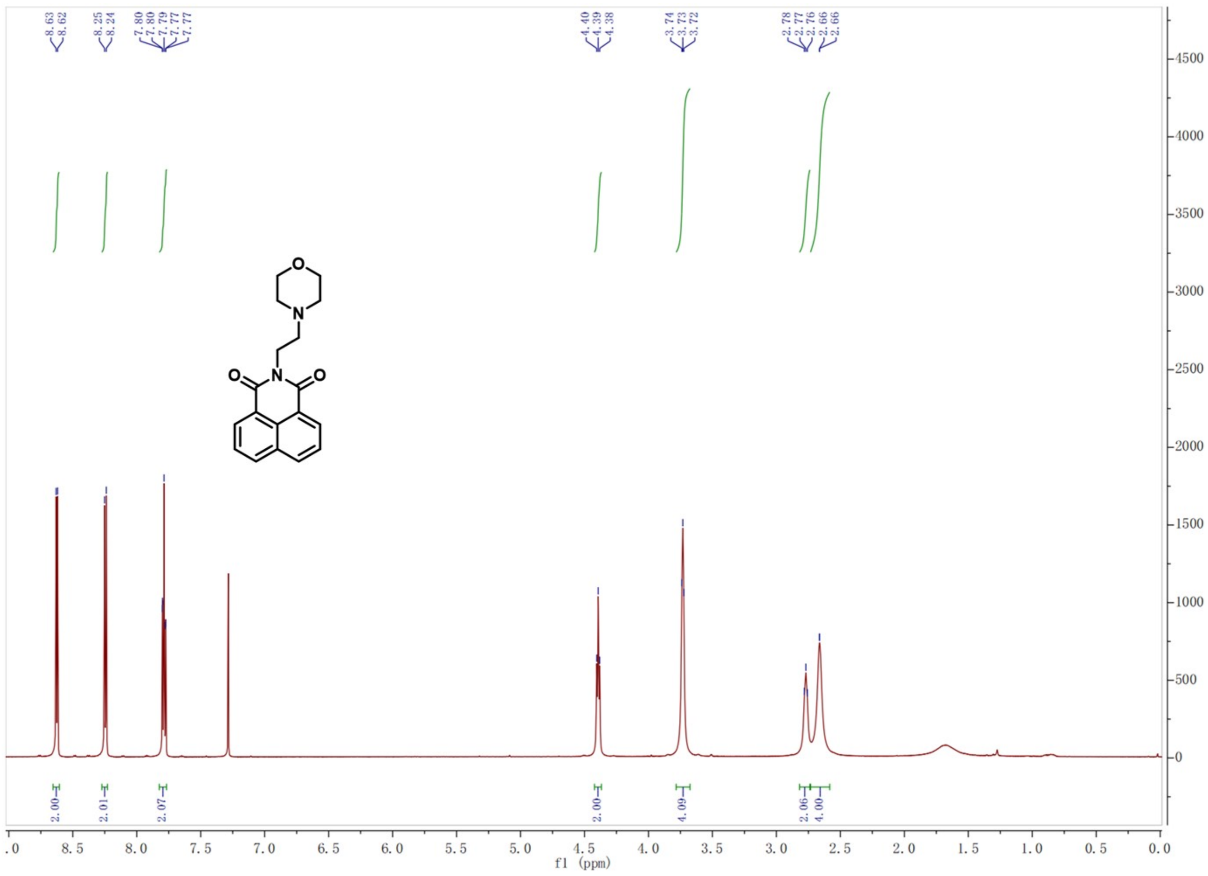

Fig. S14. ^{1}H NMR spectra of probe **11**.

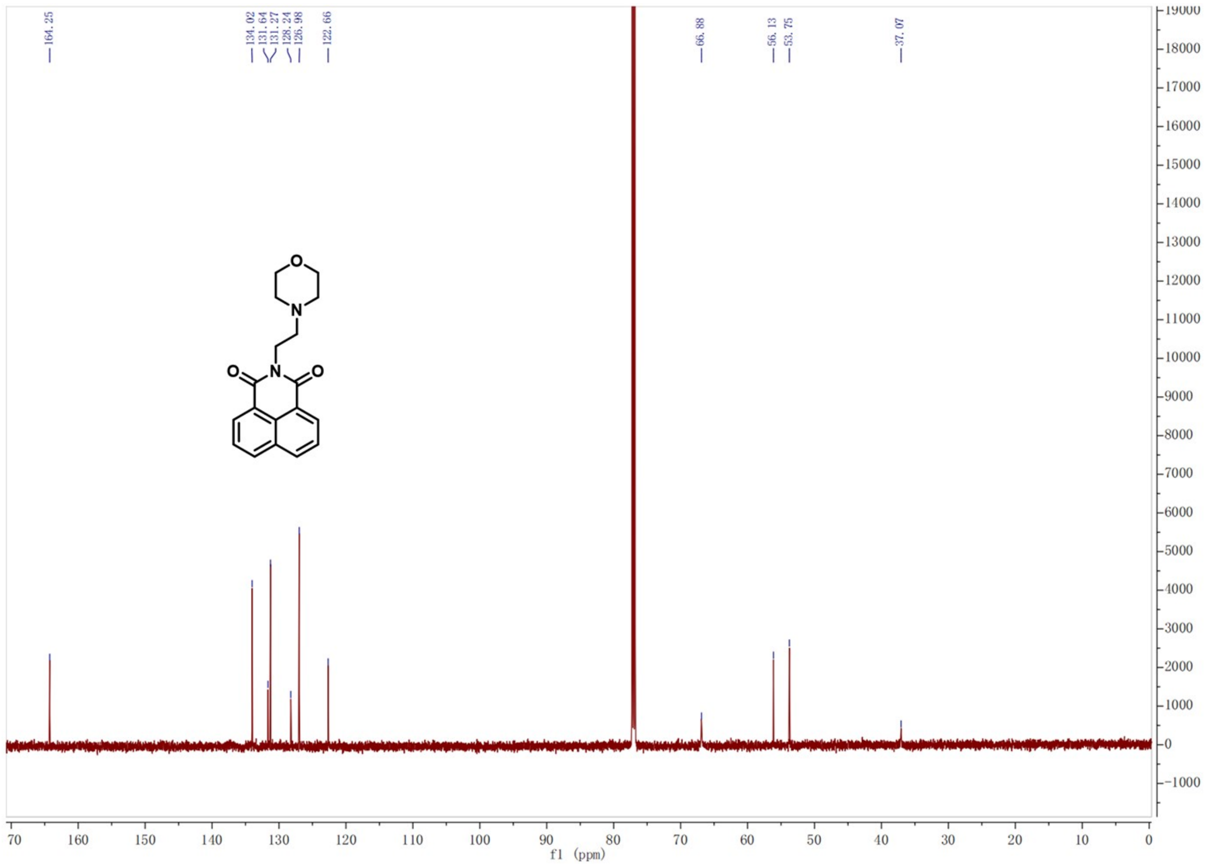

Fig. S15. ^{13}C NMR spectra of probe **11**.

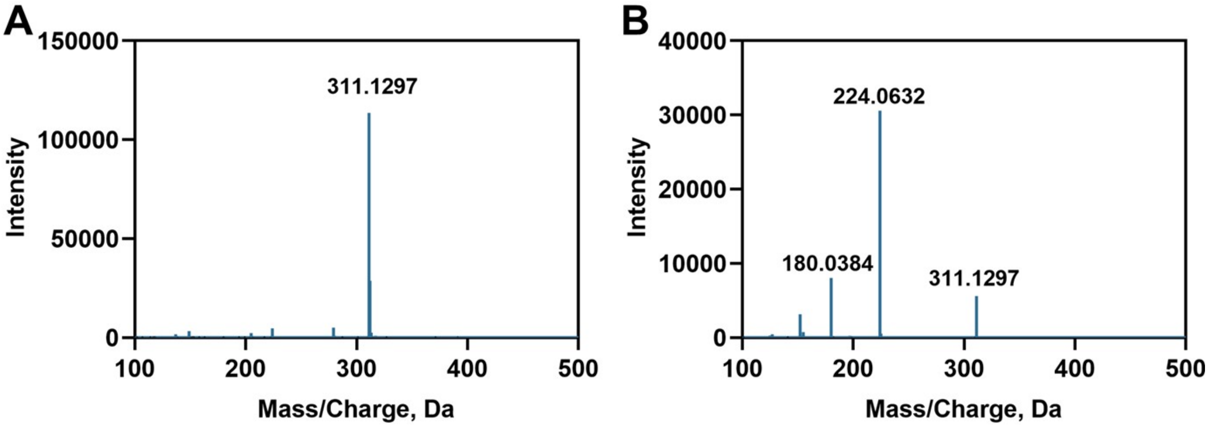

Fig. S16. MS (A) and MS/MS (B) spectra of probe **11**.

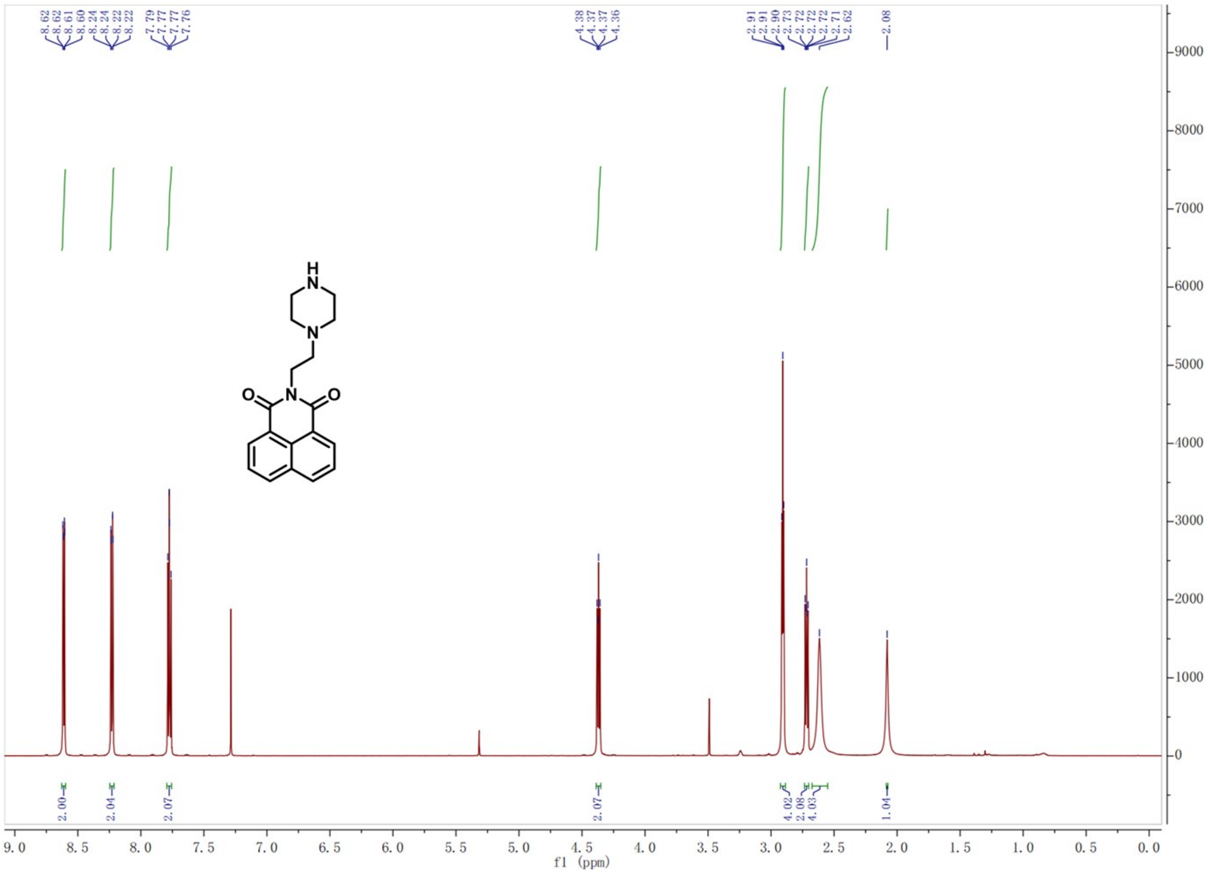

Fig. S17. ^{1}H NMR spectra of probe **12**.

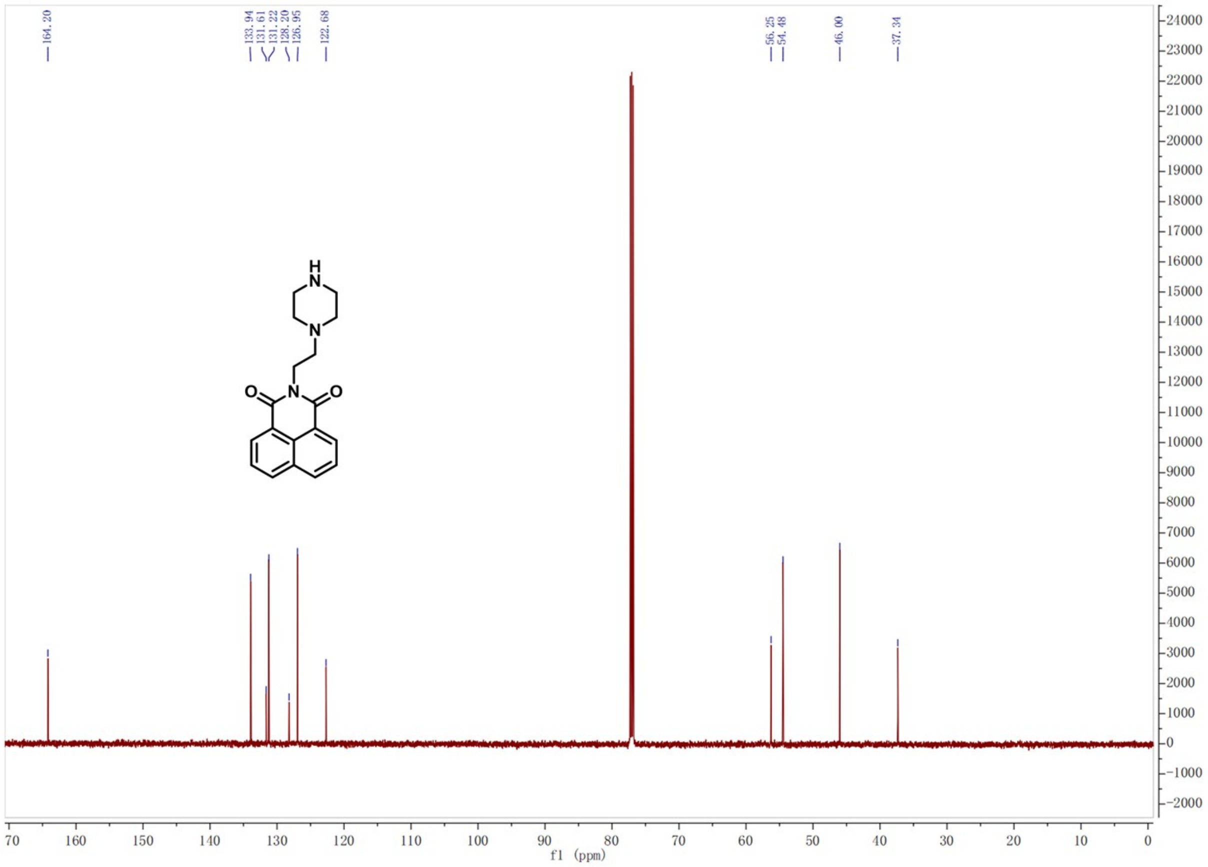

Fig. S18. ^{13}C NMR spectra of probe **12**.

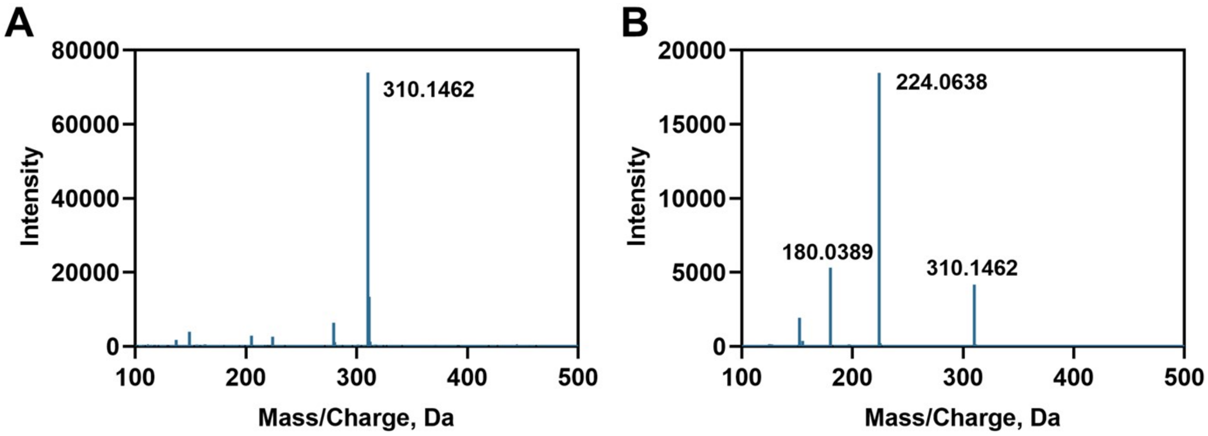

Fig. S19. MS (A) and MS/MS (B) spectra of probe **12**.

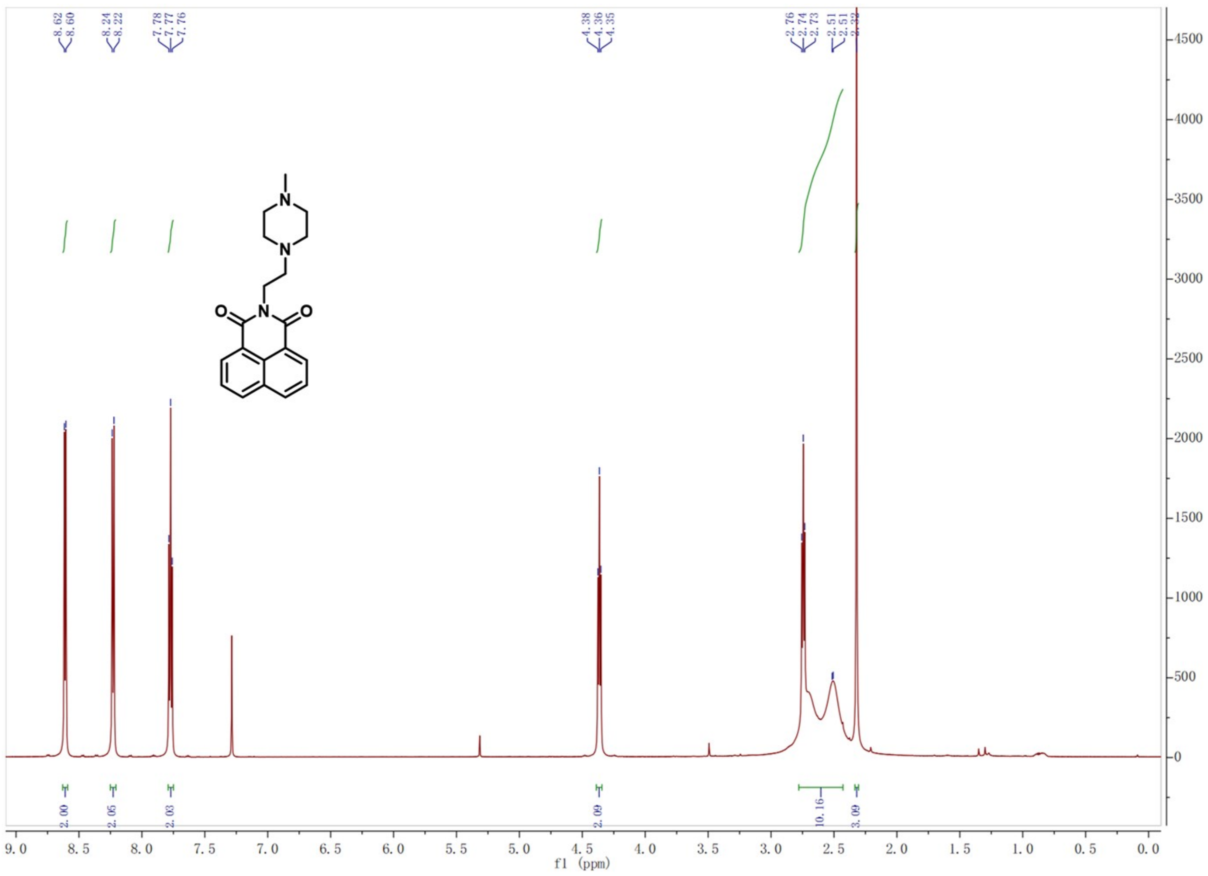

Fig. S20. ^{1}H NMR spectra of probe **13**.

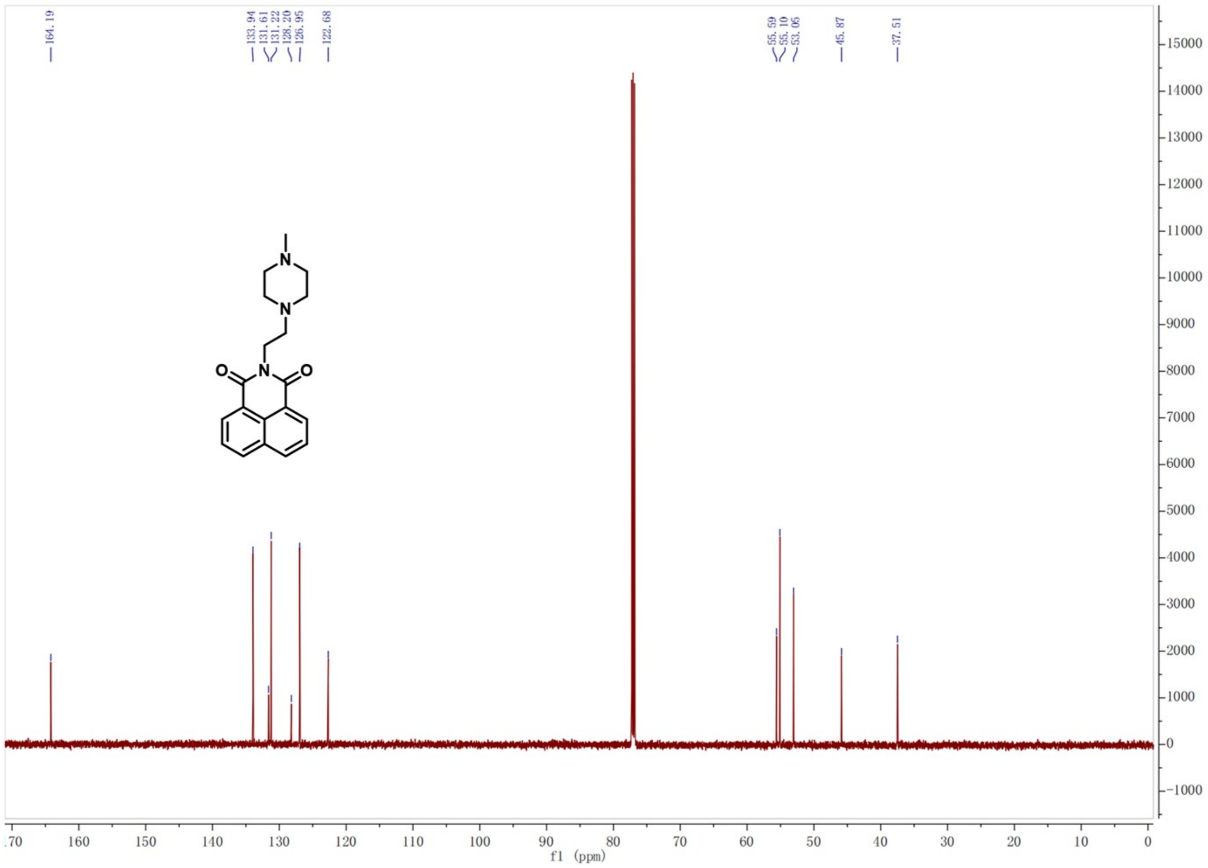

Fig. 21. ^{13}C NMR spectra of probe **13**.

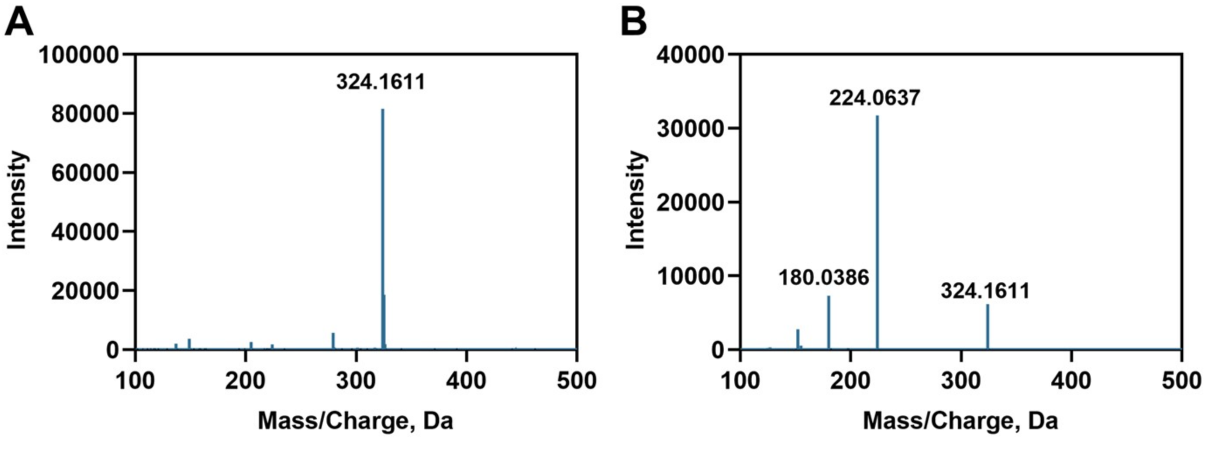

Fig. S22. MS (A) and MS/MS (B) spectra of probe **13**.

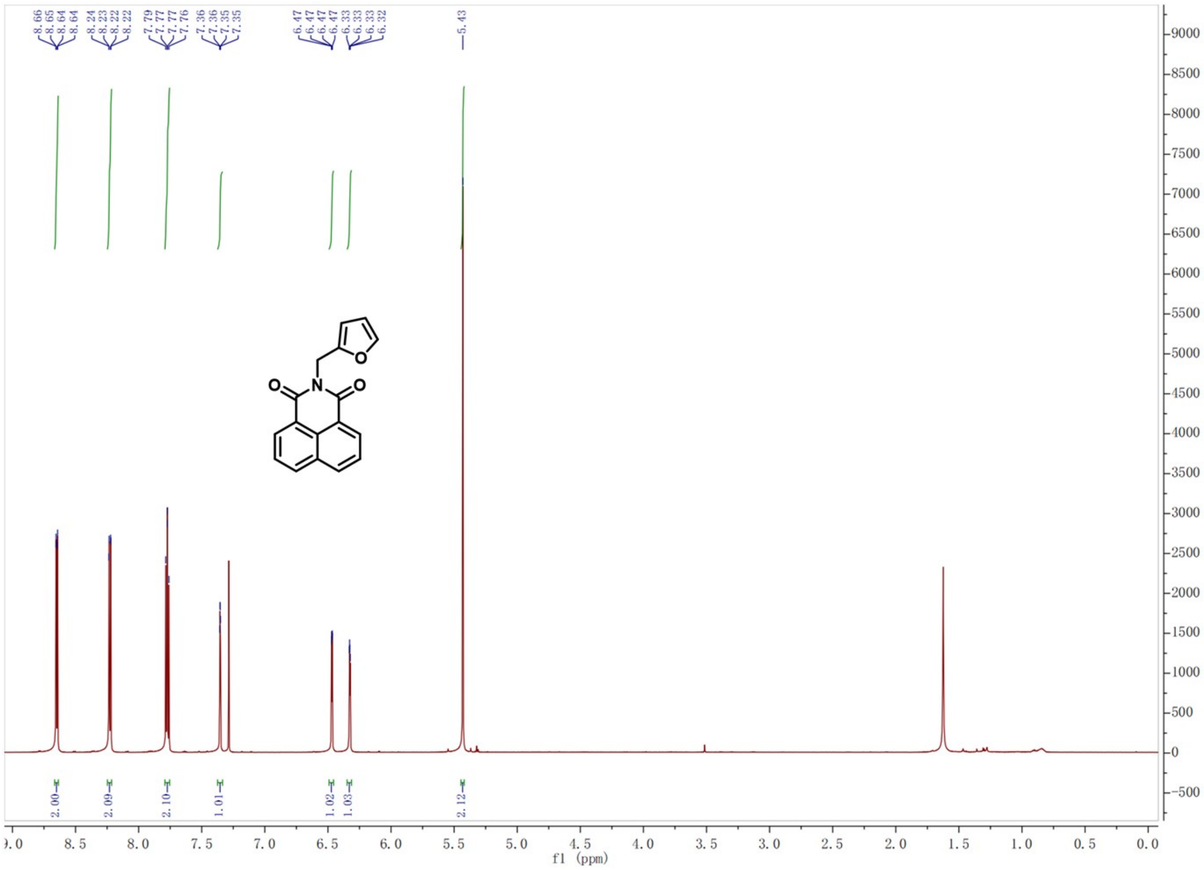

Fig. S23. ^{1}H NMR spectra of probe **15**.

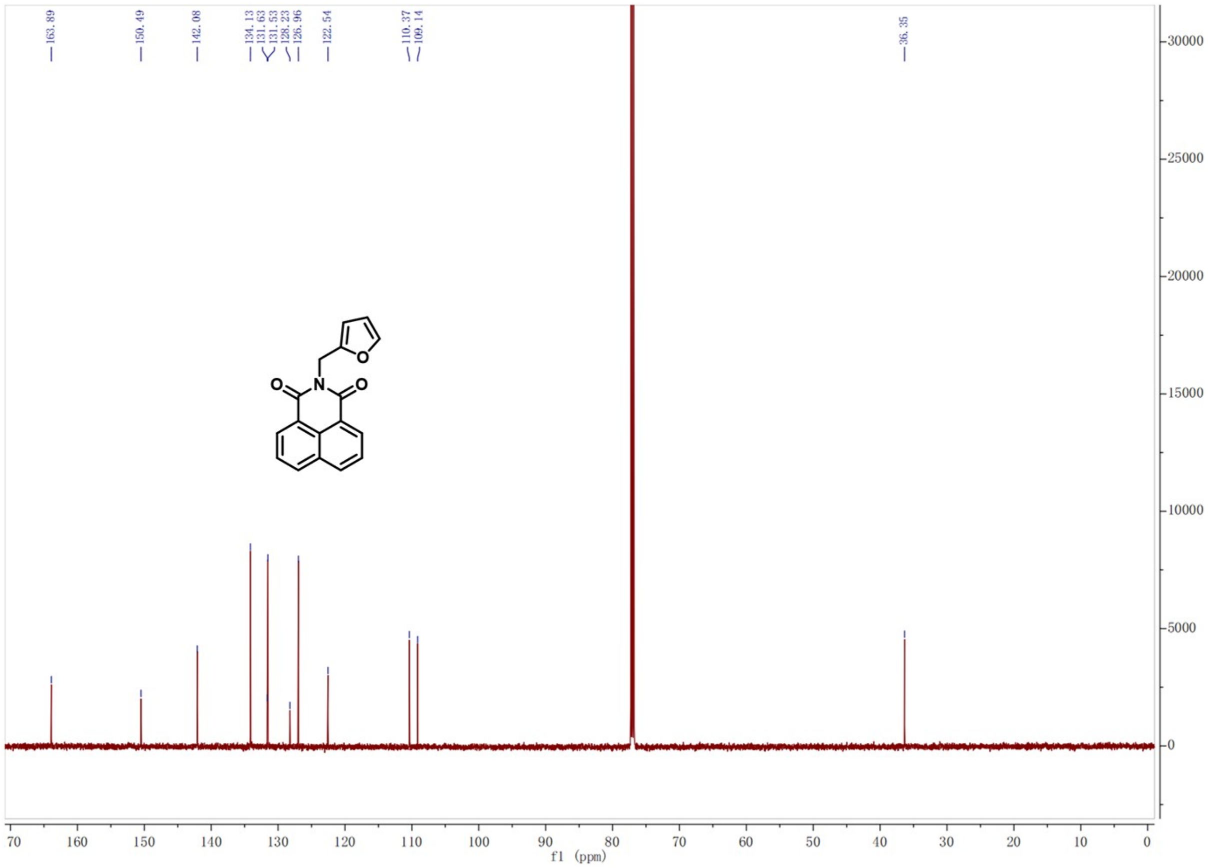

Fig. S24. ^{13}C NMR spectra of probe **15**.

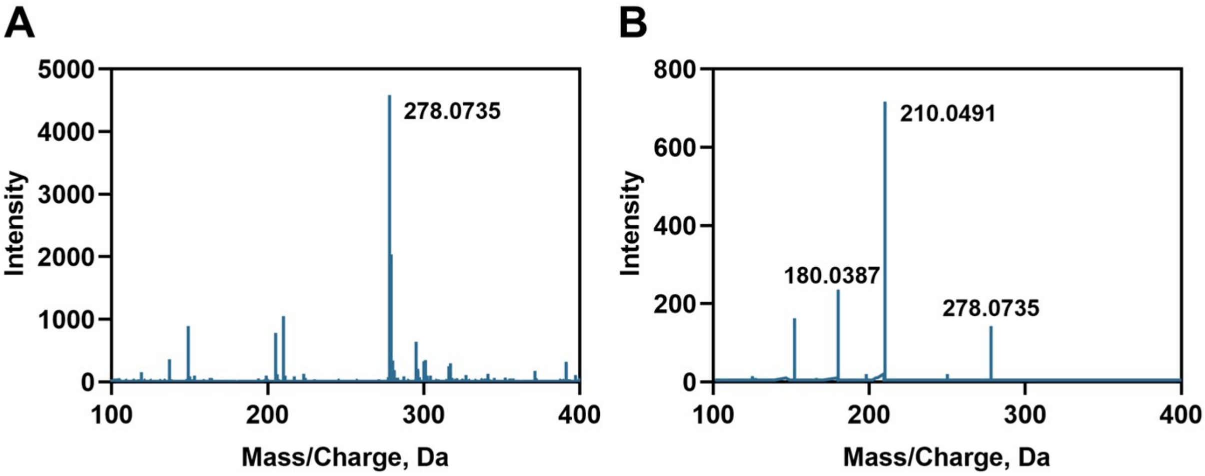

Fig. S25. MS (A) and MS/MS (B) spectra of probe **15**.

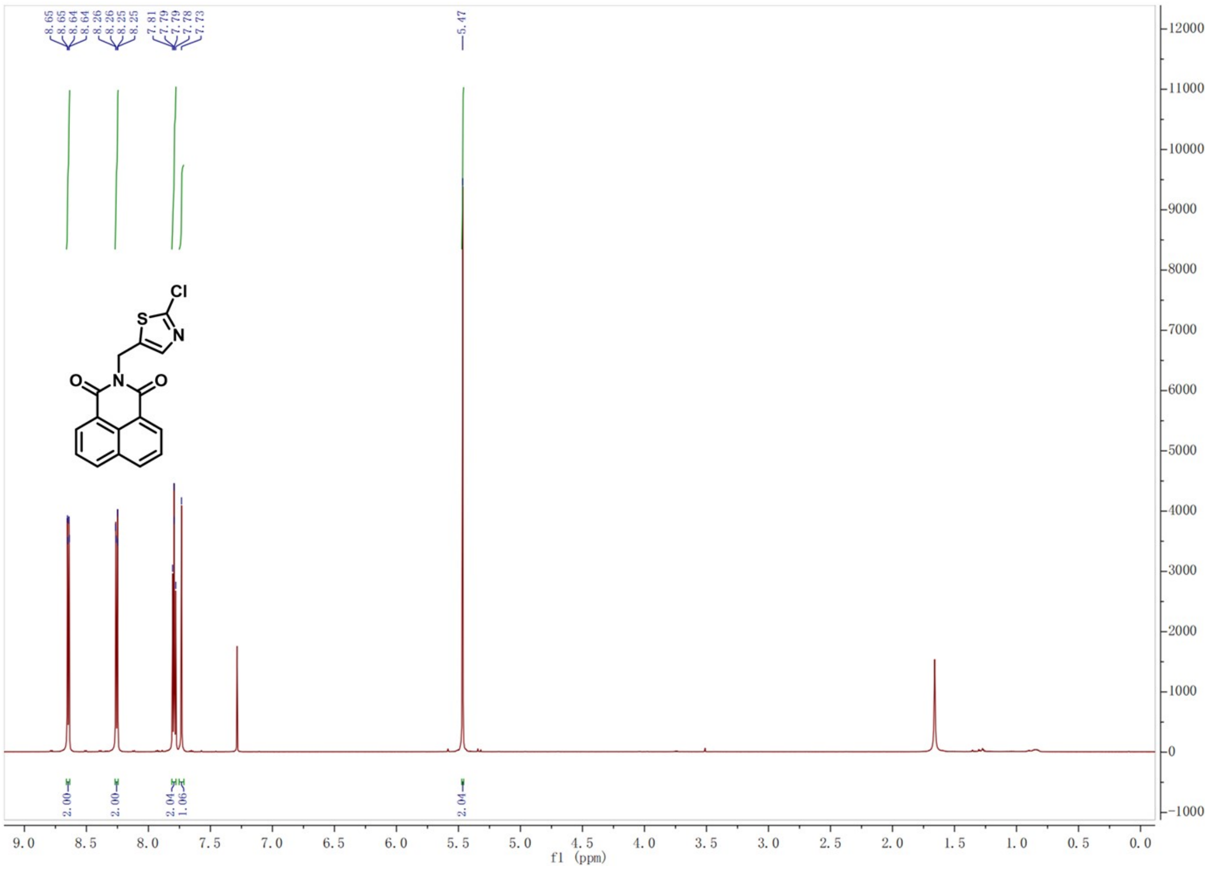

Fig. S26. ^{1}H NMR spectra of probe **18**.

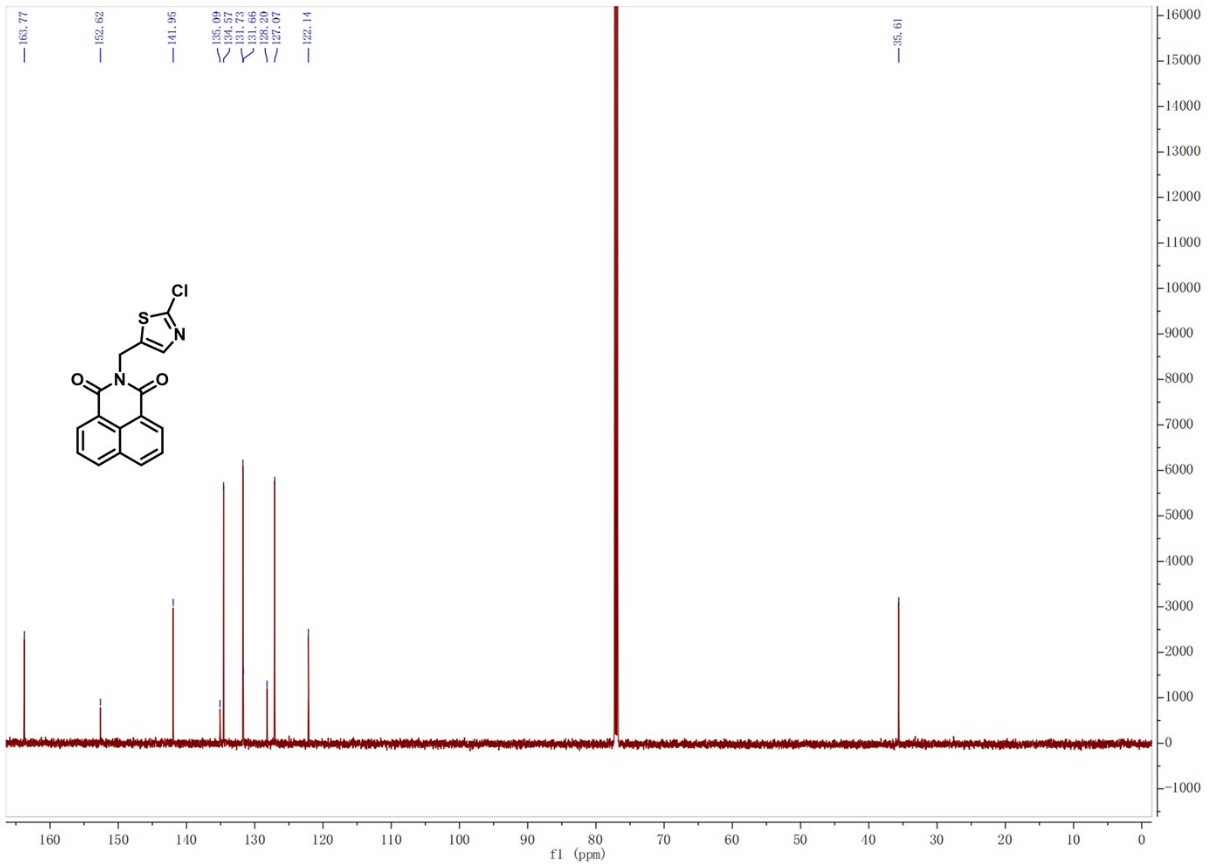

Fig. S27. ^{13}C NMR spectra of probe **18**.

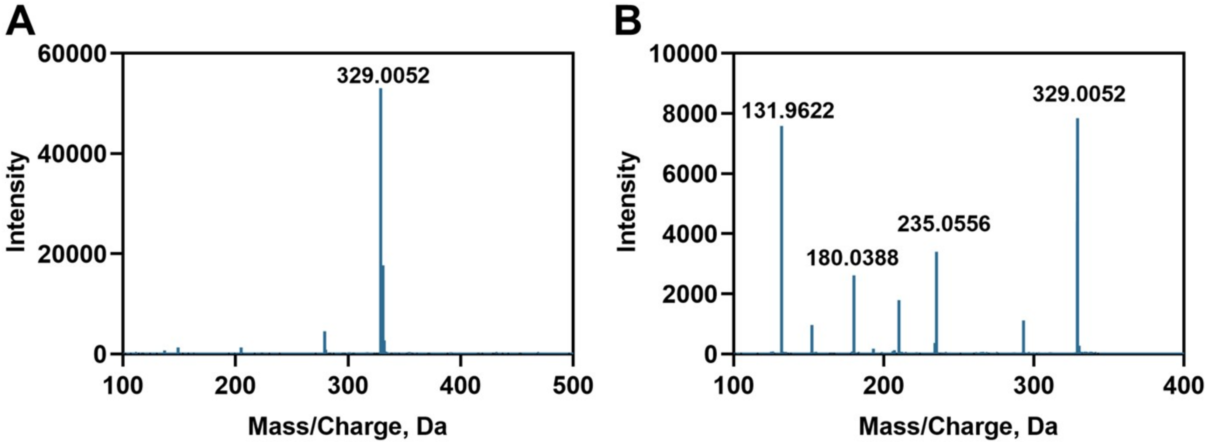

Fig. S28. MS (A) and MS/MS (B) spectra of probe **18**.

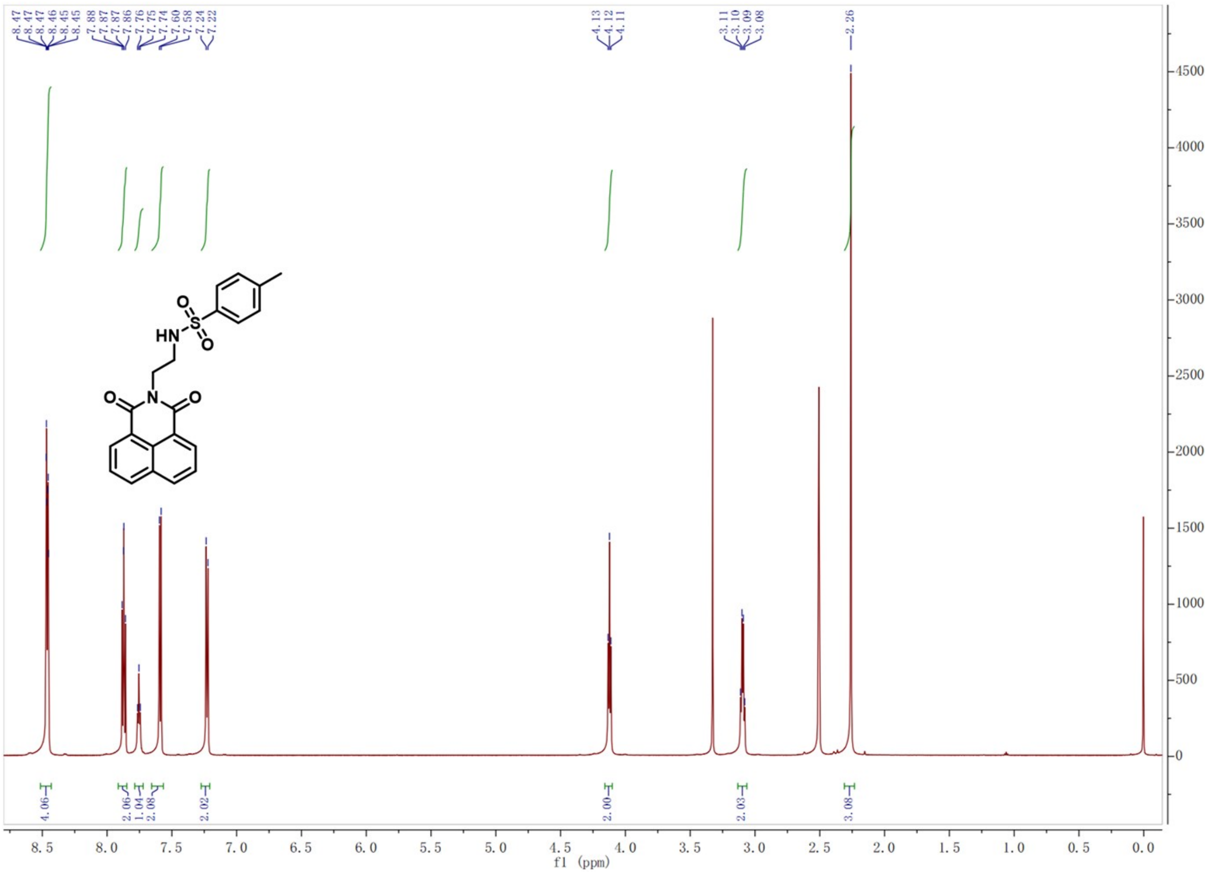

Fig. S29. ^{1}H NMR spectra of probe **22**.

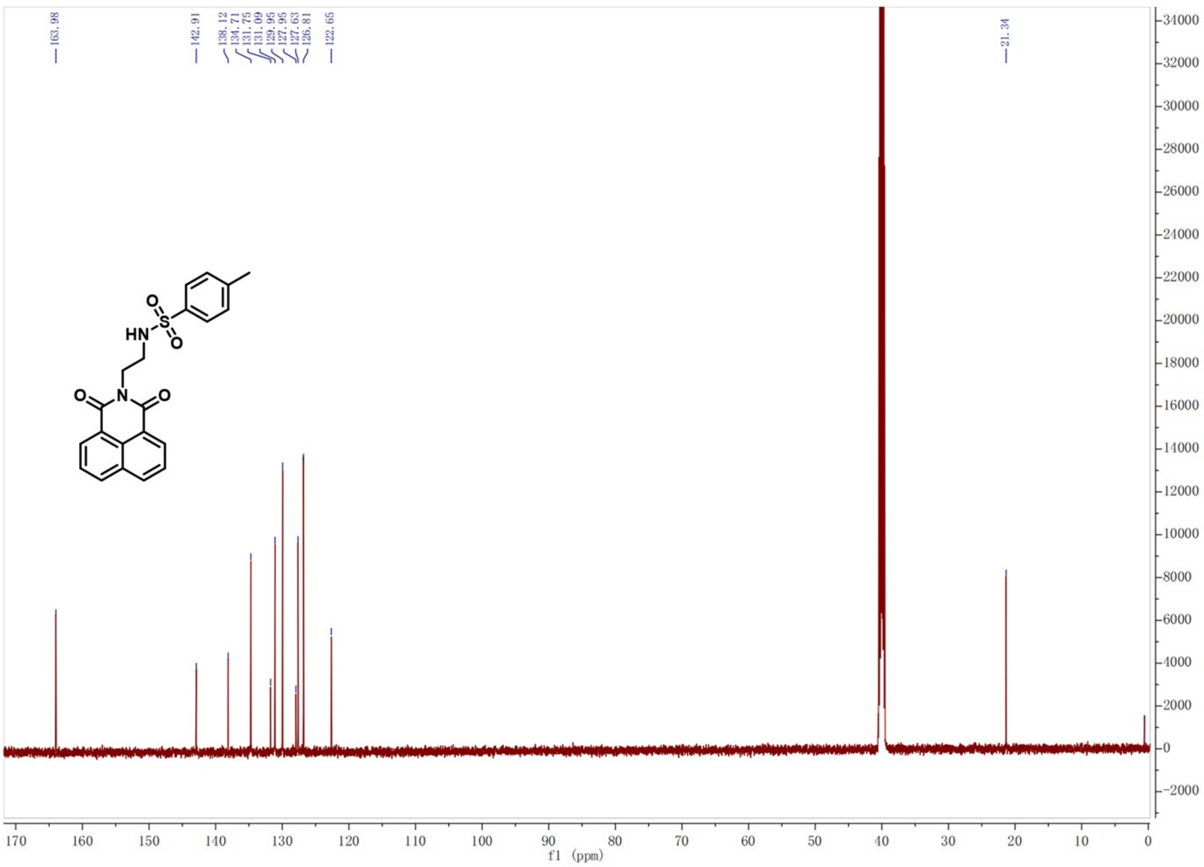

Fig. S30. ^{13}C NMR spectra of probe **22**.

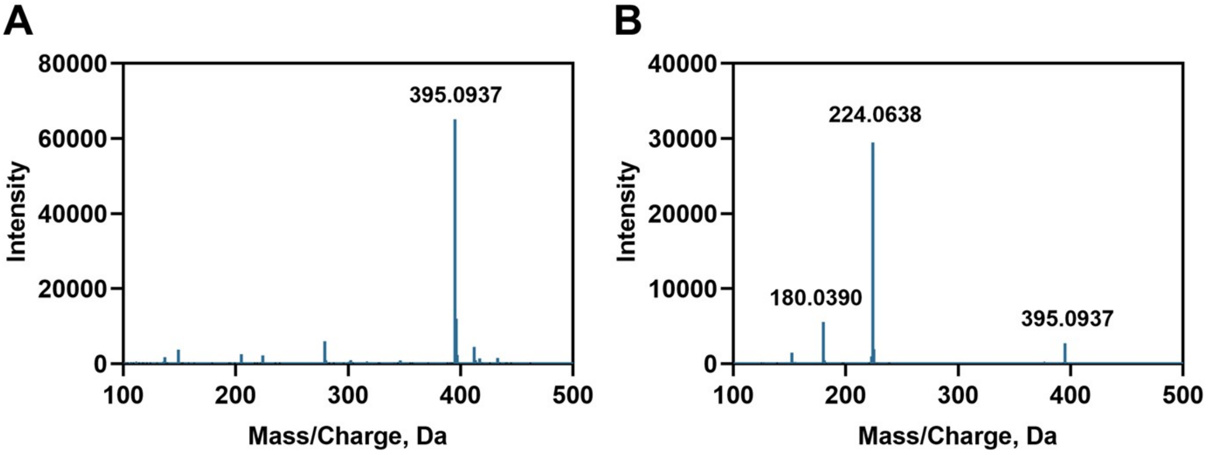

Fig. S31. MS (A) and MS/MS (B) spectra of probe **22**.

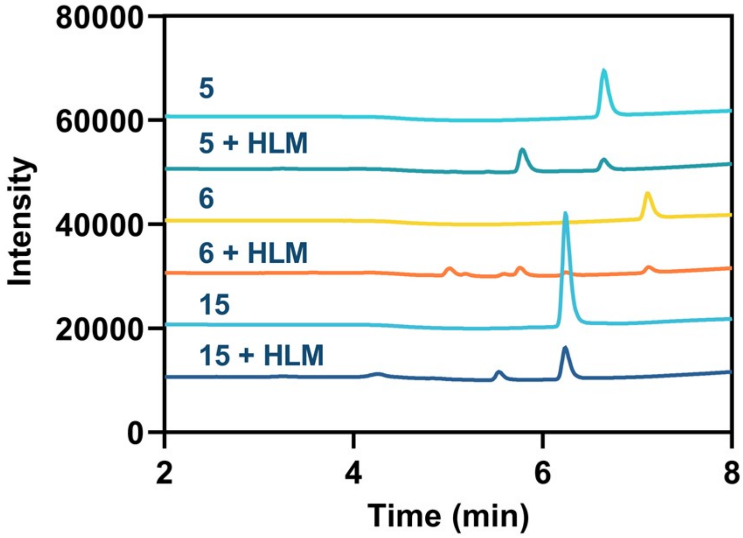

Fig. S32. Metabolic profiling analysis of probe 5, 6, and 15 in HLM.

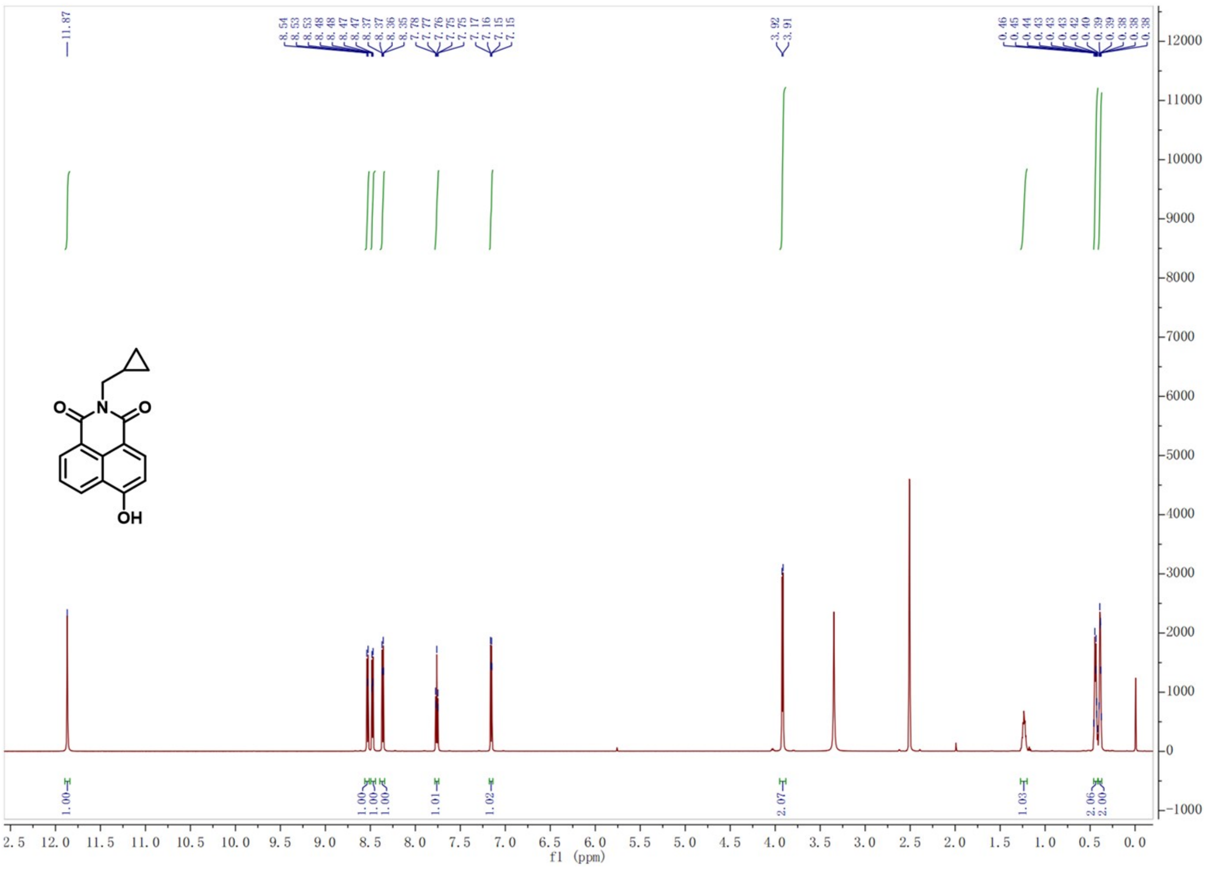

Fig. S33. ^{1}H NMR spectra of **HNCN**.

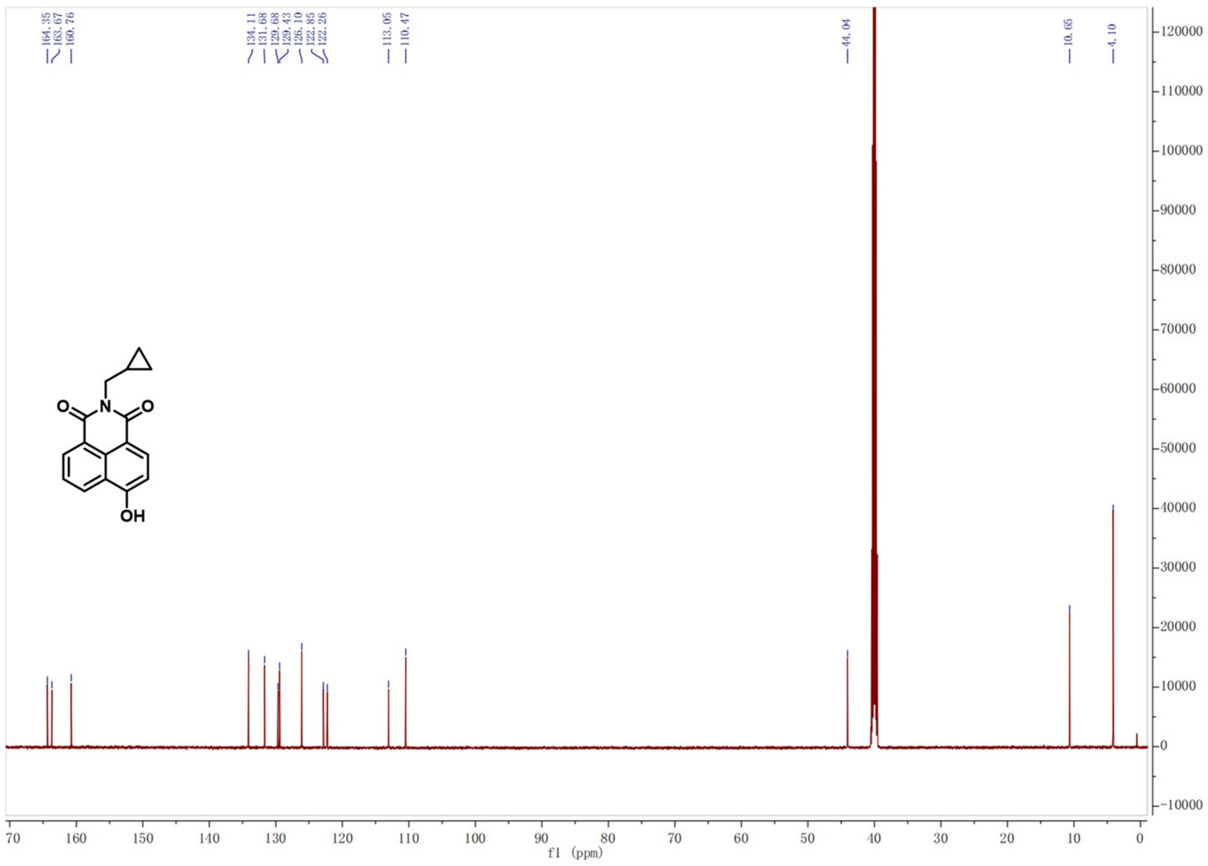

Fig. S34. ^{13}C NMR spectra of **HNCN**.

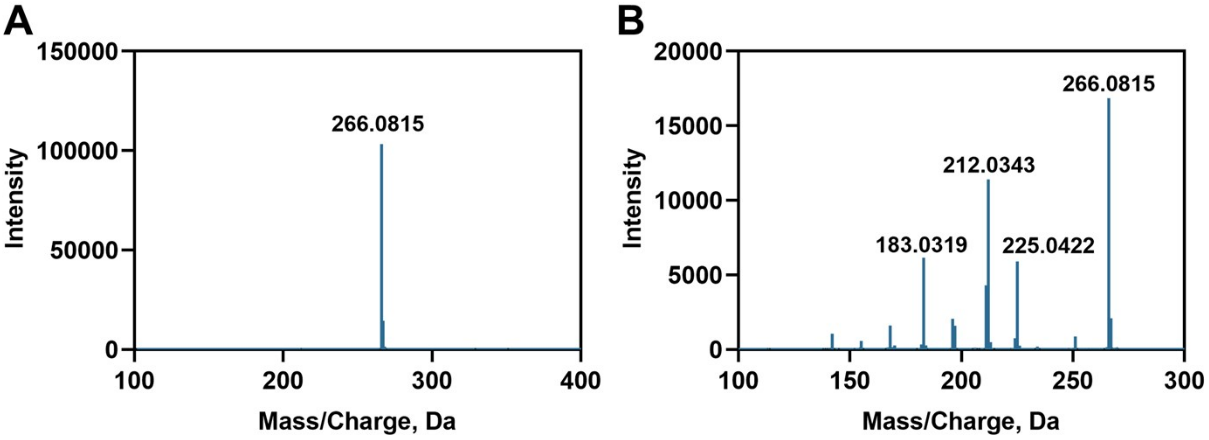

Fig. S35. MS (A) and MS/MS (B) spectra of **HNCN**.

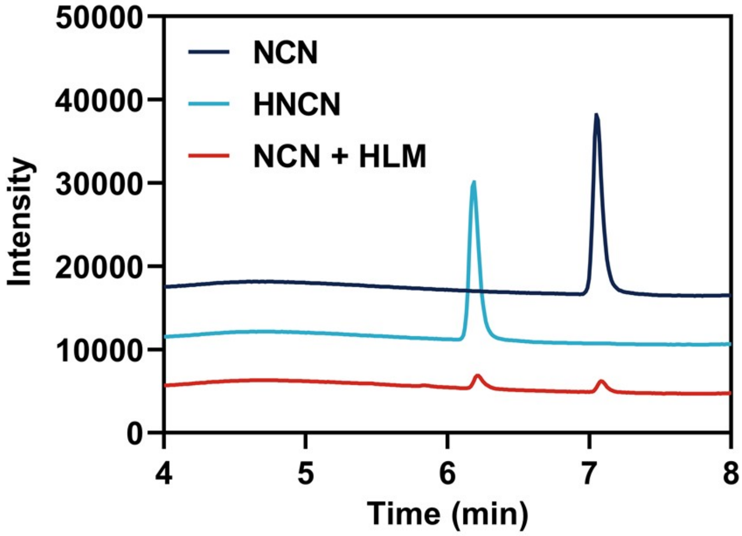

Fig. S36. LC-UV chromatography profiles of **NCN** hydroxylation.

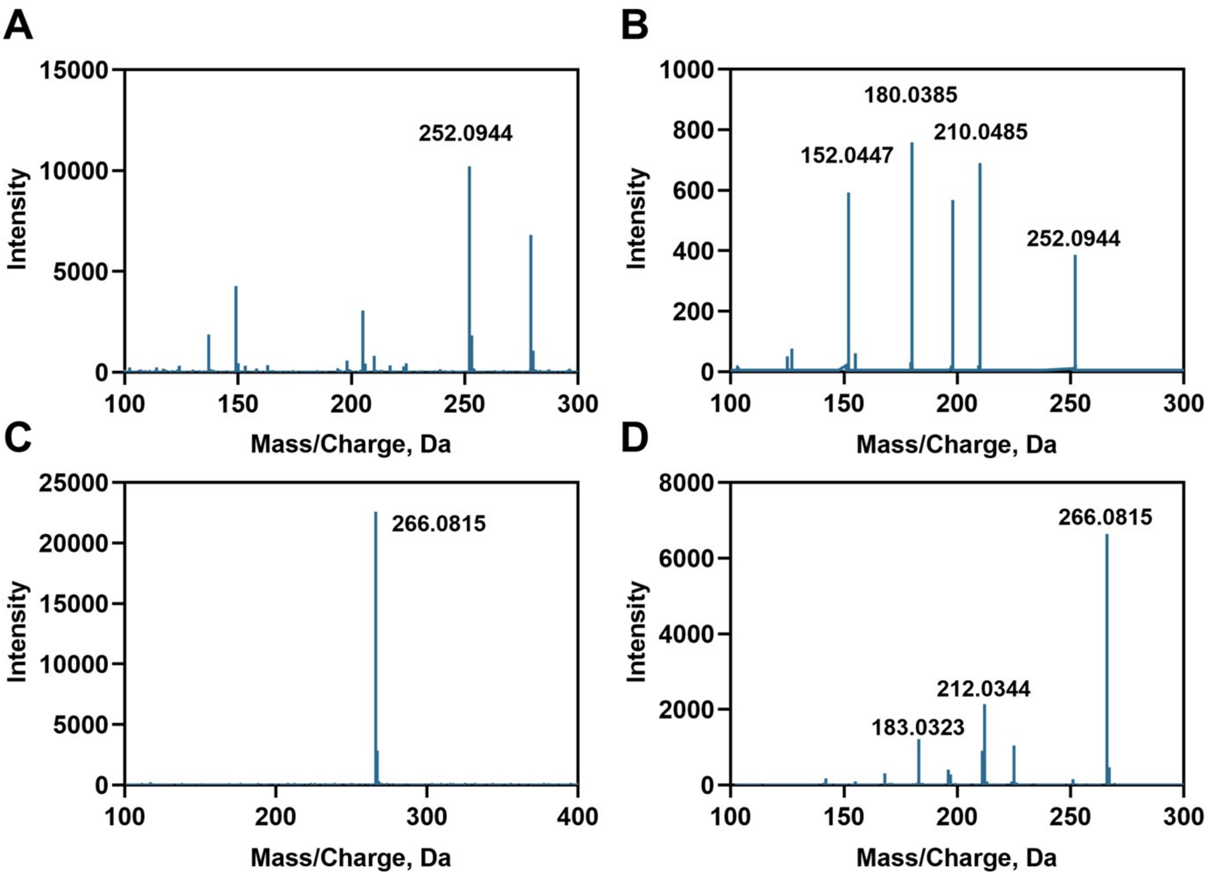

Fig. S37. (A-B) MS (A) and MS/MS (B) spectra of CYP3A4 substrate **NCN** with the quasi-molecular ion at *m/z* 252.10 under positive ion mode. (C-D) MS (C) and MS/MS (D) spectra of hydroxylation product **HNCN** with the quasi-molecular ion at *m/z* 266.08 under negative ion mode.

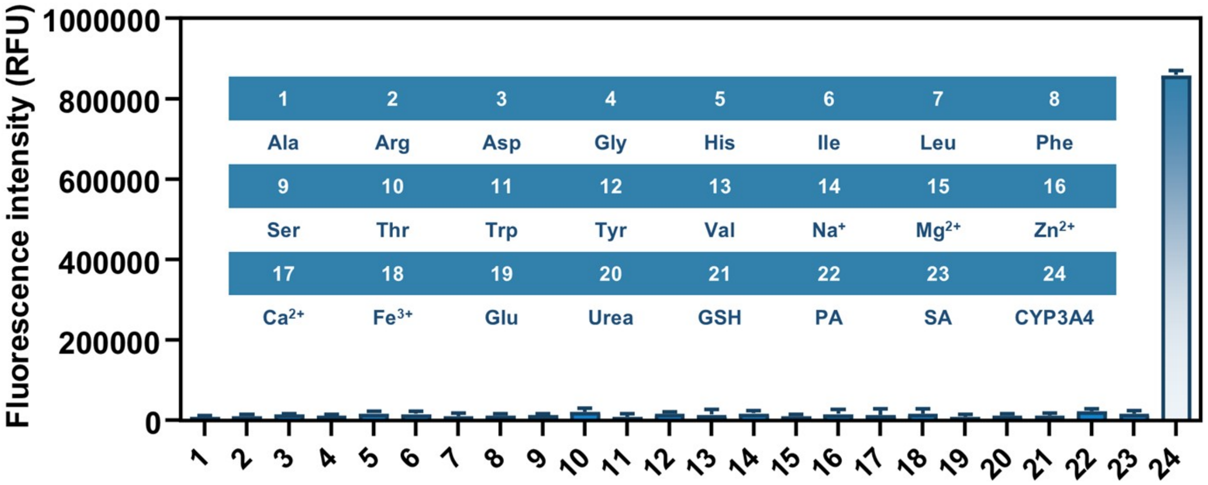

Fig. S38. Fluorescence response of **NCN** towards various endogenous compounds (10 μM). Note: PA, palmitic acid; SA, stearic acid.

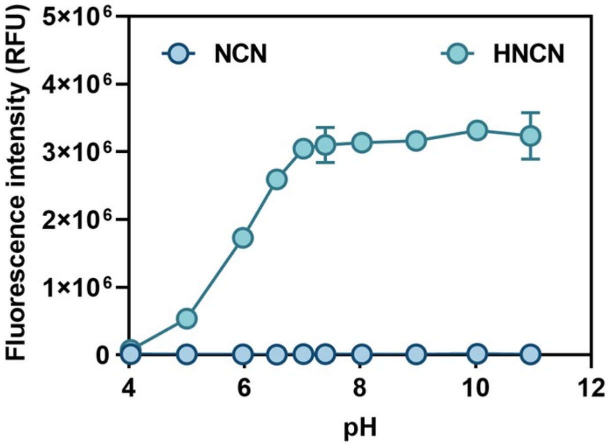

Fig. S39. Fluorescence response of **NCN** and **HNCN** under various pH conditions.

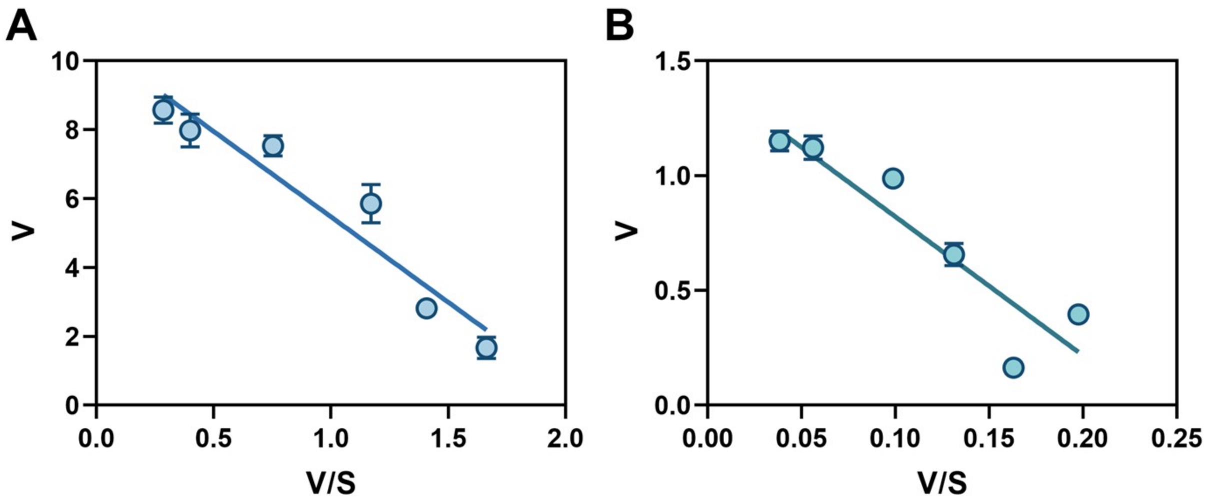

Fig. S40. The Eadie-Hofstee plots of CYP3A4 (A) and HLMs (B) catalyzed **NCN-4-*O*-**hydroxylation kinetics.

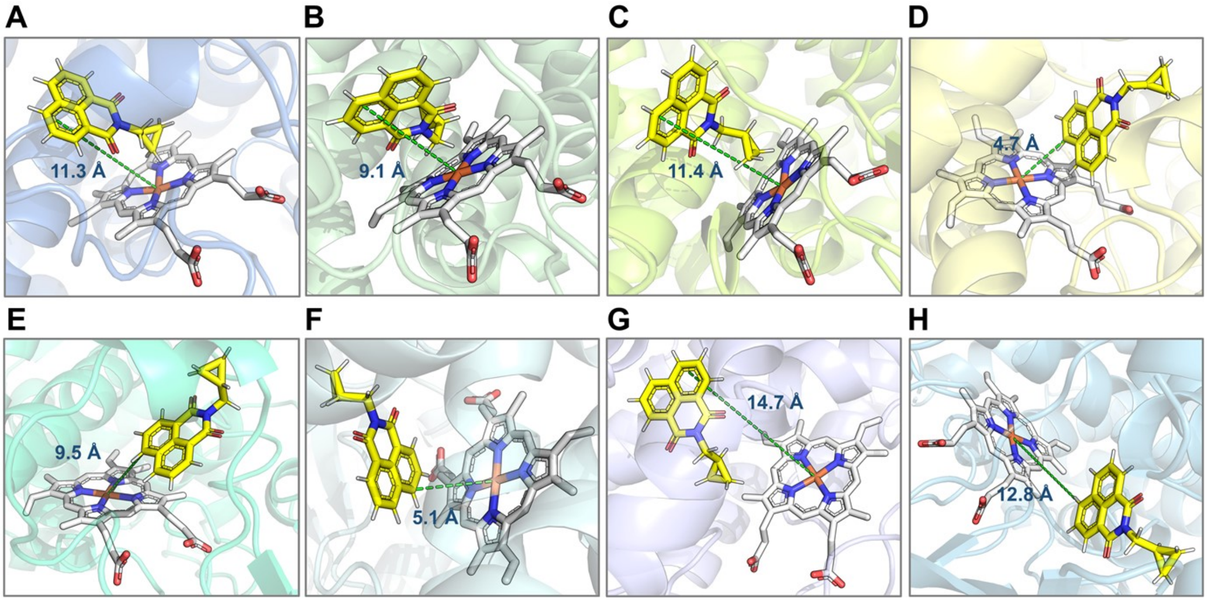

Fig. S41. Binding conformation of **NCN** in several important P450 isozymes, including CYP1A2 (PDB ID: 2HI4), CYP2A6 (PDB ID: 3T3R), CYP2B6 (PDB ID: 5UFG), CYP2C8 (PDB ID: 1PQ2), CYP2C9 (PDB ID: 1OG5), CYP2C19 (PDB ID: 4GQS), CYP2D6 (PDB ID: 4WNU), and CYP2E1 (PDB ID: 3E6I).

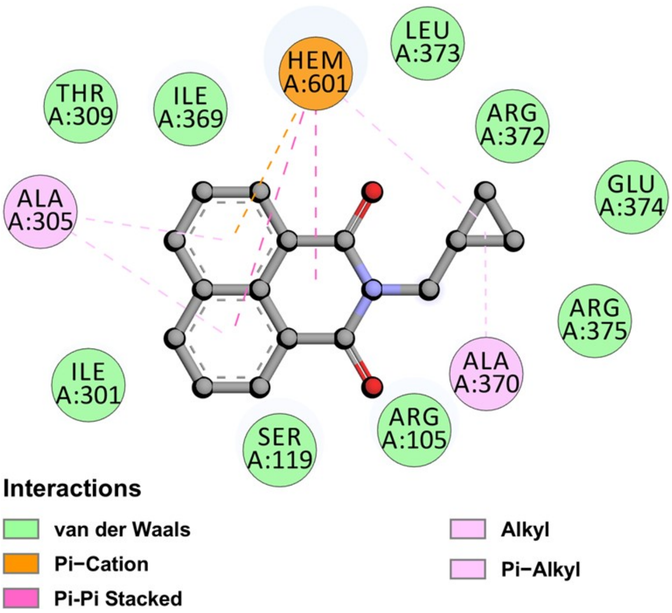

Fig. S42. Key interactions between **NCN** and the residues in the catalytic cavity of CYP3A4.

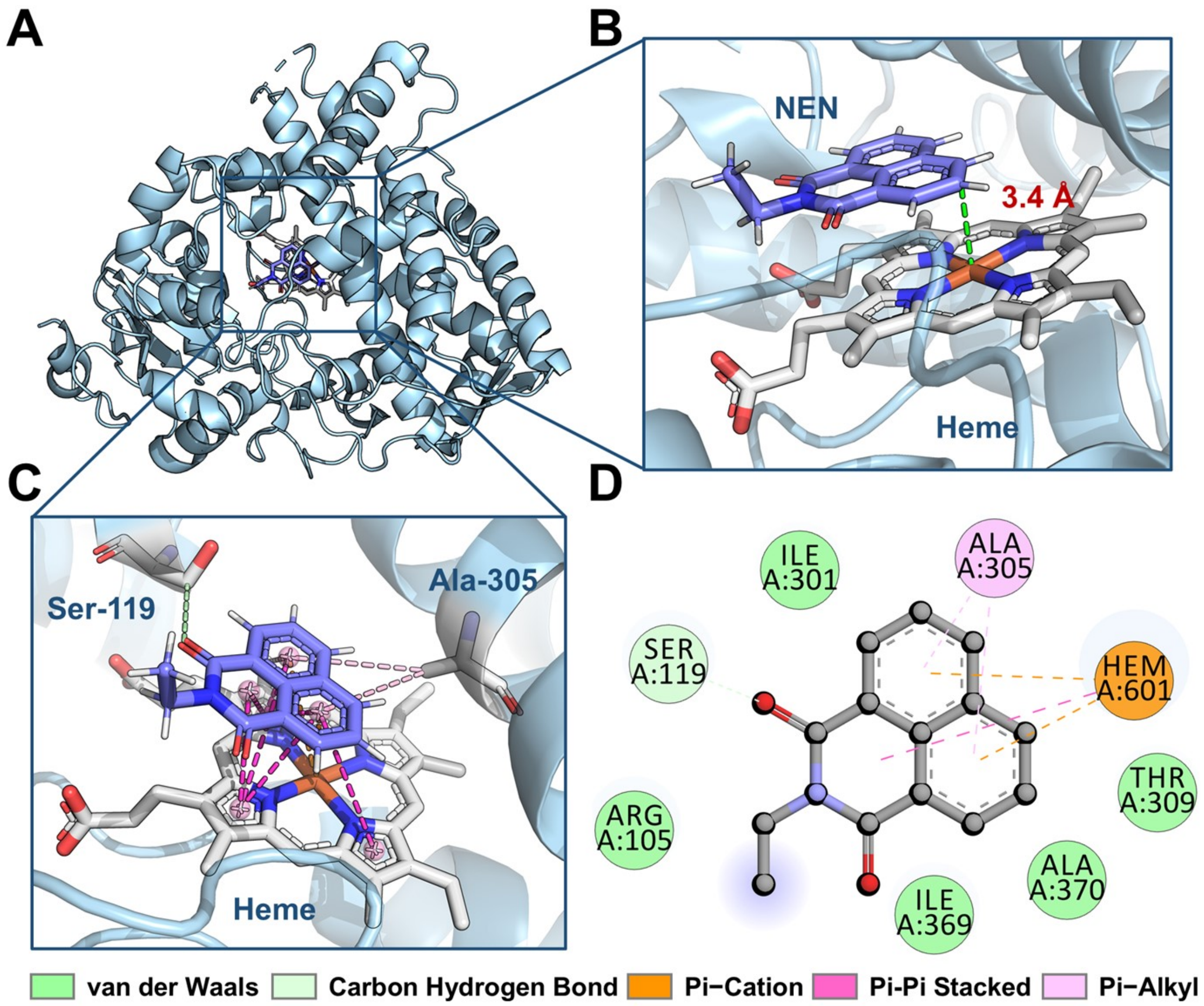

Fig. S43. Molecular docking simulations of **NEN** in the catalytic cavity of CYP3A4, including overview pose (A), catalytic distance (B), binding mode (C), and 2D interaction analysis (D).

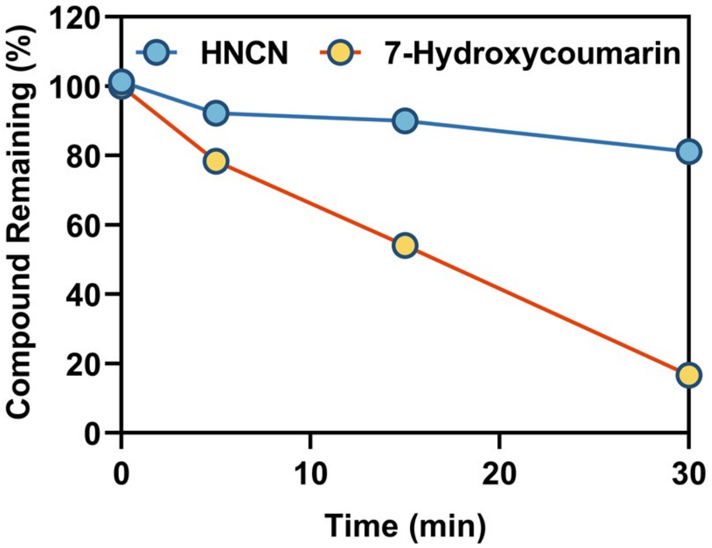

Fig. S44. Phase II metabolic stability of **HNCN** in HLMs.

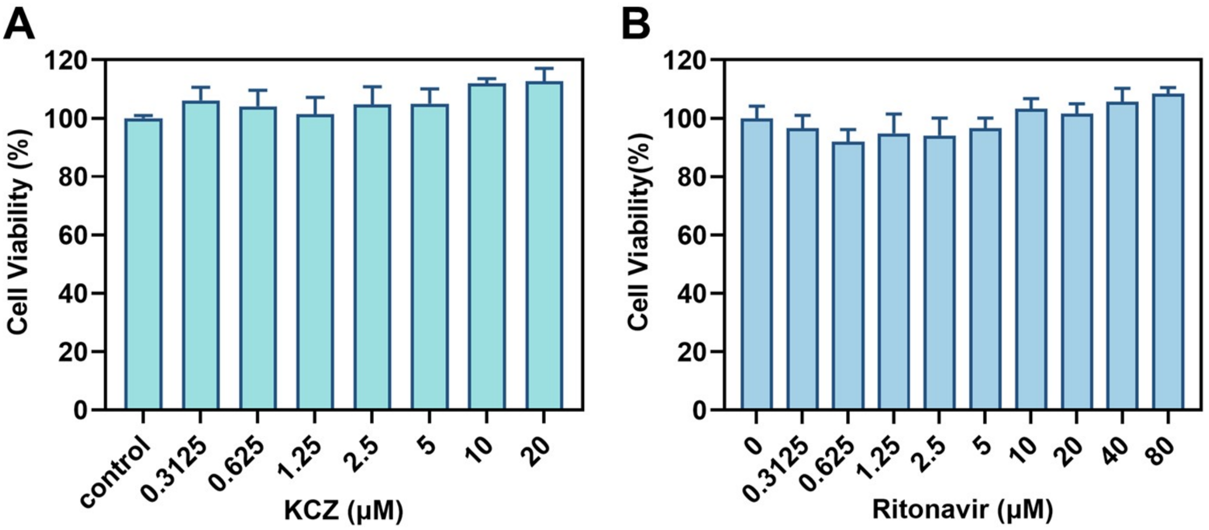

Fig. S45. Cytotoxicity assays of KCZ (A) and ritonavir (B) in Hep3B cells.

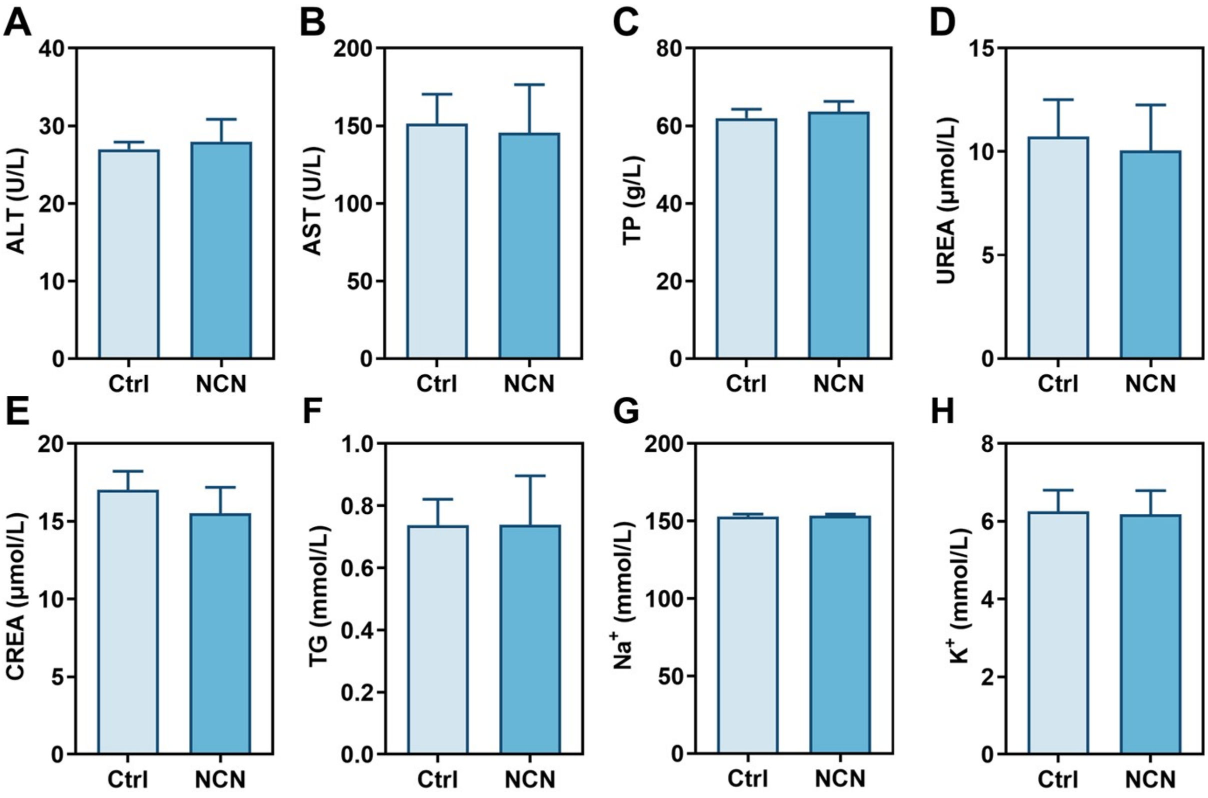

Fig. S46. Serum biochemical parameters of control and **NCN**-treated mice, including ALT, AST, total protein (TP), urea, creatinine, triglyceride (TG), Na^+, and K^+.

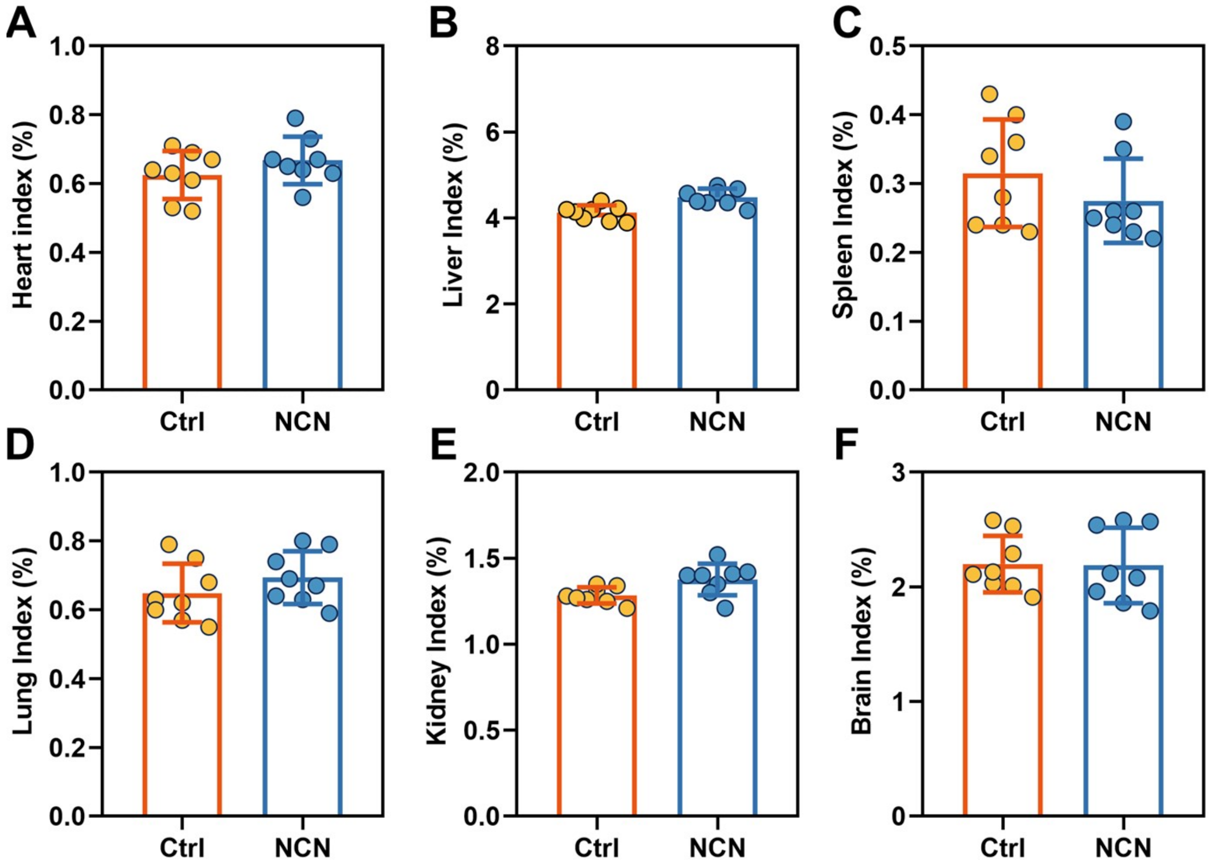

Fig. S47. The organ index in mice following treatment with **NCN** for 14 consecutive days.

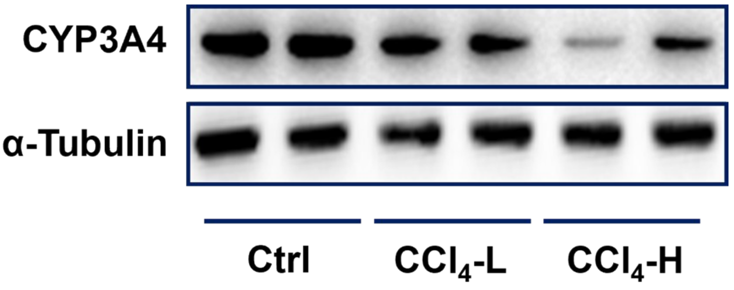

Fig. S48. The protein expression levels of CYP3A4 in the liver from healthy mice and CCl_4-induced liver damage mice.

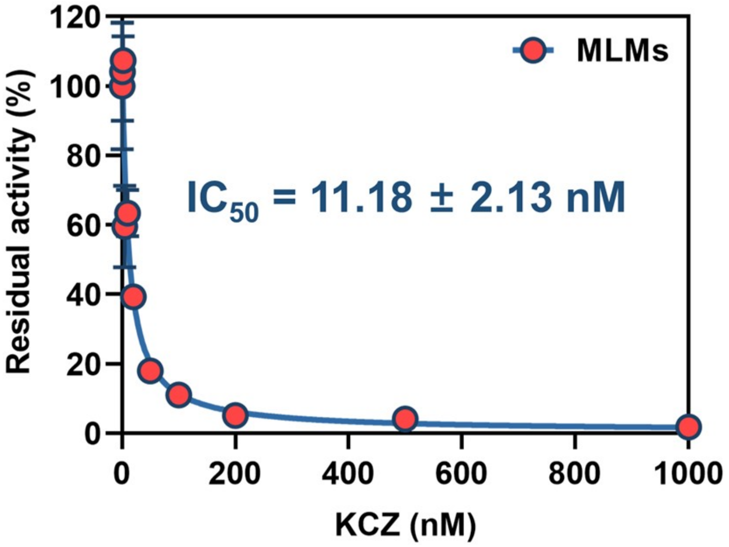

Fig. S49. Dose-inhibition curves of KCZ against CYP3A in MLMs.

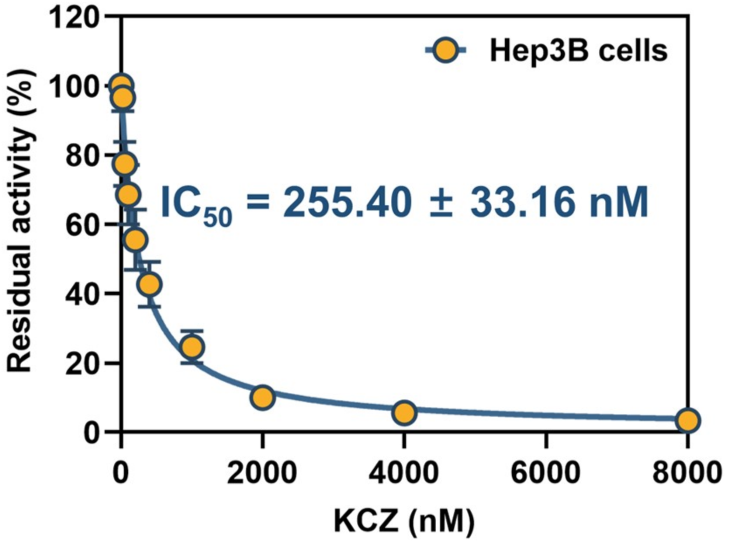

Fig. S50. Dose-inhibition curves of KCZ against CYP3A4 in Hep3B cells.

Table S1. The previously reported fluorogenic substrates for CYP3A4.

Probe	$\lambda_{ex}/\lambda_{em}$ (nm)	K_m (μM)	P-gp substrate	Biological applications	Ref.
**NEN**	450/558	59.8	Yes	Sensing CYP3A4 activity in HLM	(Ning et al. 2019)
**F8**	450/555	0.36	Yes	Sensing CYP3A4 activity in HLM<br>Imaging of CYP3A4 activity in Hepatocytes and liver slice.	(He et al. 2023)

Table S2. Physicochemical and drug-like properties of candidate probes predicted by SwissADME (http://www.swissadme.ch/).

Probe	Smiles	MW	Rotatable bonds	HBA	HBD	log*P*	tPSA (Å²)	Lipinski's rule of five
**1**	c1ccc2c3c1c(=O)n(c(=O)c3ccc2)CC(F)F	261.22	2	4	0	3.04	39.07	Yes
**2**	c1ccc2c3c1c(=O)n(c(=O)c3ccc2)CC(F)(F)F	279.21	2	5	0	3.31	39.07	Yes
**3**	c1ccc2c3c1c(=O)n(c(=O)c3ccc2)C1CC1	237.25	1	2	0	2.80	39.07	Yes
**4**	c1ccc2c3c1c(=O)n(c(=O)c3ccc2)C1CCCCC1	279.33	1	2	0	3.71	39.07	Yes
**5**	c1ccc2c3c1c(=O)n(c(=O)c3ccc2)CC1CC1	251.28	2	2	0	3.03	39.07	Yes
**6**	c1ccc2c3c1c(=O)n(c(=O)c3ccc2)CC1CCC1	265.31	2	2	0	3.37	39.07	Yes
**7**	c1ccc2c3c1c(=O)n(c(=O)c3ccc2)CC1(COC1)C	281.31	2	3	0	2.78	48.30	Yes
**8**	c1ccc2c3c1c(=O)n(c(=O)c3ccc2)C[C@H]1COCC1	281.31	2	3	0	2.72	48.30	Yes
**9**	c1ccc2c3c1c(=O)n(c(=O)c3ccc2)CCC1CC1	265.31	3	2	0	3.42	39.07	Yes
**10**	c1ccc2c3c1c(=O)n(c(=O)c3ccc2)CCN1CCCC1	294.35	3	3	0	2.82	42.31	Yes
**11**	c1ccc2c3c1c(=O)n(c(=O)c3ccc2)CCN1CCOCC1	310.35	3	4	0	2.23	51.54	Yes
**12**	c1ccc2c3c1c(=O)n(c(=O)c3ccc2)CCN1CCNCC1	309.36	3	4	1	1.92	54.34	Yes
**13**	c1ccc2c3c1c(=O)n(c(=O)c3ccc2)CCN1CCN(CC1)C	323.39	3	4	0	2.18	45.55	Yes
**14**	c1ccc2c3c1c(=O)n(c(=O)c3ccc2)Cc1sccc1	293.34	2	2	0	3.51	67.31	Yes
**15**	c1ccc2c3c1c(=O)n(c(=O)c3ccc2)Cc1occc1	277.27	2	3	0	2.84	52.21	Yes
**16**	c1ccc2c3c1c(=O)n(c(=O)c3ccc2)Cc1ocnc1	278.26	2	4	0	2.30	65.10	Yes
**17**	c1ccc2c3c1c(=O)n(c(=O)c3ccc2)Cc1cncs1	294.33	2	3	0	2.86	80.20	Yes
**18**	c1ccc2c3c1c(=O)n(c(=O)c3ccc2)Cc1cnc(s1)Cl	328.77	2	3	0	3.50	80.20	Yes
**19**	c1ccc2c3c1c(=O)n(c(=O)c3ccc2)Cc1sccn1	294.33	2	3	0	2.87	80.20	Yes
**20**	c1ccc2c3c1c(=O)n(c(=O)c3ccc2)Cc1ccc(cc1)CN1CCCCC1	384.47	4	3	0	4.24	42.31	Yes
**21**	c1ccc2c3c1c(=O)n(c(=O)c3ccc2)Cc1cnccn1	289.29	2	4	0	2.05	64.85	Yes
**22**	c1ccc2c3c1c(=O)n(c(=O)c3ccc2)CCNS(=O)(=O)c1ccc(cc1)C	394.44	5	5	1	3.03	93.62	Yes

Table S3. ADME/Tox properties of candidate probes predicted by SwissADME (http://www.swissadme.ch/).

Probe	Gastrointestinal absorption	P-gp substrate	CYP3A4 inhibitor
**1**	High	No	No
**2**	High	No	No
**3**	High	No	No
**4**	High	No	No
**5**	High	No	No
**6**	High	No	No
**7**	High	No	No
**8**	High	No	No
**9**	High	No	No
**10**	High	No	No
**11**	High	No	No
**12**	High	No	No
**13**	High	No	No
**14**	High	No	No
**15**	High	No	No
**16**	High	No	No
**17**	High	No	No
**18**	High	No	No
**19**	High	No	No
**20**	High	No	No
**21**	High	No	No
**22**	High	No	No

Table S4. Distance between the C-4 site of each candidate probe and the heme of CYP3A4.

Probe	Catalytic distance (Å)				
	3NXU	4I4G	4K9U	5TE8	5VC0
1	11.1	3.8	3.9	3.4	3.4
2	10.6	3.9	3.8	4.3	3.5
3	11.2	3.7	3.9	3.5	3.4
4	3.2	3.8	3.8	3.3	12.2
5	3.8	3.6	3.9	3.5	3.2
6	3.2	3.5	3.9	3.4	3.1
7	10.9	3.9	3.8	3.4	3.4
8	3.3	3.8	3.9	3.4	3.4
9	3.9	11.5	3.8	3.5	4.0
10	3.6	3.8	3.8	3.4	3.5
11	3.3	3.9	3.9	3.4	3.5
12	3.3	3.8	3.9	3.5	3.5
13	3.3	3.9	3.9	3.5	3.4
14	10.9	3.8	3.9	3.5	3.4
15	3.5	3.8	5.2	3.5	3.5
16	10.8	3.8	3.9	3.5	3.5
17	10.9	3.9	3.9	3.4	3.5
18	3.3	4.0	3.9	3.5	3.3
19	11.0	3.8	3.9	3.3	3.4
20	4.0	3.9	5.3	11.2	3.6
21	11.1	4.0	4.0	3.5	3.4
22	3.7	3.7	5.0	4.6	3.5

Table S5. The pharmacokinetic parameters of **NCN** when **NCN** was administered orally (20 mg/kg, *i.g.*, n = 5) and intravenously (2 mg/kg, *i.v.*, n = 5).

Administration	T_{max} (h)	C_{max} (ng/mL)	$AUC_{(0\text{-inf})}$ (ng/mL*h)	$t_{1/2}$ (h)	Oral bioavailability (%)
i.g.	2.00 ± 0.71	275.00 ± 65.00	1108.26 ± 208.24	1.79 ± 0.41	12.92
i.v.	0.083 ± 0.00	1862.34 ± 476.55	857.77 ± 119.78	2.59 ± 0.33	

Table S6. Comparison of **NCN** with previous probes (**NEN** and **F8**) in term of sensing performance and pharmacokinetic properties.

	NEN	F8	NCN
$\lambda_{ex}/\lambda_{em}$ (nm)	450/558	450/555	450/555
K_m (μM)	59.8	0.36	4.27
V_{max} (nmol/min/nmol)	2.18	–	10.01
Detection limit (nM)	–	0.1	0.03
Cell-membrane penetration (%)	0.99% (NCM460) 2.29% (Hep3B)	2.09% (NCM460) 4.82% (Hep3B)	23.76% (NCM460) 31.45% (Hep3B)
P-gp substrate	Yes	Yes	No
Application ranges	HLMs, hepatocytes, and zebrafish	HLMs, hepatocytes, liver slice, and mice	HLMs, hepatocytes, Isolated liver, and mice
Administration route	–	Intraperitoneal injection	Intragastric administration
Oral bioavailability	–	–	12.92%

References

He, R.J., Tian, Z.H., Huang, J., Sun, M.R., Wei, F., Li, C.Y., Zeng, H.R., Zhang, F., Guan, X.Q., Feng, Y., Meng, X.M., Yang, H., Ge, G.-B., 2023. Rationally Engineered CYP3A4 Fluorogenic Substrates for Functional Imaging Analysis and Drug–Drug Interaction Studies. Journal of Medicinal Chemistry 66(10), 6743-6755.

Lu, T., Chen, F., 2011. Multiwfn: A multifunctional wavefunction analyzer. Journal of Computational Chemistry 33(5), 580-592.

Ning, J., Wang, W., Ge, G., Chu, P., Long, F., Yang, Y., Peng, Y., Feng, L., Ma, X., James, T.D., 2019. Target Enzyme-Activated Two-Photon Fluorescent Probes: A Case Study of CYP3A4 Using a Two-Dimensional Design Strategy. Angew Chem Int Ed Engl 58(29), 9959-9963.

William Humphrey, A.D., and Klaus Schulten, 1996. VMD_ Visual molecular dynamics 14(2), 33-38.
